# Sphingolipidomic Profiling of Rat Serum by UPLC-Q-TOF-MS: Application to Rheumatoid Arthritis Study

**DOI:** 10.3390/molecules23061324

**Published:** 2018-05-31

**Authors:** Fanghui Qu, Hongyang Zhang, Min Zhang, Ping Hu

**Affiliations:** 1 School of Chemistry and Molecular Engineering, East China University of Science and Technology, Shanghai 200237, China; 13162300607@163.com; 2 Shanghai Key Laboratory of New Drug Design, School of Pharmacy, East China University of Science and Technology, Shanghai 200237, China; zhangm@ecust.edu.cn

**Keywords:** sphingolipidomic, UPLC-Q-TOF-MS, rat serum, rheumatoid arthritis, indomethacin intervention

## Abstract

Sphingolipids (SPLs) are biologically important molecules, but the structural diversity and complexity of SPLs brings significant analytical challenges for their study. In this paper, we have developed an UPLC-Q-TOF-MS-based sphingolipidomic approach for the comprehensive identification and quantification of SPLs in rat serum. A total of 120 SPLs covering seven subcategories were identified for the first time. Method validations including linearity, sensitivity, reproducibility, and recovery were also evaluated. This method was exemplarily applied to characterize the SPL alterations in rheumatoid arthritis (RA) rats and the intervention effects of indomethacin (IDM). Partial least squares-discriminant analysis (PLS-DA) showed that the model group was well separated from the control group, whereas the IDM-treated group exhibited a trend to recover the controls. Twenty-six significantly changed SPL markers were explored, and the levels of ceramides (Cers) and their metabolites were found to be reversed by IDM treatment. These results indicate that IDM exerts anti-arthritic effects through the suppression of Cer-mediated COX-2 activation and resulting PEG_2_ liberation. The present study demonstrates a promising potential of this method for the understanding of RA and the anti-arthritic mechanisms of relevant drugs.

## 1. Introduction

**Figure 1:**
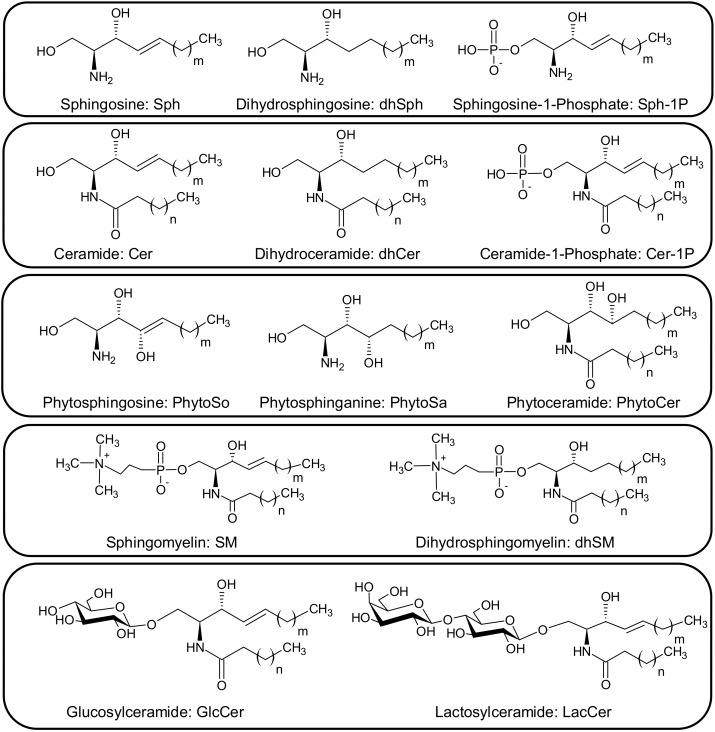
General structures, nomenclatures, and abbreviations for SPLs. *m*, carbon number of the sphingoid base backbone; *n*, carbon number of the fatty acid chain.

Sphingolipids (SPLs) represent the largest class of bioactive lipids involved in many aspects of cell structure, metabolism, and regulation [1]. Dysregulation of SPL homeostasis has been implicated in various diseases, including inflammation, cancer, obesity, and neurodegenerative disorders [2,3,4,5]. All SPLs share the common backbones of sphingoid bases, a set of aliphatic amino alcohols that contains sphingosine (So) and sphinganine (Sa) (Figure 1). As the simplest type, sphingoid bases can be amide-linked with a fatty acid to convert into ceramide (Cer). These basic SPLs can be further derivatized to: (i) phosphates, e.g., sphingosine-1-phosphate (S1P) and ceramide-1-phosphate (C1P); (ii) phosphosphingolipids, e.g., sphingomyelin (SM); and (iii) glycosphingolipids, e.g., glucosylceramide (GlcCer) and lactosylceramide (LacCer). The structural diversity and complexity of SPLs, due to the numerous variations in sphingoid backbones, fatty acid chains, and head groups, brings significant analytical challenges for their study. Therefore, the introduction of “sphingolipidomics” aims for comprehensive analysis of all SPLs present in a biological system, and is urgently required to understand the roles of SPLs in biology and disease [1].

Nowadays, liquid chromatography-mass spectrometry (LC-MS) has become the most powerful technique for sphingolipidomic analysis owing to its high sensitivity, specificity, and throughput capabilities [6]. Nevertheless, because of the extreme complexity of SPLs, there are still several difficulties in current LC-MS methods using triple-quadrupole mass spectrometry (QQQ-MS) in multiple reaction monitoring (MRM) mode [6,7]. A key limitation is that the existing methods for SPL profiling often suffer from isomeric, isobaric, or isotopic interferences, leading to a failure in detection of the low-abundance but biologically important SPLs. To solve this problem, the latest methodology developed by our group is mainly based on ultra-performance liquid chromatography coupled with quadrupole time-of-flight mass spectrometry (UPLC-Q-TOF-MS) [8]. UPLC provides sufficient separation of the interfering species. High resolution Q-TOF-MS offers accurate mass measurements at both MS and MS/MS level, permitting a reliable identification for subsequent quantification. This strategy appears promising for sphingolipidomic studies, including the discovery of disease biomarkers, as well as the evaluation of therapeutic effects of drug [8,9].

Rheumatoid arthritis (RA) is a chronic, destructive, and autoimmune disease characterized by inflammation in movable joints [10]. Growing evidence shows the significant role of SPLs in the pathogenesis of RA, and also demonstrates their potential as therapeutic targets for RA treatment [10,11]. However, until now, there has been no sphingolipidomic study focusing on this area. Herein, we firstly report an UPLC-Q-TOF-MS-based sphingolipidomic approach for the comprehensive identification and quantification of SPLs in rat serum. The developed method was fully validated and applied to RA study. Serum samples collected from rats in control, model, and indomethacin (IDM)-treated groups were comparatively analyzed. Partial least squares-discriminant analysis (PLS-DA) was further employed to identify the arthritis-related lipid alterations and explore the anti-arthritic mechanism of IDM.

## 2. Results and Discussion

### 2.1. Comprehensive Identification of SPLs in Rat Serum

**Figure 2:**
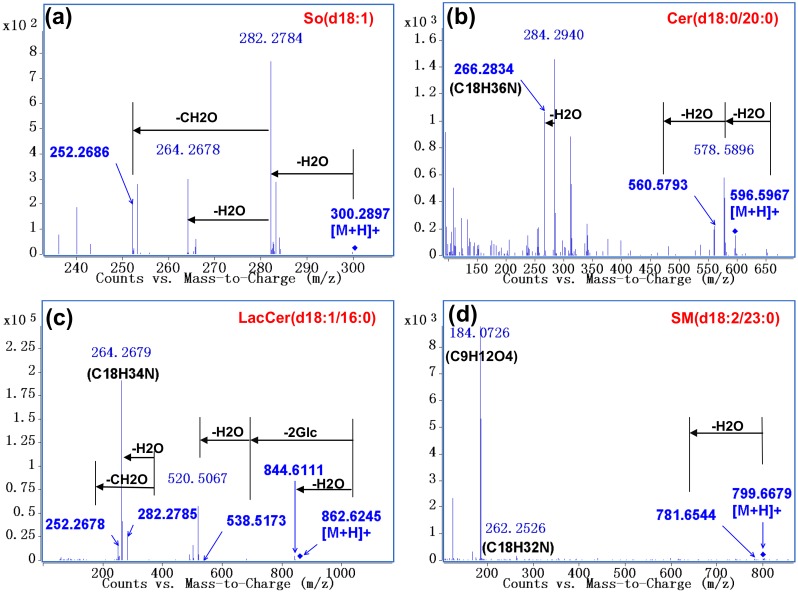
Characteristic MS/MS spectra of (**a**) So (d18:1); (**b**) Cer (d18:0/20:0); (**c**) LacCer (d18:1/16:0); and (**d**) SM (d18:2/23:0) by Q-TOF-MS analysis. So, sphingosine; Cer, ceramide; LacCer, lactosylceramide; SM, sphingomyelin.

UPLC-Q-TOF-MS is a useful analytical tool to separate and identify SPLs in complex matrices. The targeted-MS/MS analysis offered by Q-TOF-MS provides the accurate mass of both molecular and fragment ions, which are informative for SPL identification. Taking *m*/*z* 300.2897 (So), 596.5967 (Cer), 862.6245 (LacCer), and 799.6679 (SM) as examples, the fragmentation patterns of each species were summarized, as shown in Figure 2. The neutral loss of H_2_O and/or HCHO observed from MS/MS spectra corresponds to the hydroxyl group in sphingoid backbone. The characteristic ions specific to sphingoid backbone are decisive for the structural elucidation of SPLs, e.g., *m*/*z* 266.2834, 264.2679, and 262.2526 ions for the assignment of d18:0, d18:1, and d18:2 backbones, respectively [6,8]. Additionally, the abundant phosphocholine ion at *m*/*z* 184.0726 is regarded as the diagnostic fragment for the confirmation of SMs [6]. For complex glycosphingolipids, the neutral loss of sugar units reflects their sugar residue compositions, e.g., two 162 u gaps from the ions of *m*/*z* 862.6245 to 538.5173 suggesting the sequential loss of two glucosyl groups. Based on these fragmentation rules mentioned above, a total of 120 SPLs were identified in rat serum samples, covering seven subcategories (i.e., So and Sa, S1P, Cer, C1P, hexosylceramide (HexCer), LacCer, and SM) with various sphingoid and fatty acid chain compositions (Table 1 and Figure S1). It is worth mentioning that HexCer includes GlcCer and galactosylceramide (GalCer), which cannot be distinguished by this method.

**Figure 3:**
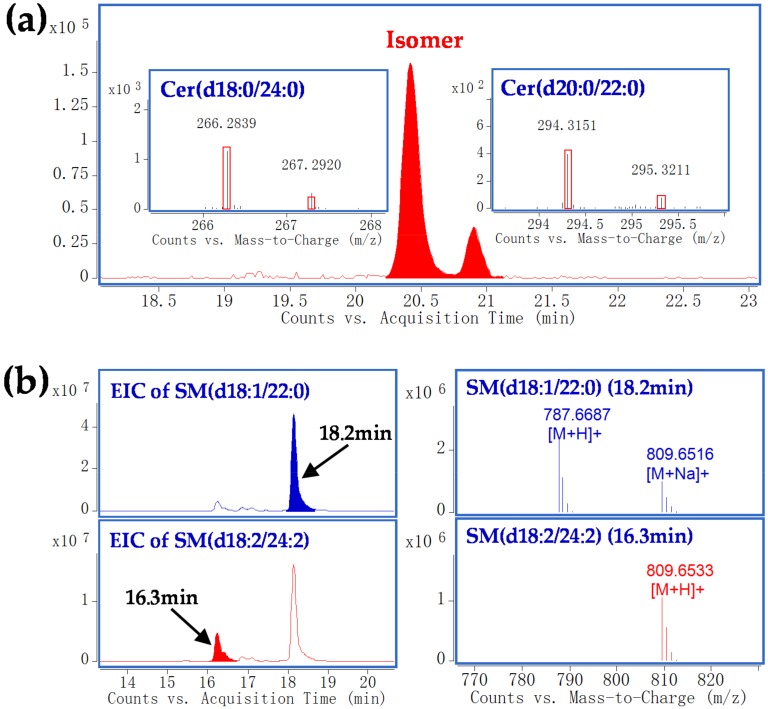
(**a**) Differentiation of SPL isomers by targeted-MS/MS analysis. Two peaks in EIC of *m*/*z* 652.6602 showed characteristic fragment ion corresponding to Cer (d18:0/24:0) (*m*/*z* 266.2839) and Cer (d20:0/22:0) (*m*/*z* 294.3151); (**b**) Differentiation of [M + H]^+^ of SM (d18:2/24:2) and [M + Na]^+^ of SM (d18:1/22:0) that with the same exact *m*/*z*. The “real” [M + H]^+^ of SM (d18:2/24:2) was assigned by the observation of its corresponding [M + Na]^+^ ion. Cer, ceramide; SM, sphingomyelin.

In the current analysis, MS signals might be interfered by isomeric or isobaric ions. A major interference in SPL identification is the isomeric species that have same molecular formula, thus, MS/MS data are necessary for discrimination. For example, the extracted ion chromatogram of *m*/*z* 652.6602 (mass accuracy within 5 ppm) yielded two Cer peaks at 20.3 and 20.9 min, respectively (Figure 3a). Further checking their MS/MS spectra revealed characteristic product ions corresponding to d18:0 (*m*/*z* 266.2839) and d20:0 (*m*/*z* 294.3151) backbones, providing evidence for the differentiation of the two isomers. Another issue is the isobaric interference from sodium adduct ions of SPLs. As seen in Figure 3b, the [M + H]^+^ ion of SM (d18:2/24:2) (calculated *m*/*z* 809.6533) coincides exactly with the *m*/*z* for [M + Na]^+^ ion of SM (d18:1/22:0) (calculated *m*/*z* 809.6516). In this case, the “real” [M + H]^+^ ion could be unambiguously assigned by the observation of its corresponding [M + Na]^+^ ion. Compared with other SPLs, identification of SMs is relatively difficult because they are more prone to form sodium adducts. It should be noticed that sufficient chromatographic separation aids in the elimination of isomeric/isobaric interferences and, therefore, facilitates accurate characterization and quantification of these species.

On the basis of optimized separation, linear regression models were constructed by plotting carbon number vs. retention time of SPLs sharing the same sphingoid backbone and unsaturated degree (Figure S2) [7,8]. Goodness of fit (*R*^2^ > 0.998 for each series) implies its capability for predicting retention behavior of the species given its chain composition, as well as for aiding in identification. This is also beneficial for the analysis of other biological samples enriched in SPLs, such as neural cells and tissues.

### 2.2. Method Validation for Quantitative Profiling

The established UPLC-Q-TOF-MS method was further validated for SPL quantification [7]. Aliquots of pooled serum from control rats were utilized for the method validation. Linearity was determined by spiking eight internal standards (IS) into samples prior to extraction at a series of spiked levels from 0.0167 to 25 µmol/L. Each sample was prepared in duplicate and measured twice. Calibration curves were constructed by linear regression. A wide dynamic range over three orders of magnitude was achieved for all IS with correlation coefficients (*R*^2^) better than 0.995. Limit of detection (LOD) and limit of quantification (LOQ) were defined as the lowest concentration when signal-to-noise ratio (S/N) of three and 10 were obtained, respectively. As shown in Table S1, the LOD and LOQ values of 8 IS ranged from 0.11–2.80 nmol/L and 0.36–9.33 nmol/L, respectively.

Intraday reproducibility was assessed by analyzing six replicates of the serum samples at different times within one day. For interday reproducibility, nine replicates of samples were measured during three consecutive days (each day three samples were prepared and analyzed). Endogenous SPLs belonging to different subcategories were randomly selected and relative standard deviations (RSDs) were used to evaluate the reproducibility. As seen in Figure S3, the RSDs of intraday and interday variations for eleven representative SPLs were lower than 4.6% and 8.8%, respectively. Recovery was verified by comparing peak areas of IS spiked into samples before and after SPL extraction at three different levels (5, 50, and 500 pmol). At each spiked level, triplicates of samples were prepared and analyzed. The extraction recoveries of all IS were between 91.3% and 108.5% with RSDs less than 12.5% (Table S2). All above results demonstrated that the proposed method was acceptable for routine SPL profiling.

### 2.3. Application in Study of RA and IDM Intervention in Rats

**Figure 4:**
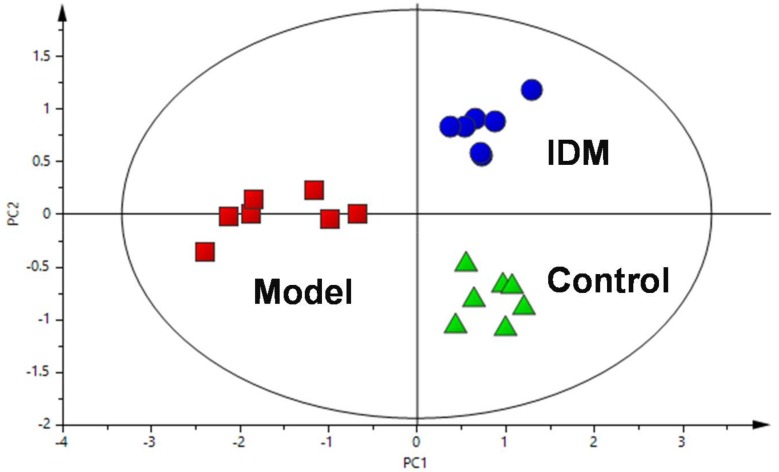
PLS-DA scores plot of rat serum data in control group (green triangles, *n* = 7), model group (red squares, *n* = 7), and IDM group (blue dots, *n* = 7).

**Figure 5:**
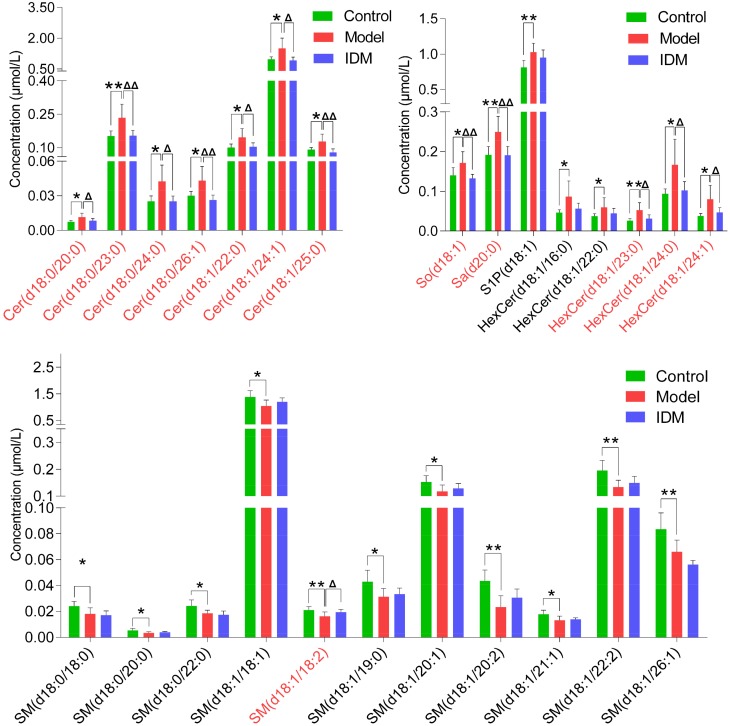
Altered levels of SPL markers in the control, model and IDM-treated rats. Data are represented as mean ± SD (*n* = 7 in each group). *, Δ *p* < 0.05 and **, ΔΔ *p* < 0.01 from one-way ANOVA analysis. The markers colored in red indicate their levels were significantly reversed by IDM treatment. Cer, Ceramide; So, sphingosine; Sa, sphinganine; S1P, sphingoid-1-phosphate; HexCer, hexosylceramide; SM, sphingomyelin.

To show the potential of the developed method, sphingolipidomic profiling was applied to rat serum samples obtained from control, model, and IDM-treated groups. A PLS-DA method was then used to visualize the lipid variations among these samples (Figure 4). The score plot showed a clear separation between the control and model groups in the first dimension, indicating a distinct arthritis induced by CFA. After the treatment of IDM, the serum SPL profiles were found to move away from the models and exhibit a tendency to restore to the controls, reflecting a protective effect of IDM against RA. Additionally, alterations of differential SPLs contributing to the classification were also investigated via one-way ANOVA analysis. Twenty-six SPLs were, therefore, selected as potential inflammatory markers, as shown in Figure 5.

**Figure 6:**
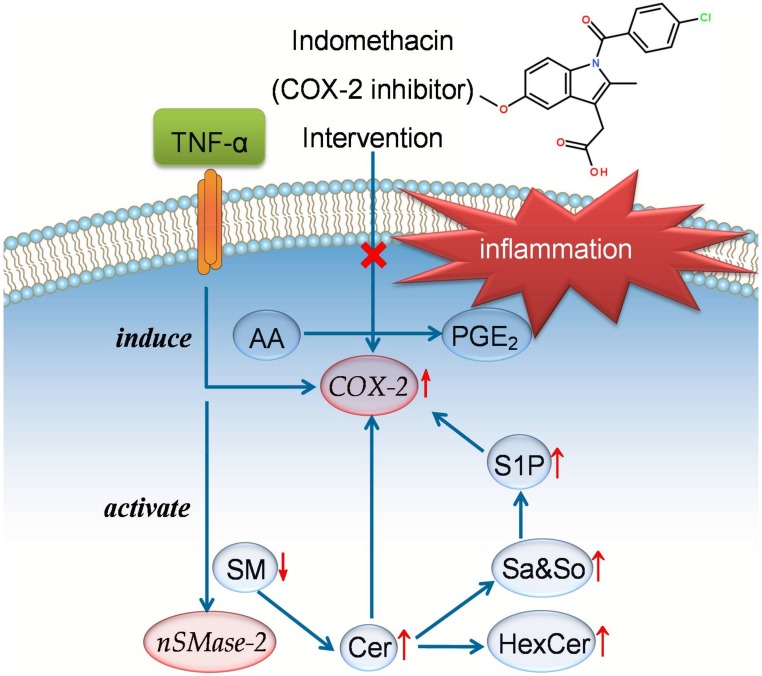
Proposed mechanistic pathways for the inflammation in RA and anti-arthritic effects of IDM. Upward and downward arrows represent up-regulation and down-regulation, respectively. Abbreviations: TNF-α, tumor necrosis factor-α; AA, arachidonic acid; PGE_2_, prostaglandin E_2_; COX-2, cyclooxygenase-2; SM, sphingomyelin; *n*SMase, neutralsphingomyelinase; Cer, ceramide; Sa&So, sphinganine and sphingosine; S1P, sphingosine-1-phosphate; HexCer, hexosylceramide.

In the identified markers, Cers, HexCers, So, Sa, and S1P displayed up-regulation in the arthritic model rats, while SMs were down-regulated. These results suggest that an impaired SPL metabolism is involved in RA (Figure 6). During inflammation, the key cytokine TNF-α can induce the overexpression of cyclooxygenase-2 (COX-2) and promote the release of pro-inflammatory prostaglandin E_2_ (PGE_2_) [2,12]. TNF-α also stimulates specific SPL metabolic enzymes, including ceramidase (CDase) and sphingosine kinase (SphK), to increase the levels of Cers and S1P [13,14]. As the structural core of SPLs, Cer is thought to have crucial roles in the development of inflammation Recent evidence has revealed that Cer accumulation enhances the COX-2 expression and PEG_2_ liberation, giving rise to various inflammatory diseases [15,16]. In addition to Cer, S1P is also able to activate the COX-2 enzyme and trigger the production of PEG_2_ [14]. SphK activation and high S1P levels have been reported in the synovial fluids of patients with RA [17]. Therefore, our findings of significant increment of Cers (together with their metabolites HexCers or So) and S1P in arthritic rats indicate an involvement of TNF-α-mediated inflammation in RA. Furthermore, neutral sphingomyelinase-2 (*n*SMase-2), which hydrolyzes SM into Cer, plays an important role in the inflammatory process. This enzyme has been shown to be activated by TNF-α, resulting in a reduction in SMs and of elevation of Cers observed in this study [18]. We here propose that the increased activities of *n*SMase-2 and accelerated SM hydrolysis are also contributed to the pathogenesis of RA.

The present work further demonstrated that IDM administration could ameliorate the CFA-induced RA in rats. IDM is a common nonsteroidal anti-inflammatory drug that has been effectively used in the management of arthritis disease. Its mechanism of action is through inhibition of COX-2 activity to block the biosynthesis of PEG_2_ [19]. As seen in Figure 5, the levels of specific SPL markers, including Cers and their metabolites, were significantly reversed by IDM treatment. These results indicate that IDM exerts anti-arthritic effects by down-regulating Cer synthesis, leading to the suppression of COX-2 activation and resulting PEG_2_ production in RA (Figure 6). In contrast, no significant intervention effects were observed for most SMs, suggesting that IDM has less impact on the *n*SMase-2-activated SM hydrolysis pathway. Our sphingolipidomic data, thus, affirm and extend previous research on the therapeutic actions of IDM.

## 3. Materials and Methods

### 3.1. Chemicals and Materials

The IS mixture (25 µmol/L for each SPLs in ethanol, catalog LM-6005) was purchased from Avanti Polar Lipids (Alabaster, AL, USA). It was composed of uncommon SPLs including So (d17:1), Sa (d17:0), S1P (d17:1), Cer (d18:1/12:0), C1P (d18:1/12:0), GlcCer (d18:1/12:0), and LacCer (d18:1/12:0), and SM (d18:1/12:0). Complete Freund’s adjuvant (CFA), ammonium acetate (NH_4_Ac) and formic acid (HCOOH) were purchased from Sigma-Aldrich (St. Louis, MO, USA). LC-MS-grade methanol (MeOH), chloroform (CHCl_3_), and isopropanol (IPA) were purchased from Merck (Darmstadt, Germany). Ultrapure water (18.2 MΩ) was purified with a Milli-Q system (Millipore, Burlington, MA, USA). All other chemicals used were of analytical grade. Indomethacin (IDM) was obtained from Shanghai Shyndec Pharmaceutical Co., Ltd. (Shanghai, China).

### 3.2. Animal Experiment

All experimental procedures were approved by the Ethics Committee of the Laboratory Animal Center of East China University of Science and Technology (project code: 20160917-2, approved date: 17 September, 2016). A total of 21 male SD rats (180 ± 10 g) were purchased from Shanghai SLAC Laboratory Animal Co., Ltd. (Shanghai, China). All animals were housed in an air-conditioned room at temperature of 25 ± 1 °C, relative humidity of 50 ± 10%, and 12 h dark/light cycle. The rats were fed with certified standard chow and tap water ad libitum. After one week of acclimation, rats were randomly divided into three groups as follows: the control, model, and IDM groups (*n* = 7 in each group). The model rats and IDM treated rats were injected intradermally with CFA (0.1 mL) for 20 days to induce arthritis. The control rats received an equal amount of physiological saline during the whole experiment. From days 11 to 20, the IDM group was injected intraperitoneally with IDM at a dose of 3 mg/kg body weight. At the end of the period, all animals were sacrificed and the sera were obtained for the following analysis.

### 3.3. Sample Pretreatment

The extraction of rat serum SPLs was according to our established procedures with minor modifications [8]. Briefly, 0.75 mL of MeOH:CHCl_3_ (2:1, *v*/*v*) and 10 µL of IS (each of 50 pmol amount) were added into 100 µL of rat serum. After sonication for 30 s, the mixture was incubated at 48 °C for 12 h. After cooling, 75 µL of KOH (1 mol/L in MeOH) was added and then incubated at 37 °C for 2 h. After cooling to room temperature, 3 µL of acetic acid was added to neutralize the mixture. The single-phase mixture was centrifuged and the supernatants were collected. The residue was re-extracted with 1 mL of MeOH:CHCl_3_ (1:2, *v*/*v*), centrifuged, and the supernatants were combined. The residue was dissolved in 0.4 mL of MeOH:CHCl_3_ (2:1, *v*/*v*) and 1 mL of CHCl_3_ and 2 mL of H_2_O was added followed by vortex mixing for 1 min. After centrifugation, the lower phase extract was collected and incorporated. The upper phase fraction was re-extracted with 1 mL of CHCl_3_, which was also combined with the extract. After extraction, the extract was evaporated to dryness under a gentle stream of nitrogen. The dried residue was reconstituted in 150 µL of MeOH, sonicated for 30 s, and filtered through a 0.22 µm syringe filter (Millipore, Burlington, MA, USA) into vials (Agilent, Santa Clara, CA, USA) for UPLC-MS analysis.

### 3.4. Chromatography and Mass Spectrometry

An optimized UPLC-Q-TOF-MS condition established by our group was employed with several revisions [8]. Briefly, chromatographic separation was performed on a Zorbax Eclipse Plus C18 column (100 × 2.1 mm, 1.8 µm, Agilent, Santa Clara, CA, USA) using an Agilent 1290 UPLC system (Santa Clara, CA, USA), equipped with a binary solvent delivery system and a high-performance auto-sampler. The mobile phase consisted of (A) MeOH:H_2_O:HCOOH (60:40:0.2, *v*/*v*/*v*) and (B) MeOH:IPA:HCOOH (10:90:0.2, *v*/*v*/*v*), both containing 10 mM NH_4_Ac. The gradient was as follows (flow rate of 0.35 mL/min): 0–3 min, 0% to 10% B; 3–5 min, 10% to 40% B; 5–5.3 min, 40% to 55% B; 5.3–8 min, 55% to 60% B; 8–8.5 min, 60% to 80% B; 8.5–10.5 min, 80% to 80% B; 10.5–16 min, 80% to 90% B; 16–19 min, 90% to 90% B; 19–22 min, 90% to 100% B; 22–26 min, 100% to 100% B. The column temperature was maintained at 40 °C and the injection volume was 2 µL.

Both qualitative and quantitative analysis was carried out using an Agilent 6530 Q-TOF mass spectrometer (Santa Clara, CA, USA), equipped with an electrospray ionization (ESI) source. The instrument was operated under 4 GHz mode to obtain high resolution data. ESI conditions were as follows: positive ion mode, capillary voltage 3500 V, nebulizer 35 psi, drying gas 10 L/min, gas temperature 350 °C, skimmer voltage 65 V, octopole RF voltage 750 V, fragmentor voltage 150 V. The collision energies (CEs) of targeted-MS/MS analysis were set at 10, 20, and 40 eV, respectively. Mass spectra were recorded over a range of *m*/*z* 50–1500. A reference solution was sprayed as continuous calibration using the following reference masses: *m*/*z* 121.0509 and 922.0098. All MS and MS/MS data was processed with MassHunter Workstation Software (version B.06.00, Agilent, Santa Clara, CA, USA).

### 3.5. Data Processing and Statistics

Agilent MassHunter Workstation software (version B.06.00) was employed for data processing. Identification of SPLs in rat serum were based on their accurate MS and MS/MS data. Relative quantification was carried out under MS mode and peak areas of the extracted ion chromatograms (EICs) for each SPL were integrated. The quantitative results were obtained by IS normalization and calculated as follows: Concentration of target SPL (µmol/L) = (Area of target SPL/Area of corresponding IS) × spiked concentration of IS. Eight IS corresponding to each subcategory are listed in Table 1. All quantitative data were converted to Microsoft Excel format and imported into SIMCA-P+ 13.0 software (Umetrics, Umea, Sweden) for PLS-DA analysis. Statistical tests for identifying differentially expressed SPLs were performed with SPSS software (version 22.0, SPSS Inc., Chicago, IL, USA). Group variations were compared using one-way ANOVA analysis with a critical *p* value set to 0.05.

## 4. Conclusions

In summary, an UPLC-Q-TOF-MS-based sphingolipidomic approach was developed for the comprehensive identification and quantification of SPLs in rat serum. The benefits from the optimized LC-MS conditions, isomeric/isobaric interferences were reduced and a total of 120 SPLs covering seven subcategories were identified. The method was validated and demonstrated good reproducibility (overall RSDs ≤ 8.8%) and accuracy (overall recoveries ≥ 91.3%) with a wide linear range. This method was then applied to investigate the SPL alterations in RA rats and the intervention effects of IDM. PLS-DA analysis showed a clear separation of the model and control groups, while the IDM group exhibited a trend to recover to the controls. Twenty-six significantly changed SPL markers were explored, and levels of Cers and their metabolites were found to be reversed by IDM treatment. The therapeutic effects of IDM may be attributed to the inhibition of Cer-mediated COX-2 activation and resulting PEG_2_ liberation in RA. All of the above results show the promising potential of this method for understanding RA and the anti-arthritic mechanisms of relevant drugs.

## Figures and Tables

**Table 1:** Identification of SPLs in rat serum by using UPLC-Q-TOF-MS.

Class ^1^	No.	Name ^2^	Formula	RT (min)	[M + H]^+^ *m*/*z*	Score	MS/MS Fragments (*m*/*z*)
So&Sa	1	Sa (d14:0)	C14H31NO2	2.26	246.2422	99.89	228.2315, 210.2193, 198.2673
	2	So (d14:1)	C14H29NO2	2.60	244.2271	99.86	226.2156,208.1987, 196.2034
	3	So (t14:2)	C14H27NO3	4.45	258.2064	82.76	240.1939, 222.1958
	4	So (d15:1)	C15H31NO2	4.74	258.2428	99.74	240.2315, 222.2216, 210.2192
	5	Sa (d16:0)	C16H35NO2	5.08	274.2739	99.91	256.2627, 238.2575
	6	So (d16:1)	C16H33NO2	4.92	272.2584	86.47	254.2476, 236.2378
	7	Sa (t16:0)	C16H35NO3	5.12	290.2677	99.91	272.2571, 254.2487, 242.2472
	8	Sa (d18:0)	C18H39NO2	6.15	302.3053	98.75	284.2943, 266.2811
	9	So (d18:1)	C18H37NO2	9.85	300.2897	99.56	282.2796, 264.2728, 252.2642
	10	So (d18:2)	C18H35NO2	9.40	298.2745	85.01	280.2625, 262.2540
	11	Sa (d19:0)	C19H41NO2	11.91	316.3209	99.22	298.3109
	12	So (d19:1)	C19H39NO2	10.91	314.3041	99.31	296.2927
	13	Sa (d20:0)	C20H43NO2	13.84	330.3363	90.41	312.3268, 294.3189
	14	Sa (d24:0)	C24H51NO2	9.73	386.3950	99.34	368.3831, 350.3687
	[IS-1]	Sa (d17:0)	C17H37NO2	5.62	288.2872	-	270.2790, 252.2685, 240.2682
	[IS-2]	So (d17:1)	C17H35NO2	7.41	286.2749	-	268.2631, 250.2519, 238.2529
S1P	15	S1P (d18:1)	C18H38NO5P	6.93	380.2561	99.89	282.2864, 264.2709
	[IS-3]	S1P (d17:1)	C11H36NO5P	7.89	366.2399	-	348.2312, 268.2618, 250.2532
Cer	16	Cer (d16:1/23:0)	C39H77NO3	17.11	608.5976	92.16	590.5851, 572.5759, 254.2505, 236.2367
	17	Cer (d17:1/24:1)	C41H79NO3	16.89	634.6133	85.39	616.6008, 598.5886, 268.2633, 250.2521
	18	Cer (d18:0/16:0)	C34H69NO3	14.03	540.5344	90.32	522.5218, 504.5128, 302.3115, 284.2943, 266.2829
	19	Cer (d18:0/20:0)	C38H77NO3	16.88	596.5967	94.77	578.5896, 560.5793, 284.2940, 266.2834
	20	Cer (d18:0/21:0)	C39H79NO3	17.70	610.6133	94.78	592.6011, 266.2884
	21	Cer (d18:0/22:0)	C40H81NO3	18.41	624.6289	99.59	606.6167, 588.6131, 302.3045, 284.2946, 266.2837
	22	Cer (d18:0/23:0)	C41H83NO3	19.36	638.6448	97.15	620.6309, 602.6203, 302.3067, 284.2946, 266.2843, 254.2850
	23	Cer (d18:0/24:0)	C42H85NO3	20.30	652.6596	98.57	634.6530, 616.6360, 302.3034, 284.2964, 266.2846, 254.2844
	24	Cer (d18:0/24:1)	C42H83NO3	18.61	650.6441	98.55	632.6337, 614.6224, 302.3030, 284.2950, 266.2840, 254.2840
	25	Cer (d18:0/26:1)	C44H87NO3	20.53	678.6751	84.92	660.6634, 284.2932, 266.2848
	26	Cer (d18:1/14:0)	C32H63NO3	12.00	510.4881	78.69	492.4790, 474.4692, 282.2814, 264.2651
	27	Cer (d18:1/16:0)	C34H67NO3	13.61	538.5191	99.81	520.5074, 502.4899, 282.2789, 264.2684, 252.2676
	28	Cer (d18:1/17:2)	C35H65NO3	16.61	548.5037	98.94	264.2651
	29	Cer (d18:1/17:3)	C35H63NO3	12.96	546.4881	96.54	282.2838, 264.2675
	30	Cer (d18:1/18:0)	C36H71NO3	14.96	566.5507	97.65	548.5396, 282.2766, 264.2681, 252.2659
	31	Cer (d18:1/20:0)	C38H75NO3	16.38	594.5820	89.81	576.5703, 558.5569, 546.5640, 282.2792, 264.2676, 252.2668
	32	Cer (d18:1/20:4)	C38H67NO3	13.98	586.5167	67.08	300.2854, 282.2756, 264.2686
	33	Cer (d18:1/21:0)	C39H77NO3	17.01	608.5976	95.73	282.2768, 264.2670, 252.2686
	34	Cer (d18:1/22:0)	C40H79NO3	17.81	622.6133	99.51	604.6030, 586.5865, 300.2959, 282.2779, 264.2684, 252.2680
	35	Cer (d18:1/23:0)	C41H81NO3	18.66	636.6285	99.56	618.6171, 600.6080, 282.2790, 264.2683, 252.2682
	36	Cer (d18:1/23:1)	C41H79NO3	17.11	634.6133	84.89	616.6008, 598.5886, 282.2802, 264.2681, 252.2660
	37	Cer (d18:1/24:0)	C42H83NO3	19.59	650.6441	99.24	632.6343, 614.6247, 602.6231, 282.2789, 264.2678, 252.2682
	38	Cer (d18:1/24:1)	C42H81NO3	18.31	648.6299	91.22	630.6188, 612.6087, 282.2789, 264.2682, 252.2684
	39	Cer (d18:1/25:0)	C43H85NO3	20.07	664.6588	92.95	646.6410, 628.6374, 300.2882, 282.2786, 264.2682, 252.2680
	40	Cer (d18:1/26:3)	C42H77NO3	17.81	644.5934	90.90	282.2805, 264.2683
	41	Cer (d18:2/16:0)	C34H65NO3	12.71	536.5037	70.02	518.4939, 500.4770, 280.2648, 262.2522, 250.2529
	42	Cer (d18:2/18:2)	C36H65NO3	13.63	560.4967	90.73	542.4881, 280.2661, 262.2545
	43	Cer (d18:2/22:0)	C40H77NO3	16.79	620.5976	98.38	602.5861, 584.5741, 298.2758, 280.2635, 262.2526, 250.2515
	44	Cer (d20:0/16:0)	C36H73NO3	12.67	568.5663	97.20	312.3264, 294.3158
	45	Cer (d20:0/22:0)	C42H85NO3	20.91	652.6602	99.80	634.6507, 616.6358, 312.3241, 294.3116
	46	Cer (t18:0/16:0)	C34H69NO4	12.91	556.5299	81.00	502.4892, 300.2886, 282.2773, 264.2704
	47	Cer (t18:0/19:2)	C37H71NO5	15.96	610.5405	99.43	282.2797, 264.2680
	[IS-4]	Cer (d18:1/12:0)	C30H59NO3	10.38	482.4564	-	464.4507, 282.2779, 264.2682, 252.2670
C1P	48	C1P (d18:0/16:0)	C34H70NO6P	12.21	620.5014	97.60	266.2896
	49	C1P (d18:1/12:3)	C30H54NO6P	9.17	556.3733	90.34	264.2703
	50	C1P (d18:1/16:0)	C34H68NO6P	11.41	618.4866	97.06	264.2682
	51	C1P (d18:1/16:2)	C34H64NO6P	11.09	614.4544	91.65	264.2693
	52	C1P (d18:1/18:0)	C36H72NO6P	14.84	646.5187	76.43	282.2779, 264.2691
	53	C1P (d18:1/26:0)	C44H88NO6P	25.49	758.6422	86.48	264.2698
	54	C1P (d18:2/14:1)	C32H60NO6P	9.67	586.4231	96.64	262.2576
	[IS-5]	C1P (d18:1/12:0)	C30H60NO6P	9.61	562.4226	-	544.4109, 464.4442, 446.4342, 282.2719, 264.2677
HexCer ^3^	55	HexCer (d18:0/20:0)	C44H87NO8	13.19	758.6504	99.76	596.5976, 578.5845, 284.2936, 266.2851
	56	HexCer (d18:1/16:0)	C40H77NO8	11.65	700.5722	90.23	520.4977, 502.4891, 282.2819, 264.2683, 252.2643
	57	HexCer (d18:1/20:0)	C44H85NO8	14.31	756.6328	98.50	738.6248, 576.5731, 282.2748, 264.2694, 252.2673
	58	HexCer (d18:1/22:0)	C46H89NO8	15.95	784.6661	98.10	766.6539, 622.6147, 604.6016, 586.5895, 282.2804, 264.2681, 252.2644
	59	HexCer (d18:1/23:0)	C47H91NO8	16.60	798.6817	93.90	780.6693, 618.6176, 600.6079, 282.2786, 264.2683, 252.2675
	60	HexCer (d18:1/24:0)	C48H93NO8	17.27	812.6972	90.51	794.6858, 632.6339, 614.6225, 282.2791, 264.2683, 252.2681
	61	HexCer (d18:1/24:1)	C48H91NO8	16.05	810.6786	87.89	630.6161, 612.6090, 282.2784, 264.2686, 252.2670
	[IS-6]	HexCer (d18:1/12:0)	C36H69NO8	9.98	644.5093	-	626.4985, 464.4457, 446.4346, 282.2790, 264.2683, 252.2684
LacCer	62	LacCer (d18:0/22:0)	C52H101NO13	14.39	948.7346	98.47	624.6283, 606.6159, 284.2937, 266.2884
	63	LacCer (d18:1/16:0)	C46H87NO13	12.02	862.6245	99.73	844.6111, 538.5173, 520.5067, 282.2785, 264.2679, 252.2678
	64	LacCer (d18:1/22:0)	C52H99NO13	15.73	946.7177	92.24	928.7105, 928.7105, 604.6023, 586.5924, 282.2796, 264.2682, 252.2669
	65	LacCer (d18:1/24:0)	C54H103NO13	15.14	974.7502	99.16	956.7359, 794.6845, 632.6315, 614.6225, 282.2775, 264.2675, 252.2685
	66	LacCer (d18:1/24:1)	C54H101NO13	15.86	972.7346	98.93	954.7245, 792.6653, 630.6171, 612.6074, 282.2782, 264.2680, 252.2672
	[IS-7]	LacCer (d18:1/12:0)	C42H79NO13	9.78	806.5621	-	788.5510, 626.5002, 464.4464, 446.4358, 264.2686
SM	67	SM (d16:1/14:0)	C35H71N2O6P	11.53	647.5109	98.44	236.2362, 184.0737
	68	SM (d17:0/18:0)	C40H83N2O6P	15.14	719.6062	96.03	252.2691, 184.0731
	69	SM (d18:0/14:0)	C37H77N2O6P	13.10	677.5592	98.82	266.2830, 184.0729
	70	SM (d18:0/16:0)	C39H81N2O6P	14.43	705.5905	98.83	266.2810, 184.0727
	71	SM (d18:0/18:0)	C41H85N2O6P	15.85	733.6218	95.37	266.2899, 184.0736
	72	SM (d18:0/20:0)	C43H89N2O6P	17.30	761.6531	95.46	266.2787,184.0725
	73	SM (d18:0/22:0)	C45H93N2O6P	18.87	789.6844	99.17	266.2881, 184.0725
	74	SM (d18:0/24:0)	C47H97N2O6P	20.75	817.7157	99.36	266.2808, 184.0724
	75	SM (d18:1/14:0)	C37H75N2O6P	12.59	675.5436	99.00	264.2671, 184.0729
	76	SM (d18:1/16:0)	C39H79N2O6P	13.82	703.5755	99.19	264.2661, 184.0728
	77	SM (d18:1/16:3)	C39H73N2O6P	12.59	697.5279	97.13	264.2659, 184.0728
	78	SM (d18:1/17:0)	C40H81N2O6P	14.53	717.5904	98.69	264.2618, 184.0728
	79	SM (d18:1/18:0)	C41H83N2O6P	15.24	731.6062	98.06	264.2685, 184.0729
	80	SM (d18:1/18:1)	C41H81N2O6P	14.21	729.5926	86.17	264.2614, 184.0726
	81	SM (d18:1/18:2)	C41H79N2O6P	14.41	727.5737	99.30	264.2659, 184.0727
	82	SM (d18:1/18:3)	C41H77N2O6P	13.82	725.5569	94.58	264.2608, 184.0725
	83	SM (d18:1/19:0)	C42H85N2O6P	15.98	745.6218	99.44	264.2675, 184.0729
	84	SM (d18:1/19:1)	C42H83N2O6P	14.92	743.6064	97.01	264.2699, 184.0730
	85	SM (d18:1/20:0)	C43H87N2O6P	16.69	759.6361	91.31	264.2691, 184.0729
	86	SM (d18:1/20:1)	C43H85N2O6P	15.65	757.6218	99.58	264.2687, 184.0725
	87	SM (d18:1/20:2)	C43H83N2O6P	14.52	755.6062	98.54	264.2703, 184.0733
	88	SM (d18:1/20:3)	C43H81N2O6P	15.24	753.5905	91.53	264.2691, 184.0728
	89	SM (d18:1/21:0)	C44H89N2O6P	17.45	773.6531	99.62	264.2663, 184.0731
	90	SM (d18:1/21:1)	C44H87N2O6P	16.18	771.6367	98.99	264.2676, 184.0729
	91	SM (d18:1/22:0)	C45H91N2O6P	18.20	787.6682	89.66	264.2738, 184.0729
	92	SM (d18:1/22:1)	C45H89N2O6P	17.10	785.6521	90.18	264.2700, 184.0729
	93	SM (d18:1/22:2)	C45H87N2O6P	15.85	783.6374	98.59	264.2717, 184.0728
	94	SM (d18:1/22:3)	C45H85N2O6P	16.71	781.6218	94.77	264.2674, 184.0723
	95	SM (d18:1/23:0)	C46H93N2O6P	19.02	801.6844	99.32	264.2665, 184.0725
	96	SM (d18:1/23:2)	C46H89N2O6P	16.57	797.6506	92.95	264.2660, 184.0730
	97	SM (d18:1/23:3)	C46H87N2O6P	17.45	795.6354	81.68	264.2723, 184.0732
	98	SM (d18:1/24:0)	C47H95N2O6P	19.98	815.7001	97.84	264.2705, 184.0724
	99	SM (d18:1/24:1)	C47H93N2O6P	18.28	813.6844	98.88	264.2691, 184.0728
	100	SM (d18:1/24:4)	C47H87N2O6P	15.44	807.6375	97.83	264.2698, 184.0731
	101	SM (d18:1/25:0)	C48H97N2O6P	20.79	829.7155	99.43	264.2667, 184.0732
	102	SM (d18:1/25:1)	C48H95N2O6P	19.24	827.7001	95.78	264.2600, 184.0728
	103	SM (d18:1/25:3)	C48H91N2O6P	19.02	823.6665	96.01	264.2643, 184.0731
	104	SM (d18:1/25:4)	C48H89N2O6P	17.85	821.6506	88.64	264.2666, 184.0719
	105	SM (d18:1/26:1)	C49H97N2O6P	20.21	841.7157	99.25	264.2691, 184.0725
	106	SM (d18:1/26:2)	C49H95N2O6P	18.81	839.7001	99.73	264.2754, 184.0730
	107	SM (d18:1/26:3)	C49H93N2O6P	19.98	837.6800	95.27	264.2679, 184.0730
	108	SM (d18:1/26:4)	C49H91N2O6P	18.28	835.6683	94.37	264.2731, 184.0735
	109	SM (d18:2/18:1)	C41H79N2O6P	14.41	727.5749	98.92	262.2556, 184.0727
	110	SM (d18:2/18:3)	C41H75N2O6P	12.90	723.5436	90.63	262.2628, 184.0733
	111	SM (d18:2/20:0)	C43H85N2O6P	15.48	757.6211	92.69	262.2501, 184.0730
	112	SM (d18:2/20:2)	C43H81N2O6P	15.24	753.5905	91.53	262.2516, 184.0708
	113	SM (d18:2/20:3)	C43H79N2O6P	14.21	751.5749	86.38	262.2509, 184.0731
	114	SM (d18:2/21:0)	C44H87N2O6P	16.40	771.6353	98.99	262.2554, 184.0731
	115	SM (d18:2/23:0)	C46H91N2O6P	17.84	799.6679	98.81	781.6544, 262.2526, 184.0726
	116	SM (d18:2/24:0)	C47H93N2O6P	18.28	813.6842	98.28	262.2562, 184.0728
	117	SM (d18:2/24:2)	C47H89N2O6P	16.30	809.6533	98.67	262.2502, 184.0728
	118	SM (d20:0/24:4)	C49H93N2O6P	19.98	837.6844	87.77	294.3110, 184.0738
	119	SM (d20:1/23:4)	C48H89N2O6P	17.85	821.6531	93.97	292.3001, 184.0730
	120	SM (t18:0/16:1)	C39H79N2O7P	13.32	719.5698	96.54	300.2822, 264.2643, 184.0729
	[IS-8]	SM (d18:1/12:0)	C35H71N2O6P	9.94	647.5116	-	282.2776, 264.2681, 252.2660, 184.0731

^1^ IS, internal standard; So, sphingosine; Sa, sphinganine; S1P, sphingosine-1-phosphate; Cer, ceramide; C1P, ceramide-1-phosphate; HexCer, hexosylceramide; LacCer, lactosylceramide; SM, sphingomyelin. ^2^ Annotation of the sphingoid backbone denotes the number of hydroxyl groups, the number of carbon atoms, and the number of the unsaturation degree (e.g., d means two hydroxyl groups; t means three hydroxyl groups (i.e., phyto-)); annotation of the fatty acid chain indicates the number of carbon atoms and the number of unsaturation degree. ^3^ HexCer includes glucosylceramide (GlcCer) and galactosylceramide (GalCer), which cannot be distinguished by this method.

## Supplementary Materials

The following are available online, Figure S1. Characteristic MS/MS spectra of all identified SPLs by targeted-MS/MS analysis; Figure S2. Linear regression models constructed by plotting carbon number vs. retention time for SPLs; Figure S3. Intra- and inter-day RSDs of randomly selected SPLs in rat serum samples; Table S1. Linearity and sensitivity testing results of eight IS spiked in rat serum extracts; Table S2. Recovery testing results of eight IS spiked in rat serum extracts.
