# Supplementary material for: Sphingolipidomic Profiling of Rat Serum by UPLC-Q-TOF-MS: Application to Rheumatoid Arthritis Study
Source: Molecules. 2018 May 31;23(6):1324. doi: 10.3390/molecules23061324 (PMC6099492; doi:10.3390/molecules23061324)
Supplement: Supplementary file 1 [file molecules-23-01324-s001.pdf]

Supplementary materials

Sphingolipidomic profiling of rat serum by UPLC-Q-TOF-MS: Application to rheumatoid arthritis study

Fanghui Qu [1], Hongyang Zhang [1,2,*], Min Zhang [2], and Ping Hu [1,*]

[1] School of Chemistry and Molecular Engineering, East China University of Science and Technology, Shanghai 200237, China; 13162300607@163.com (F.Q.)

[2] Shanghai Key Laboratory of New Drug Design, School of Pharmacy, East China University of Science and Technology, Shanghai 200237, China; zhangm@ecust.edu.cn (M.Z.)

[*] Correspondence: hongyang_zhang@ecust.edu.cn (H.Z.); huping@ecust.edu.cn (P.H.); Tel.: +86-021-6425-2844

Contents

Figure S1. Characteristic MS/MS spectra of all identified SPLs by targeted-MS/MS analysis

Figure S2. Linear regression models constructed by plotting carbon number *vs.* retention time for SPLs

Figure S3. Intra- and inter-day RSDs of randomly selected SPLs in rat serum sample

Table S1. Linearity and sensitivity testing results of 8 IS spiked in rat serum extracts

Table S2. Recovery testing results of 8 IS spiked in rat serum extracts

Figure S1. Characteristic MS/MS spectra of all identified SPLs by targeted-MS/MS analysis

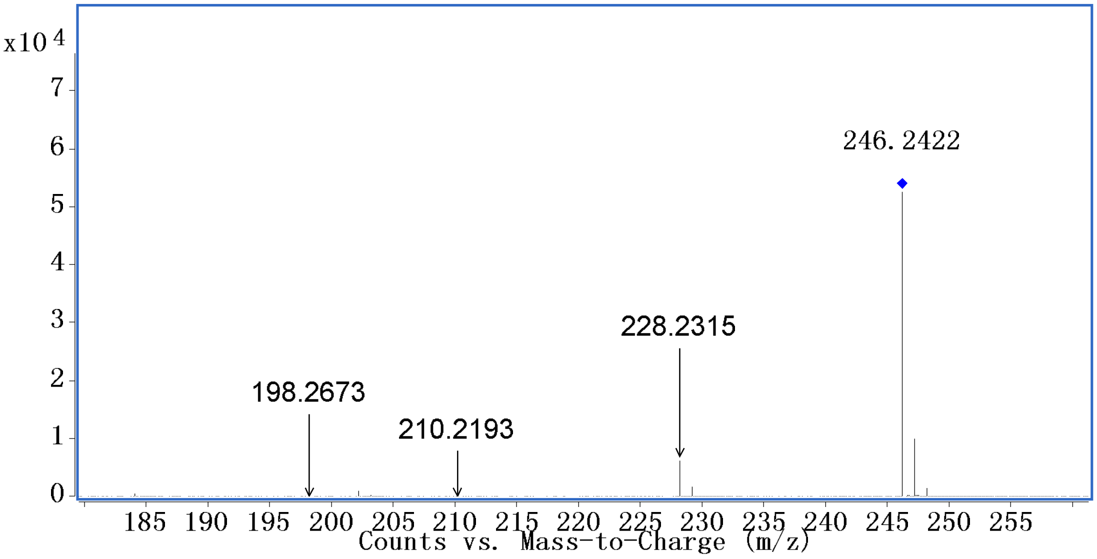

No.1 **Sa (d14:0)**: $[M+H]^+$ 246.2422, $[M-H_2O+H]^+$ 228.2315, $[M-2H_2O+H]^+$ 210.2193, $[M-H_2O-HCHO+H]^+$ 198.2673, the collision energy was set as 20eV.

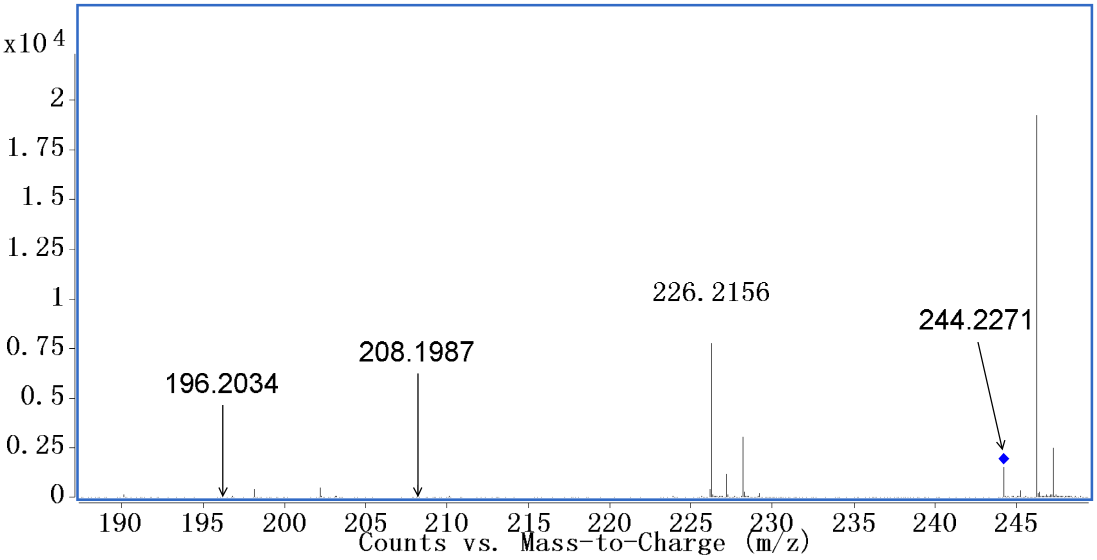

No.2 **So (d14:1)**: $[M+H]^+$ 244.2271, $[M-H_2O+H]^+$ 226.2156, $[M-2H_2O+H]^+$ 208.1987, $[M-H_2O-HCHO+H]^+$ 196.2034, the collision energy was set as 20eV.

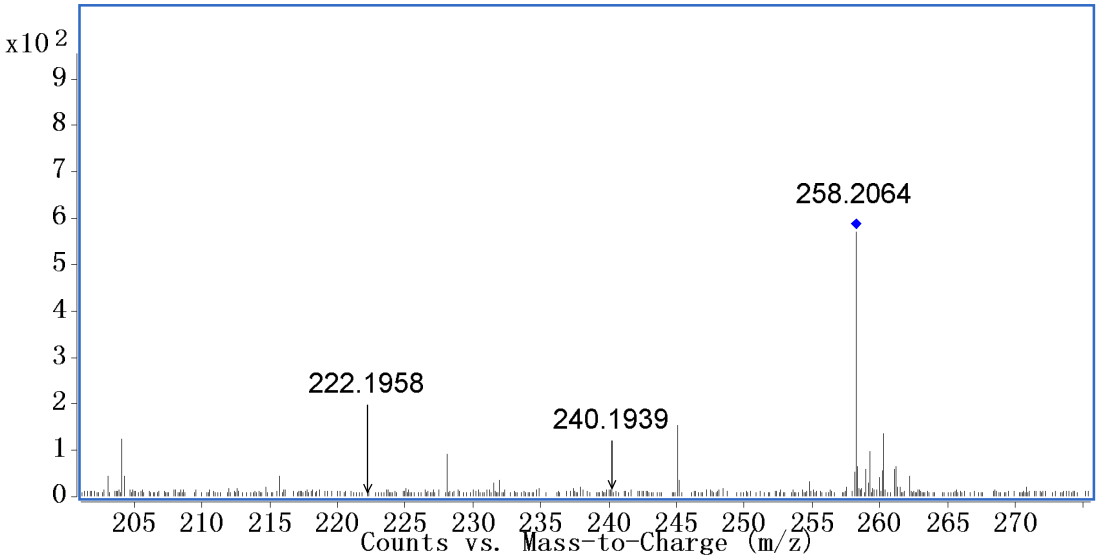

No.3 **So (t14:2)**: $[M+H]^+$ 258.2064, $[M-H_2O+H]^+$ 240.1939, $[M-2H_2O+H]^+$ 222.1958, the collision energy was set as 20eV.

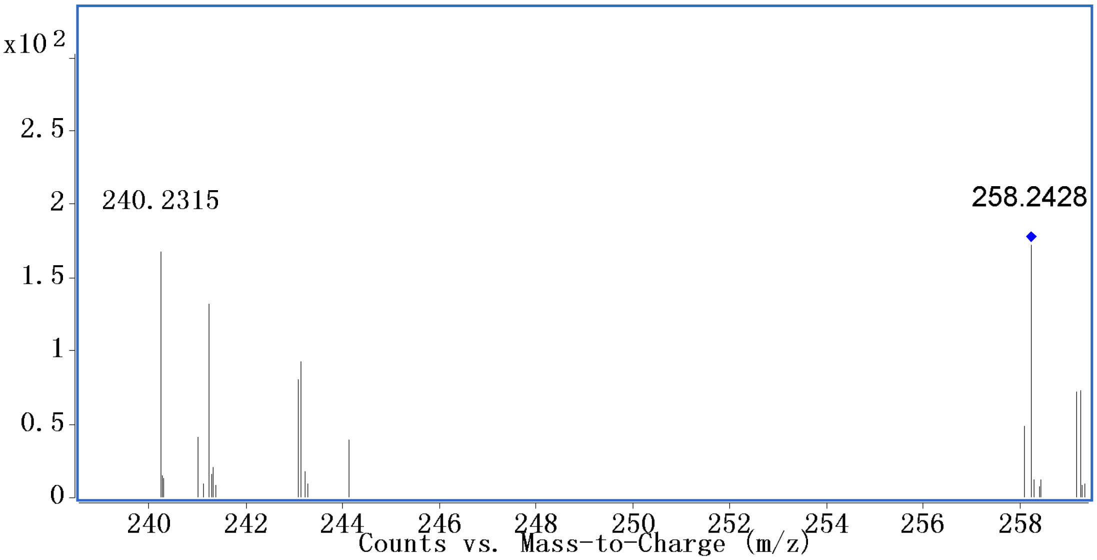

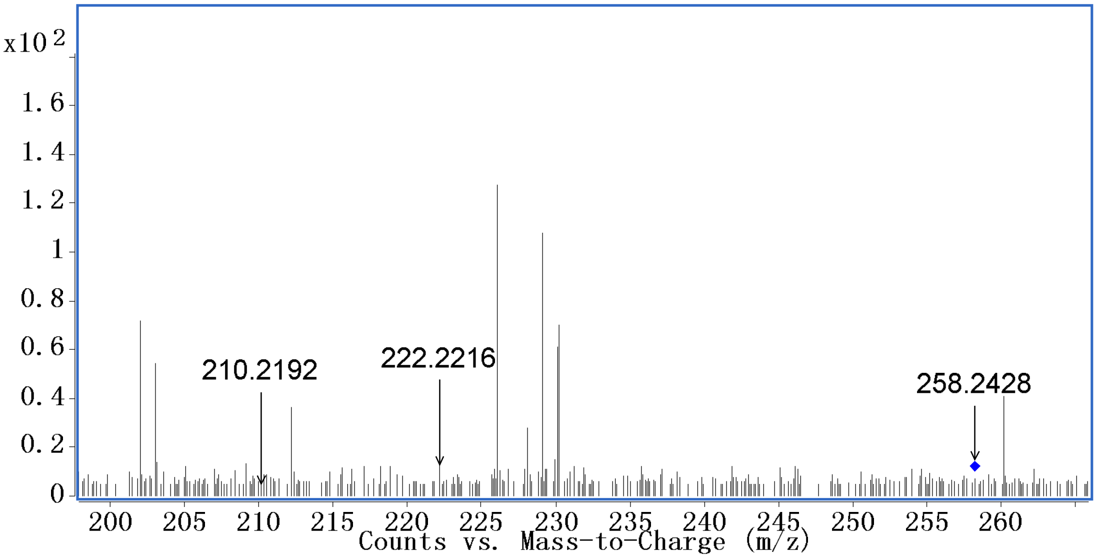

No.4 **So (d15:1)**: $[M+H]^+$ 258.2428, $[M-H_2O+H]^+$ 240.2315, the collision energy was set as 10eV; $[M-2H_2O+H]^+$ 222.2216, $[M-H_2O-HCHO+H]^+$ 210.2192, the collision energy was set as 40eV.

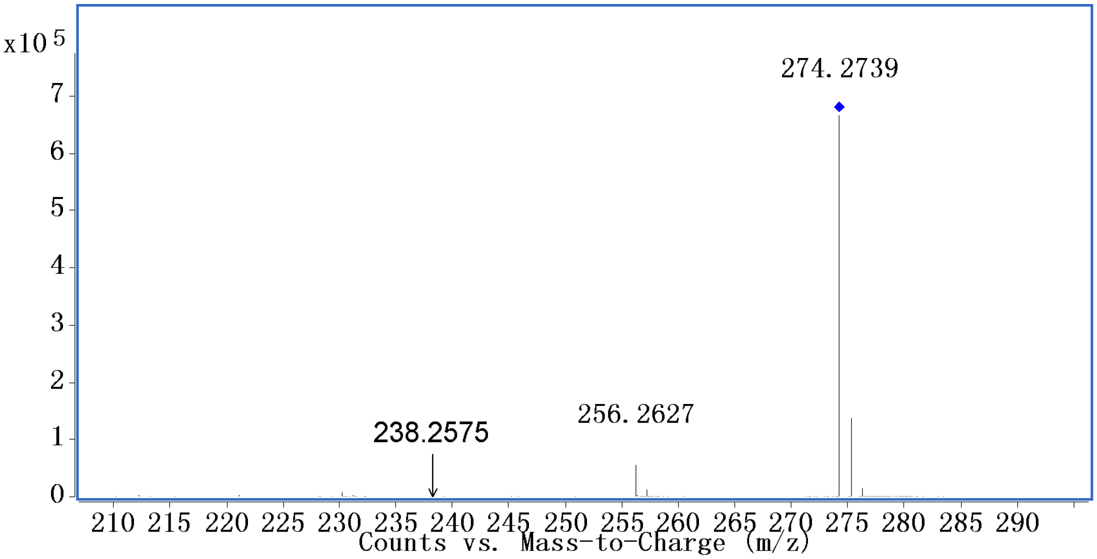

No.5 **Sa (d16:0)**: $[M+H]^+$ 274.2739, $[M-H_2O+H]^+$ 256.2627, $[M-2H_2O+H]^+$ 238.2575, the collision energy was set as 20eV.

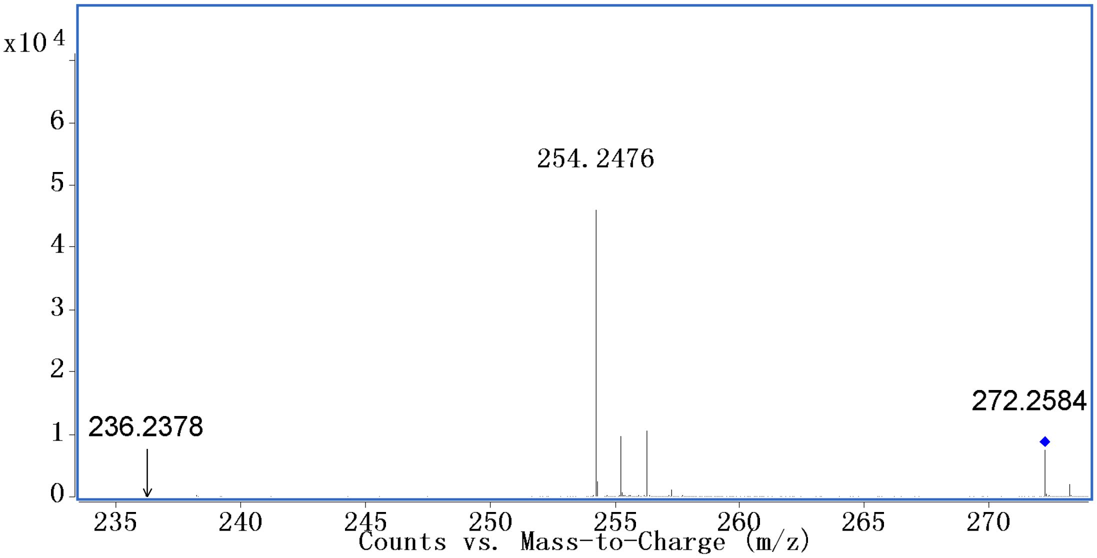

No.6 **So (d16:1)**: $[M+H]^+$ 272.2584, $[M-H_2O+H]^+$ 254.2476, $[M-2H_2O+H]^+$ 236.2378, the collision energy was set as 20eV.

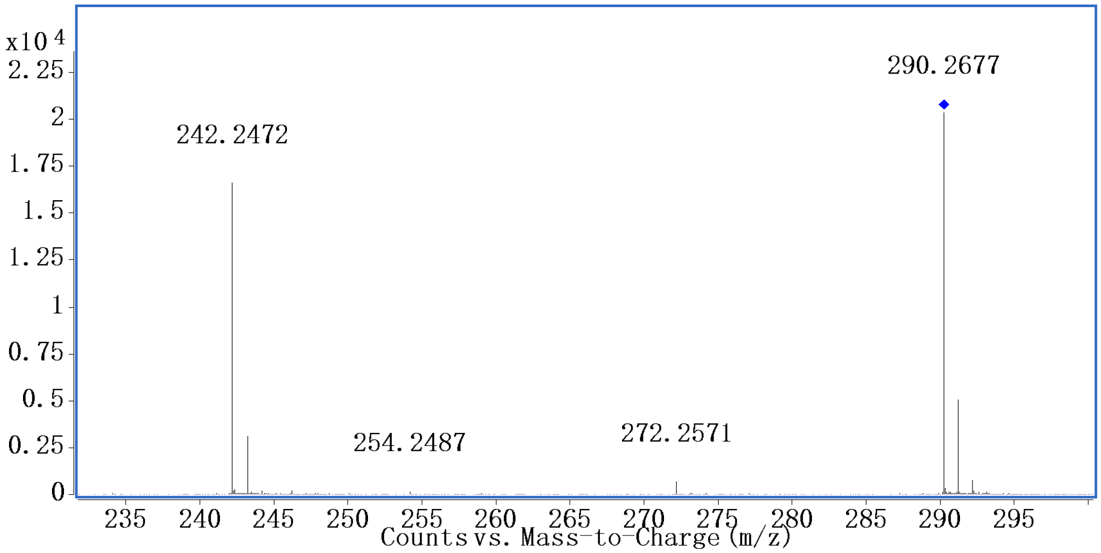

No.7 **Sa (t16:0)**: $[M+H]^+$ 290.2677, $[M-H_2O+H]^+$ 272.2571, $[M-2H_2O+H]^+$ 254.2487, $[M-H_2O-HCHO+H]^+$ 242.2472, the collision energy was set as 20eV.

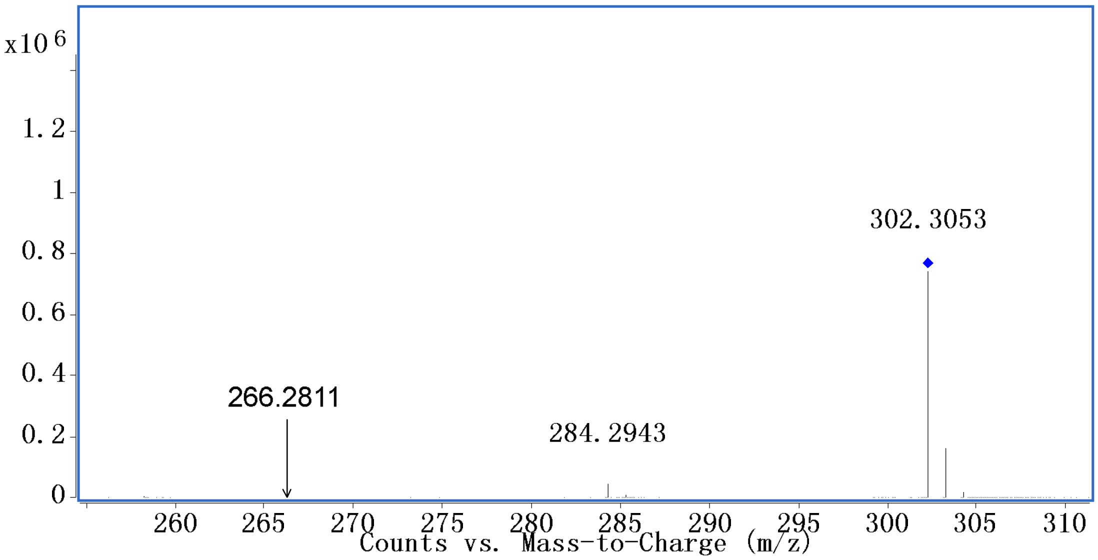

No.8 **Sa (d18:0)**: $[M+H]^+$ 302.3053, $[M-H_2O+H]^+$ 284.2943, $[M-2H_2O+H]^+$ 266.2811, the collision energy was set as 20eV.

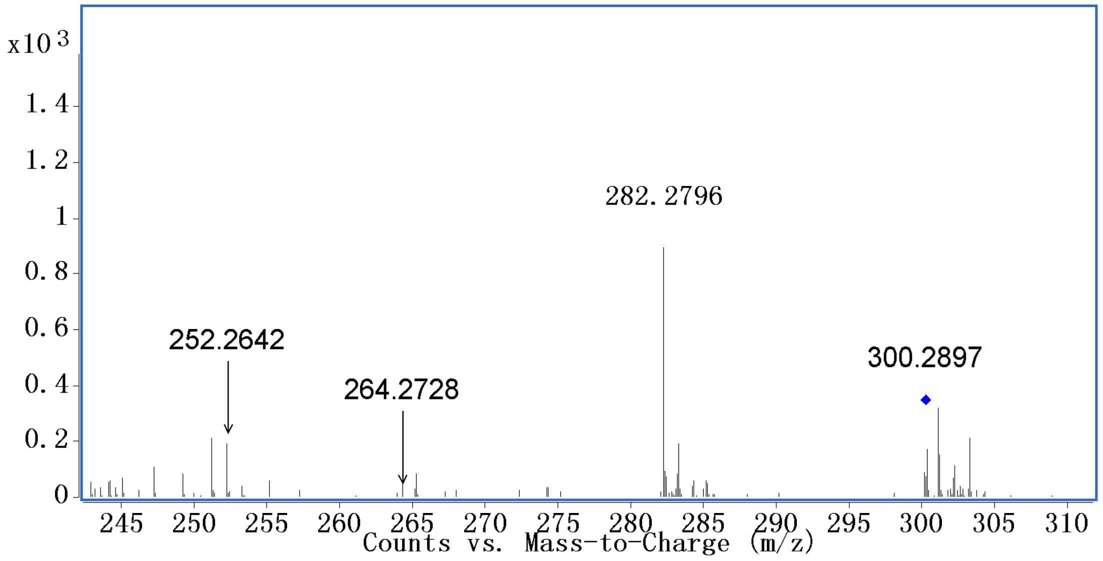

No.9 **So (d18:1)**: $[M+H]^+$ 300.2897, $[M-H_2O+H]^+$ 282.2796, $[M-2H_2O+H]^+$ 264.2728, $[M-H_2O-HCHO+H]^+$ 252.2642, the collision energy was set as 20eV.

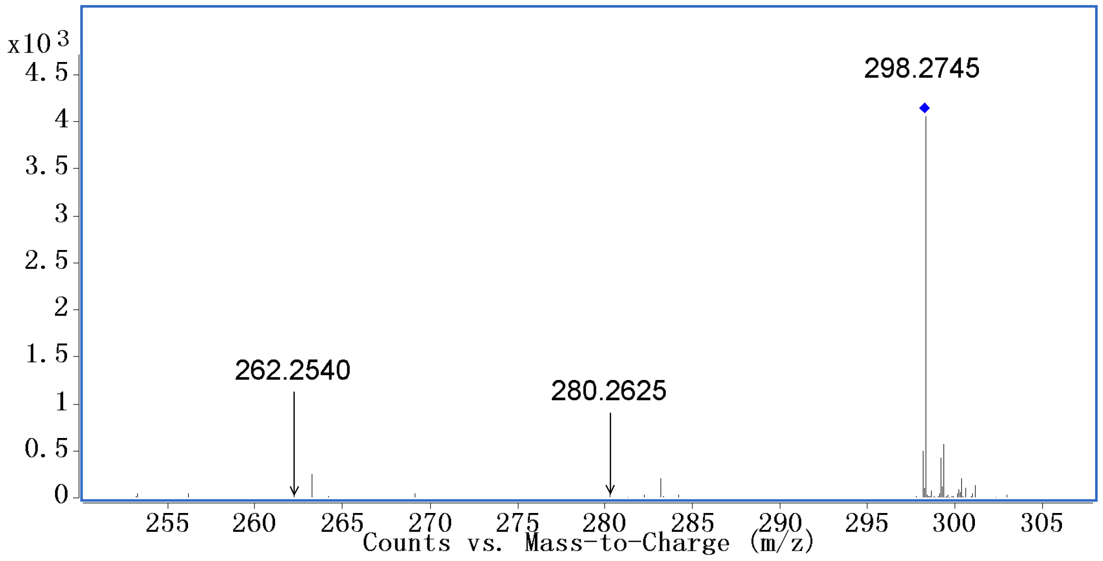

No.10 **So (d18:2)**: $[M+H]^+$ 298.2745, $[M-H_2O+H]^+$ 280.2625, $[M-2H_2O+H]^+$ 262.2540, the collision energy was set as 20eV.

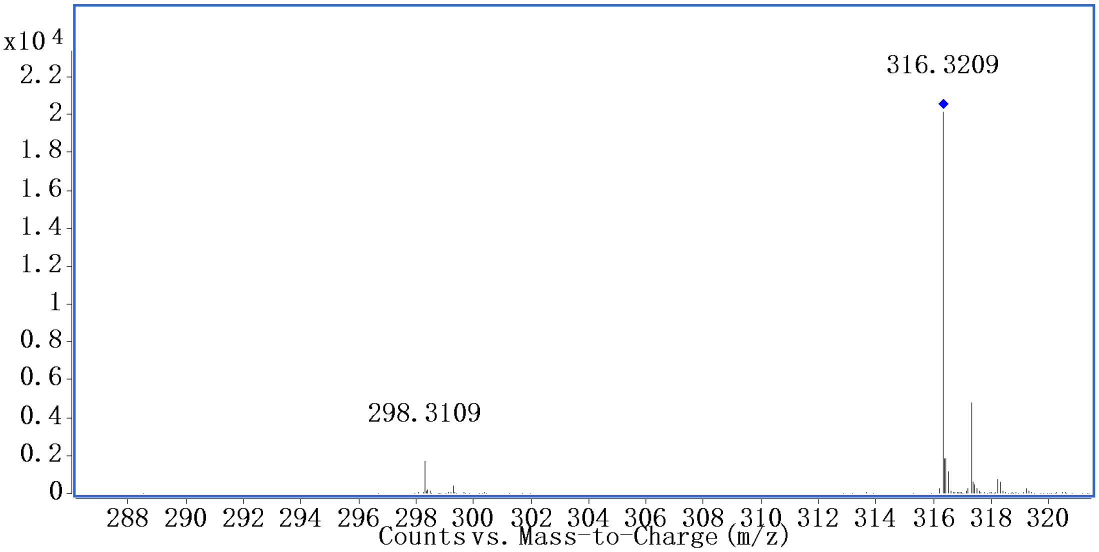

No.11 **Sa (d19:0)**: $[M+H]^+$ 316.3209, $[M\text{-}H_2O+H]^+$ 298.3109, the collision energy was set as 20eV.

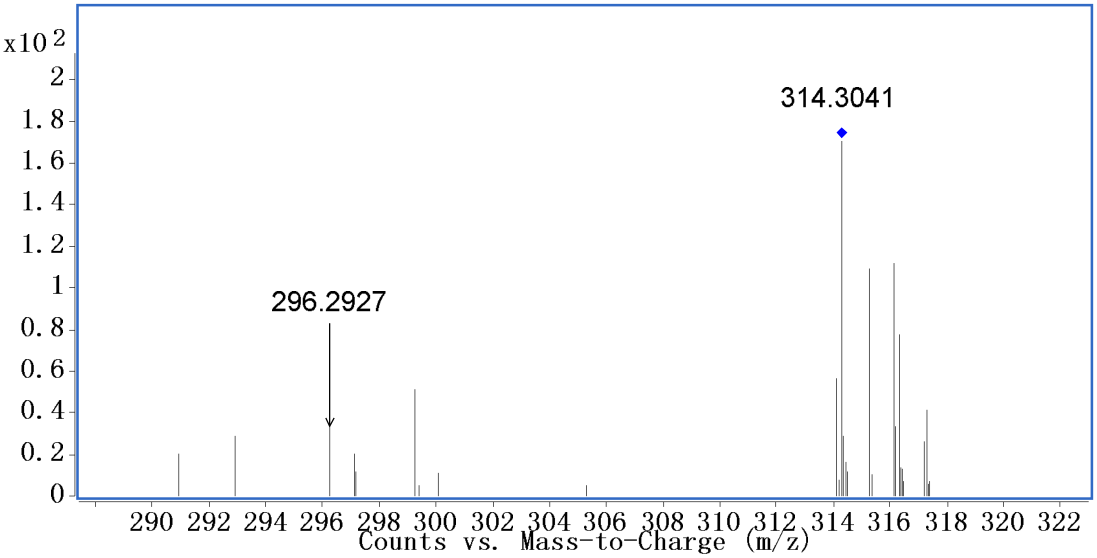

No.12 **So (d19:1)**: $[M+H]^+$ 314.3041, $[M\text{-}H_2O+H]^+$ 296.2927, the collision energy was set as 20eV.

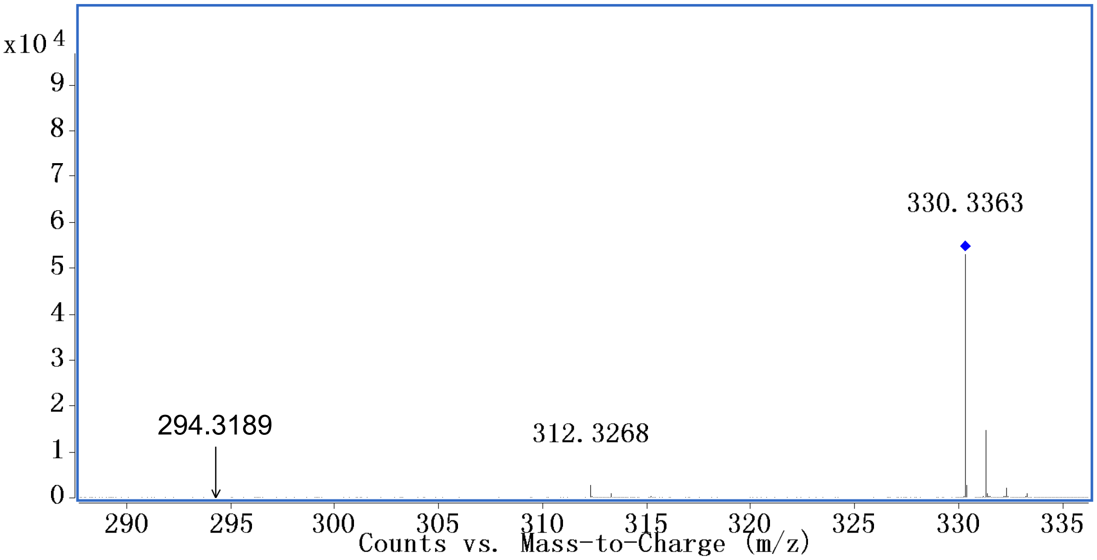

No.13 **Sa (d20:0)**: $[M+H]^+$ 330.3363, $[M\text{-}H_2O+H]^+$ 312.3268, $[M\text{-}2H_2O+H]^+$ 294.3189, the collision energy was set as 20eV.

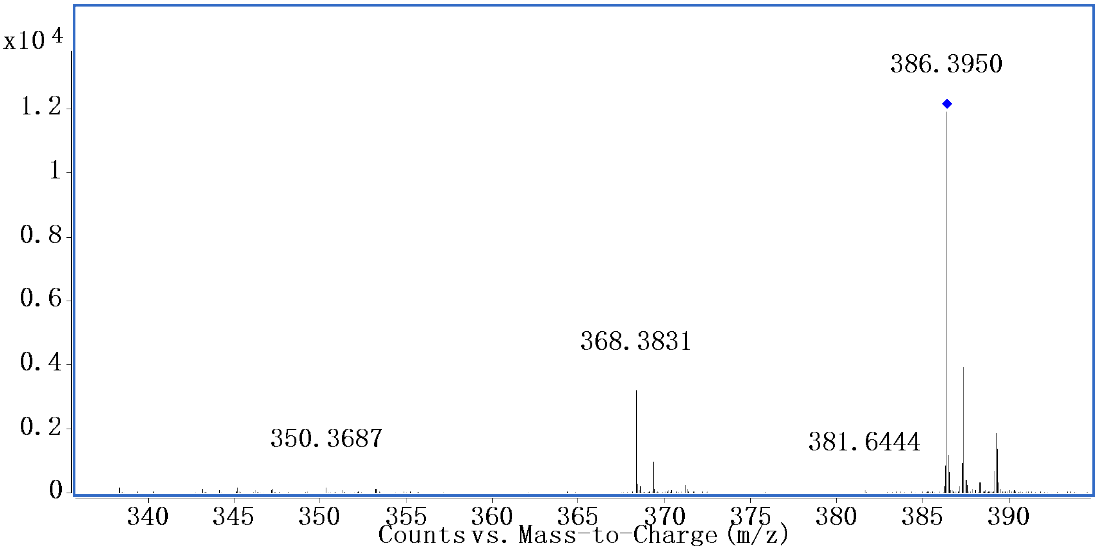

No.14 **Sa (d24:0)**: $[M+H]^+$ 386.3950, $[M\text{-}H_2O+H]^+$ 368.3831, $[M\text{-}2H_2O+H]^+$ 350.3687, the collision energy was set as 20eV.

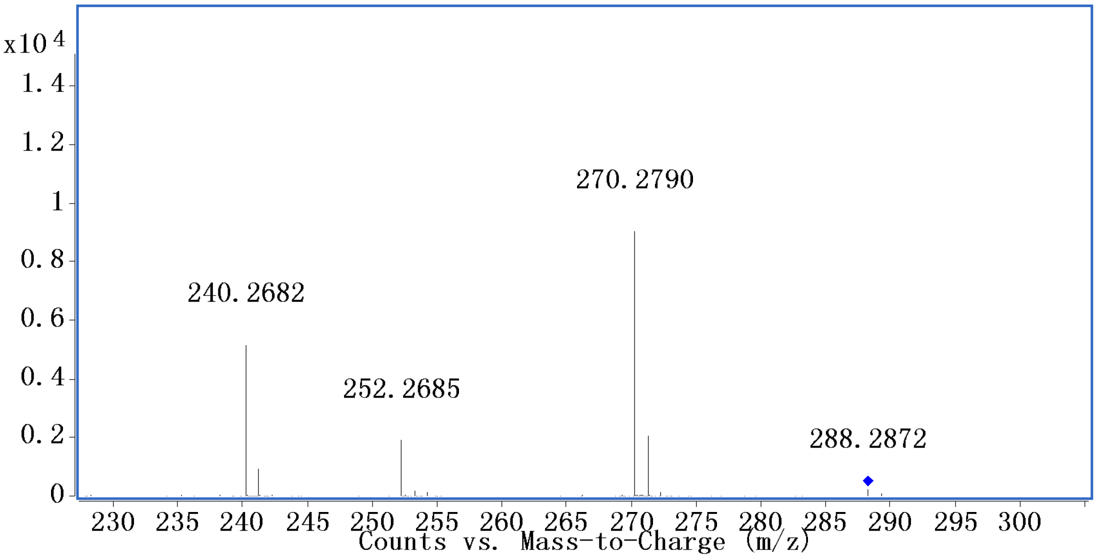

[IS-1] **Sa (d17:0)**: $[M+H]^+$ 288.2872, $[M\text{-}H_2O+H]^+$ 270.2790, $[M\text{-}2H_2O+H]^+$ 252.2685, $[M\text{-}H_2O\text{-}HCHO+H]^+$ 240.2682, the collision energy was set as 20eV.

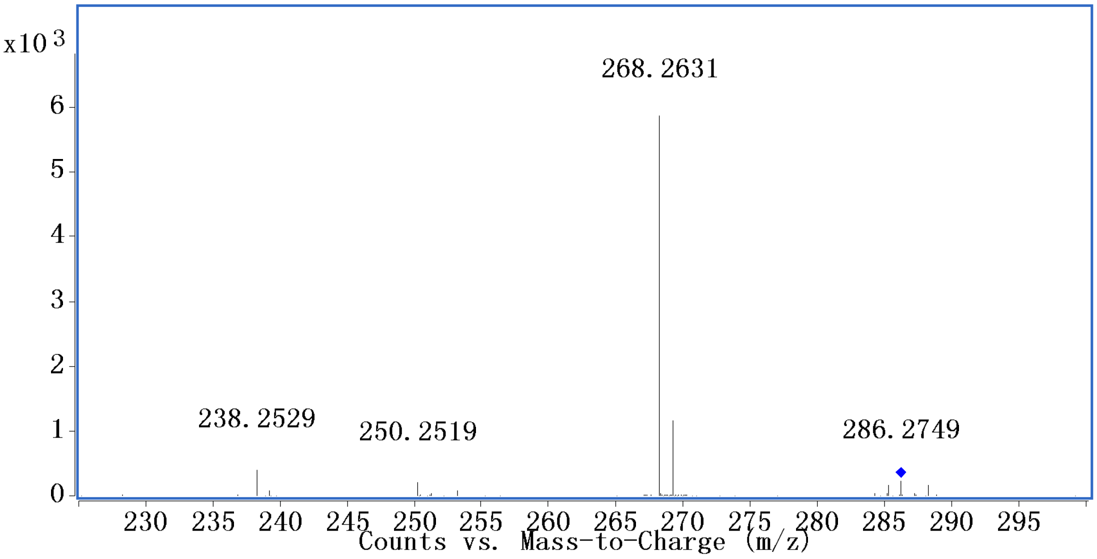

[IS-2] **So (d17:1)**: $[M+H]^+$ 286.2749, $[M\text{-}H_2O+H]^+$ 268.2631, $[M\text{-}2H_2O+H]^+$ 250.2519, $[M\text{-}H_2O\text{-}HCHO+H]^+$ 238.2529, the collision energy was set as 20eV.

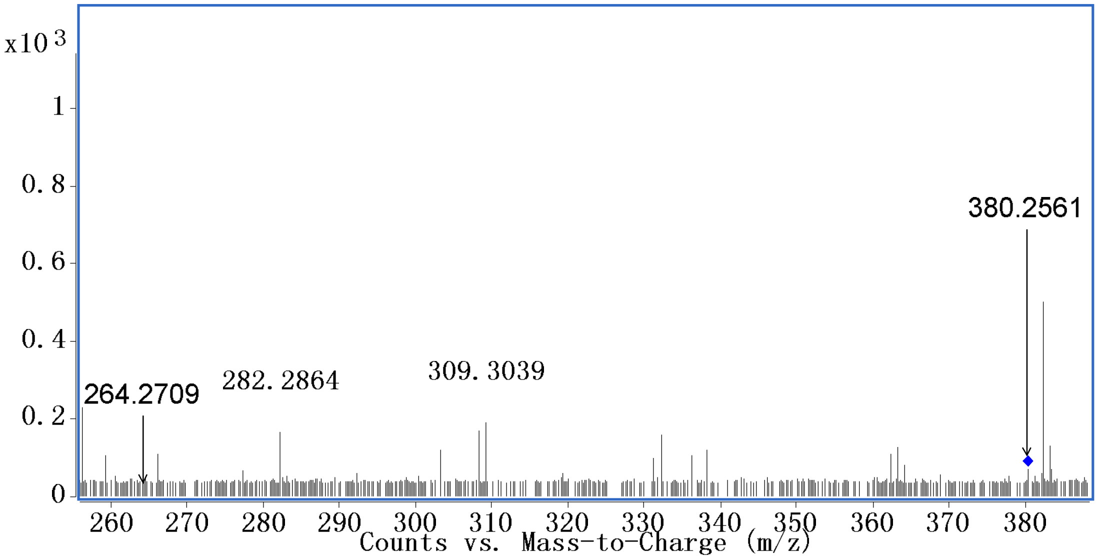

No.15 **S1P (d18:1)**: $[M+H]^+$ 380.2561, [So (d18:1)-H_2O+H]$^+$ 282.2864, [So (d18:1)-2H_2O+H]$^+$ 264.2709, the collision energy was set as 20eV.

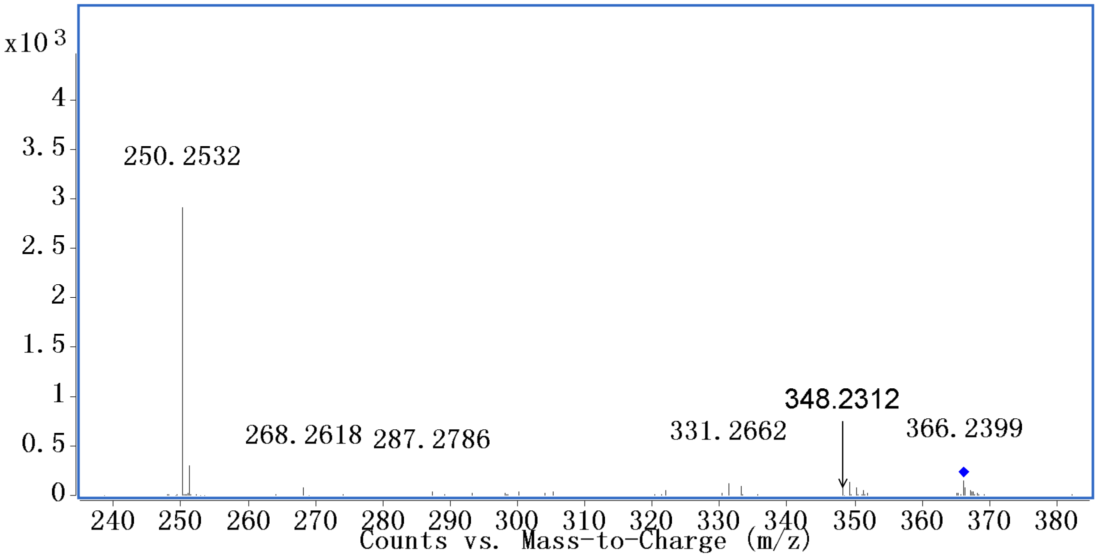

[IS-3] **S1P (d17:1)**: $[M+H]^+$ 366.2399, [M-H_2O+H]$^+$ 348.2312, [So (d17:1)-H_2O+H]$^+$ 268.2618, [So (d17:1)-2H_2O+H]$^+$ 250.2532, the collision energy was set as 20eV.

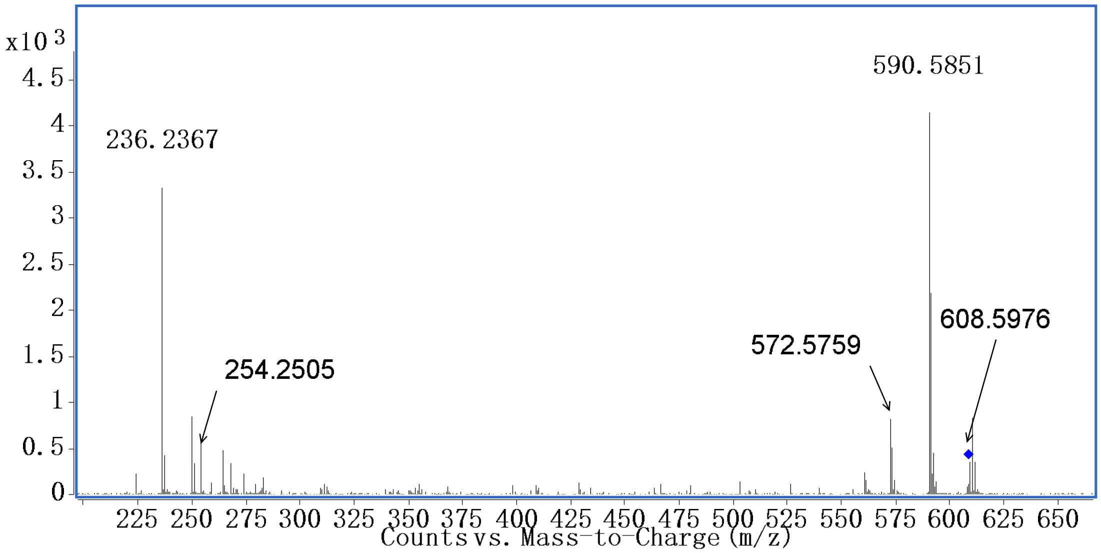

No.16 **Cer (d16:1/23:0)**: $[M+H]^+$ 608.5976, $[M-H_2O+H]^+$ 590.5851, $[M-2H_2O+H]^+$ 572.5759, [So (d16:1)-H_2O+H]$^+$ 254.2505, [So (d16:1)-$2H_2O$+H]$^+$ 236.2367, the collision energy was set as 20eV.

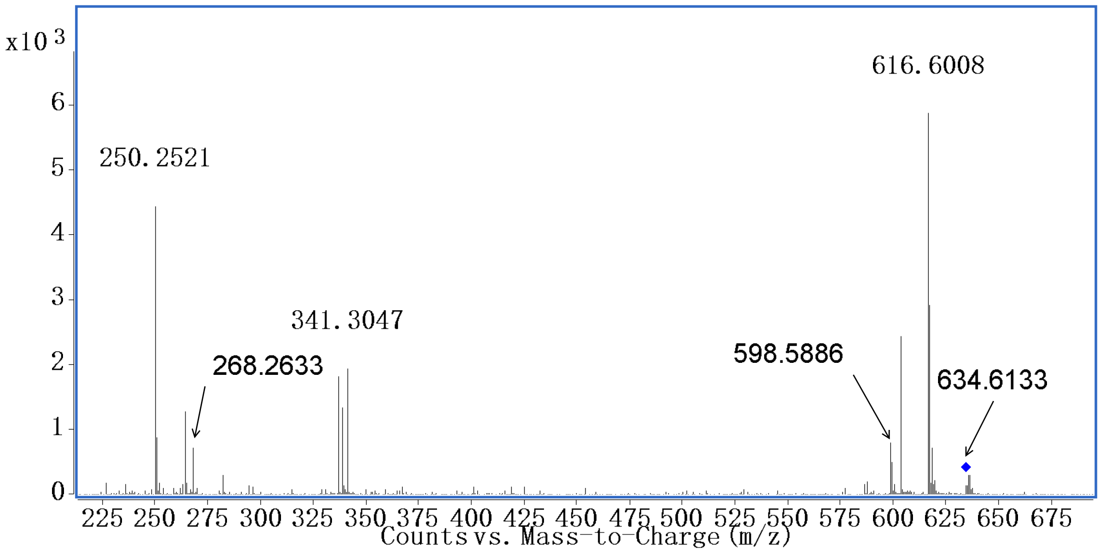

No.17 **Cer (d17:1/24:1)**: $[M+H]^+$ 634.6133, $[M-H_2O+H]^+$ 616.6008, $[M-2H_2O+H]^+$ 598.5886, [So (d17:1)-H_2O+H]$^+$ 268.2633, [So (d17:1)-$2H_2O$+H]$^+$ 250.2521, the collision energy was set as 40eV.

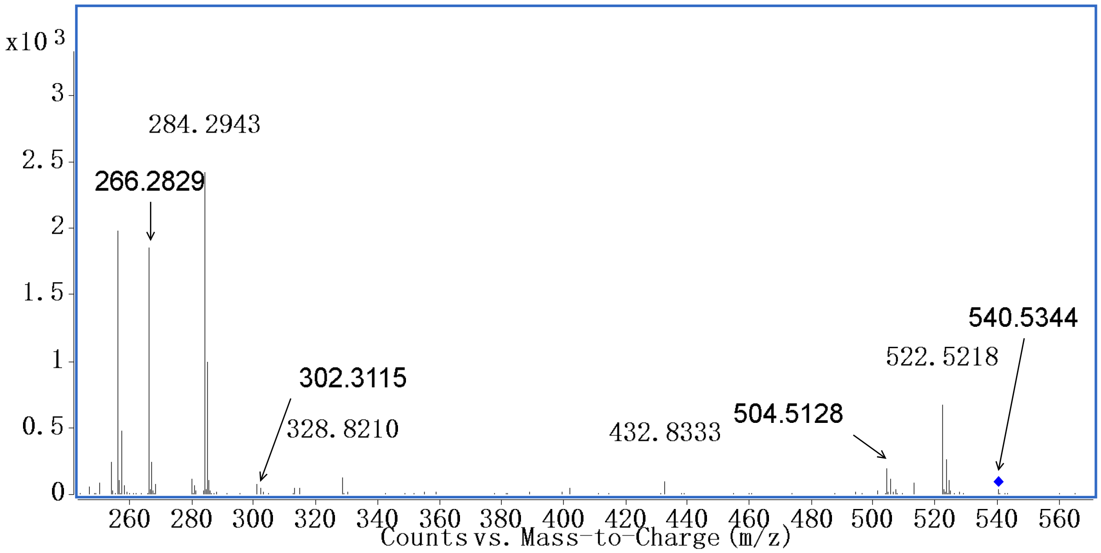

No.18 **Cer (d18:0/16:0)**: $[M+H]^+$ 540.5344, $[M-H_2O+H]^+$ 522.5218, $[M-2H_2O+H]^+$ 504.5128, [Sa (d18:0)+H]$^+$ 302.3115, [Sa (d18:0)-H_2O+H]$^+$ 284.2943, [Sa (d18:0)-$2H_2O$+H]$^+$ 266.2829, the collision energy was set as 40eV.

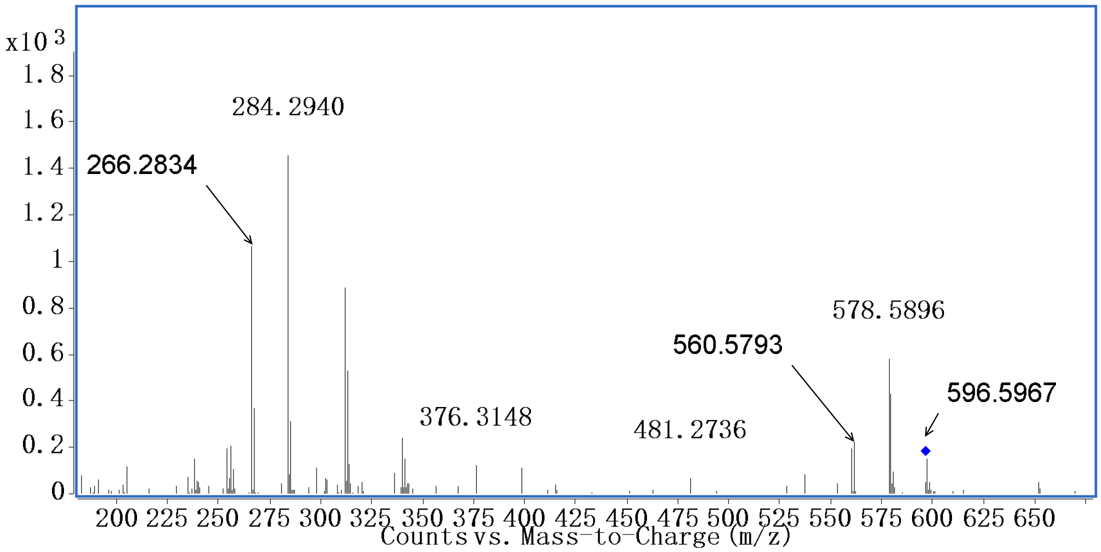

No.19 **Cer (d18:0/20:0)**: $[M+H]^+$ 596.5967, $[M-H_2O+H]^+$ 578.5896, $[M-2H_2O+H]^+$ 560.5793, [Sa (d18:0)-H_2O+H]$^+$ 284.2940, [Sa (d18:0)-$2H_2O$+H]$^+$ 266.2834, the collision energy was set as 40eV.

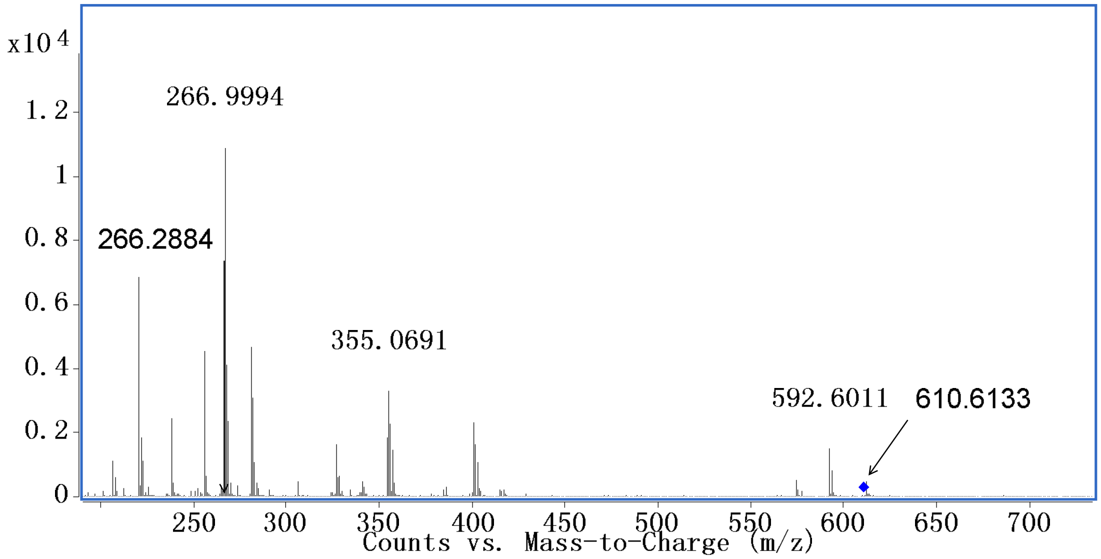

No.20 **Cer (d18:0/21:0)**: $[M+H]^+$ 610.6133, $[M-H_2O+H]^+$ 592.6011, [Sa (d18:0)-$2H_2O+H]^+$ 266.2884, the collision energy was set as 40eV.

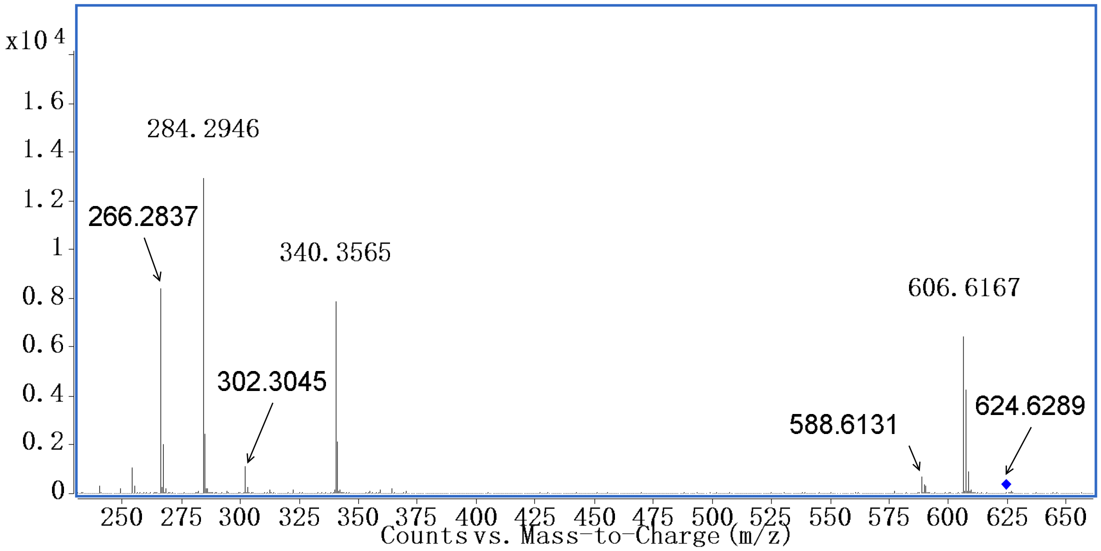

No.21 **Cer (d18:0/22:0)**: $[M+H]^+$ 624.6289, $[M-H_2O+H]^+$ 606.6167, $[M-2H_2O+H]^+$ 588.6131, [Sa (d18:0)$+H]^+$ 302.3045, [Sa (d18:0)-$H_2O+H]^+$ 284.2946, [Sa (d18:0)-$2H_2O+H]^+$ 266.2837, the collision energy was set as 40eV.

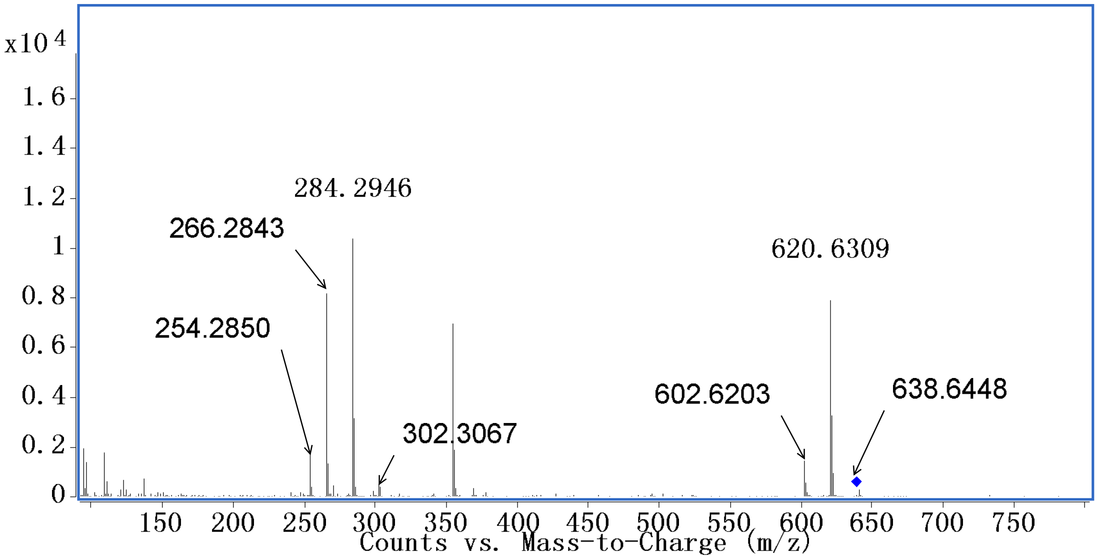

No.22 **Cer (d18:0/23:0)**: $[M+H]^+$ 638.6448, $[M-H_2O+H]^+$ 620.6309, $[M-2H_2O+H]^+$ 602.6203, [Sa (d18:0)+H]$^+$ 302.3067, [Sa (d18:0)-H_2O+H]$^+$ 284.2946, [Sa (d18:0)-$2H_2O$+H]$^+$ 266.2843, [Sa (d18:0)-H_2O-HCHO+H]$^+$ 254.2850, the collision energy was set as 40eV.

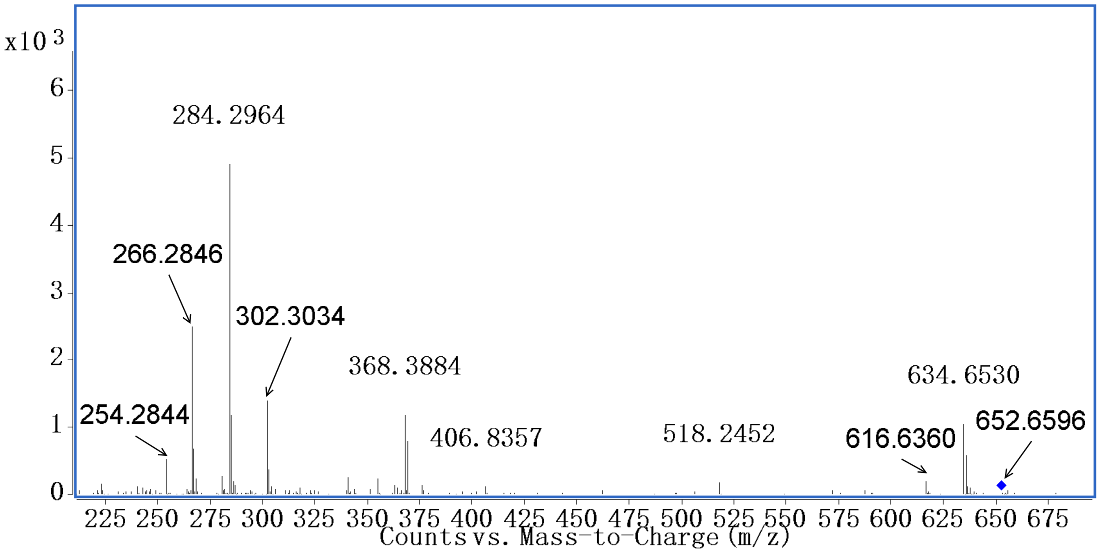

No.23 **Cer (d18:0/24:0)**: $[M+H]^+$ 652.6596, $[M-H_2O+H]^+$ 634.6530, $[M-2H_2O+H]^+$ 616.6360, [Sa (d18:0)+H]$^+$ 302.3034, [Sa (d18:0)-H_2O+H]$^+$ 284.2964, [Sa (d18:0)-$2H_2O$+H]$^+$ 266.2846, [Sa (d18:0)-H_2O-HCHO+H]$^+$ 254.2844, the collision energy was set as 40eV.

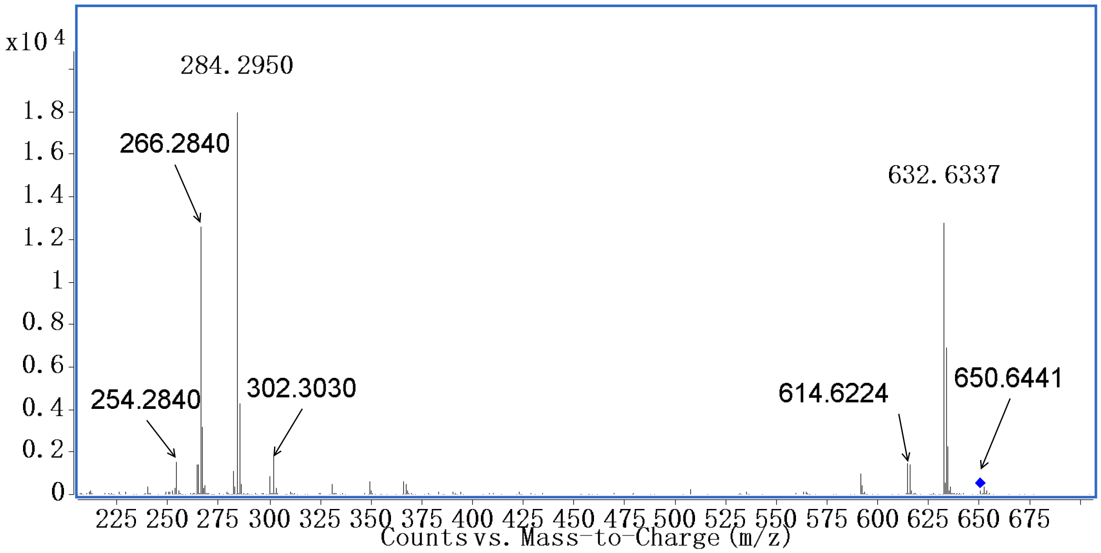

No.24 **Cer (d18:0/24:1)**: $[M+H]^+$ 650.6441, $[M-H_2O+H]^+$ 632.6337, $[M-2H_2O+H]^+$ 614.6224, [Sa (d18:0)+H]$^+$ 302.3030, [Sa (d18:0)-H_2O+H]$^+$ 284.2950, [Sa (d18:0)-$2H_2O$+H]$^+$ 266.2840, [Sa (d18:0)-H_2O-HCHO+H]$^+$ 254.2840, the collision energy was set as 40eV.

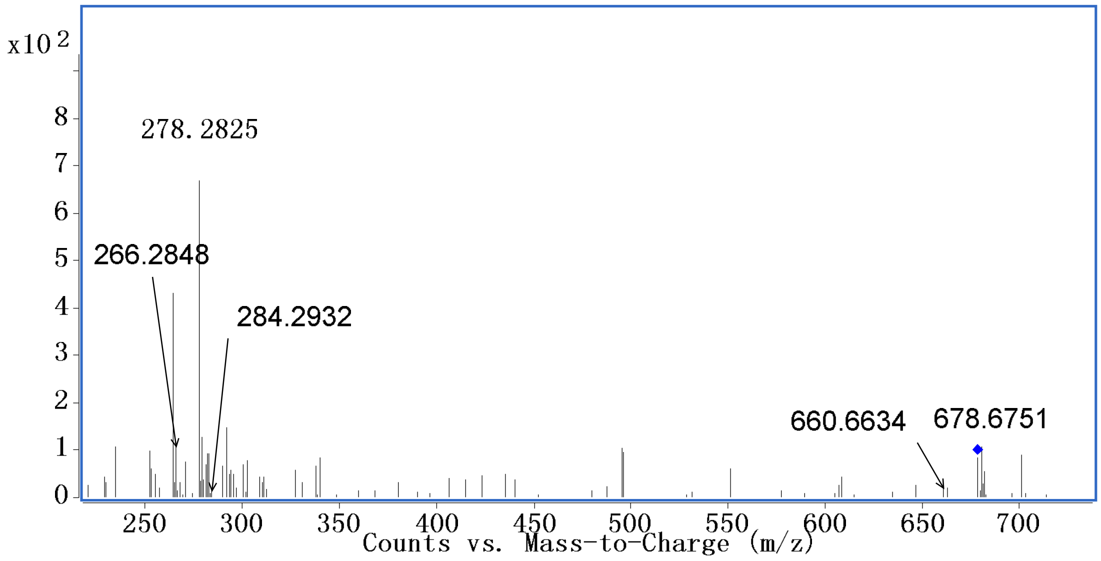

No.25 **Cer (d18:0/26:1)**: $[M+H]^+$ 678.6751, $[M-H_2O+H]^+$ 660.6634, [Sa (d18:0)-H_2O+H]$^+$ 284.2932, [Sa (d18:0)-$2H_2O$+H]$^+$ 266.2848, the collision energy was set as 40eV.

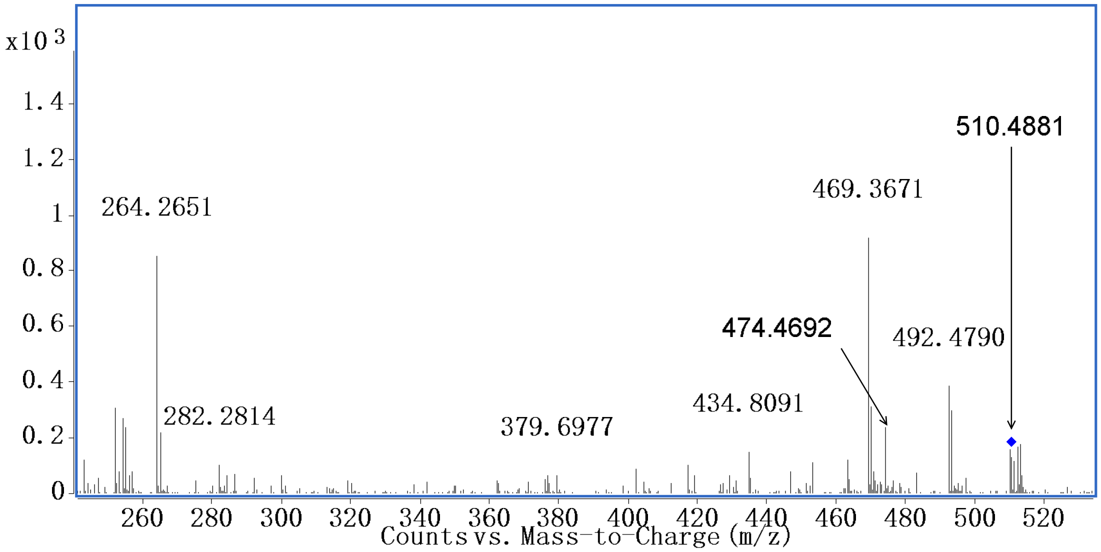

No.26 **Cer (d18:1/14:0)**: $[M+H]^+$ 510.4881, $[M\text{-}H_2O+H]^+$ 492.4790, $[M\text{-}2H_2O+H]^+$ 474.4692, [So (d18:1)-$H_2O+H]^+$ 282.2814, [So (d18:1)-$2H_2O+H]^+$ 264.2651, the collision energy was set as 40eV.

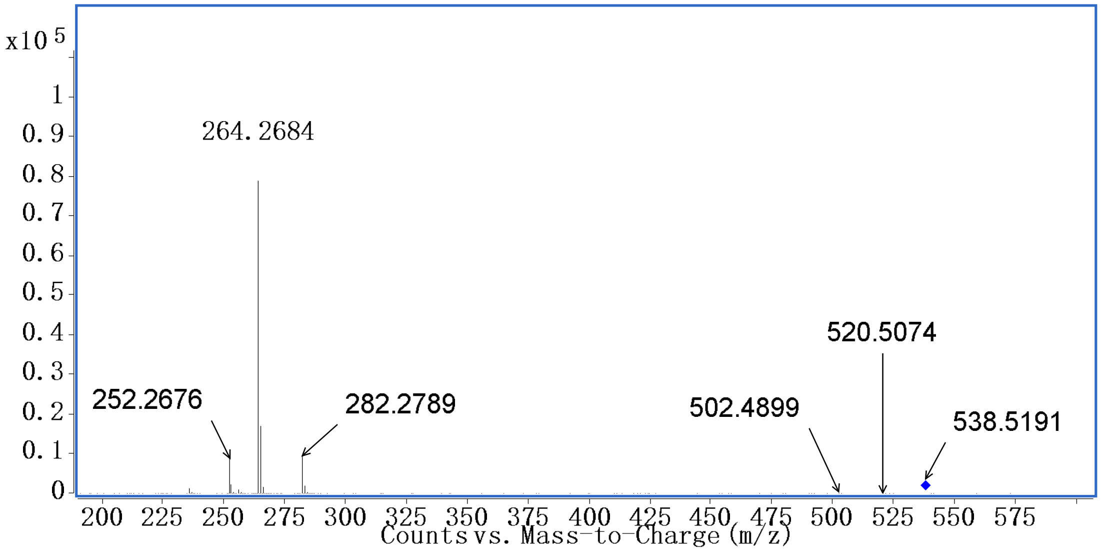

No.27 **Cer (d18:1/16:0)**: $[M+H]^+$ 538.5191, $[M\text{-}H_2O+H]^+$ 520.5074, $[M\text{-}2H_2O+H]^+$ 502.4899, [So (d18:1)-$H_2O+H]^+$ 282.2789, [So (d18:1)-$2H_2O+H]^+$ 264.2684, [So (d18:1)-H_2O-$HCHO+H]^+$ 252.2676, the collision energy was set as 40eV.

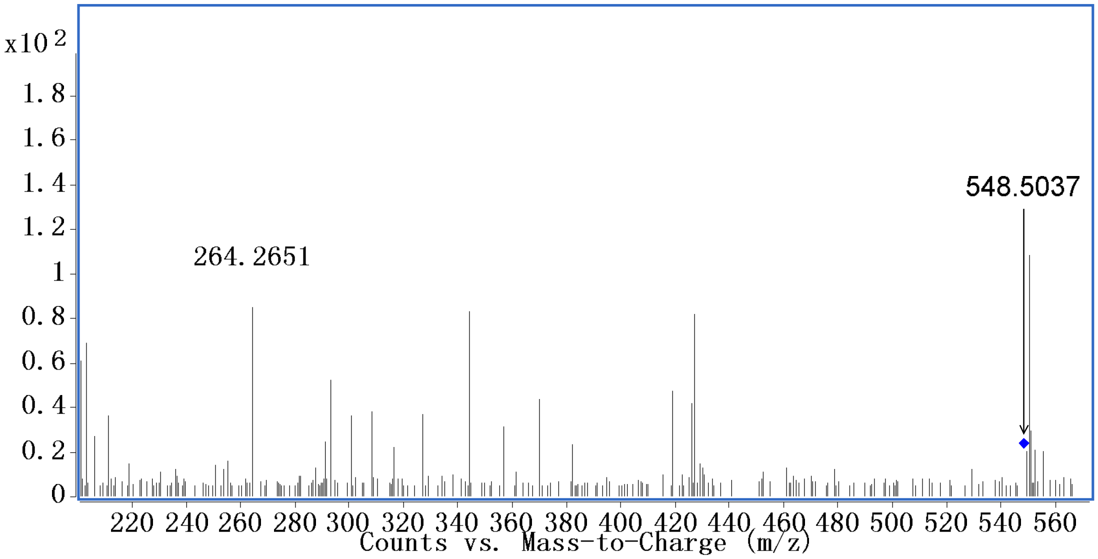

No.28 **Cer (d18:1/17:2)**: $[M+H]^+$ 548.5037, [So (d18:1)-$2H_2O$+H]$^+$ 264.2651, the collision energy was set as 40eV.

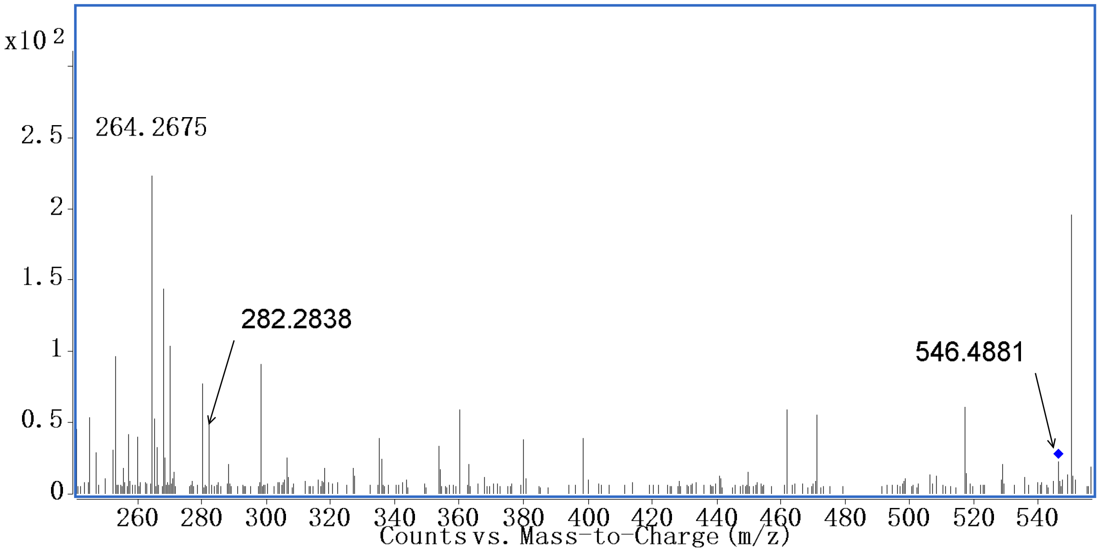

No.29 **Cer (d18:1/17:3)**: $[M+H]^+$ 546.4881, [So (d18:1)-H_2O+H]$^+$ 282.2838, [So (d18:1)-$2H_2O$+H]$^+$ 264.2675, the collision energy was set as 40eV.

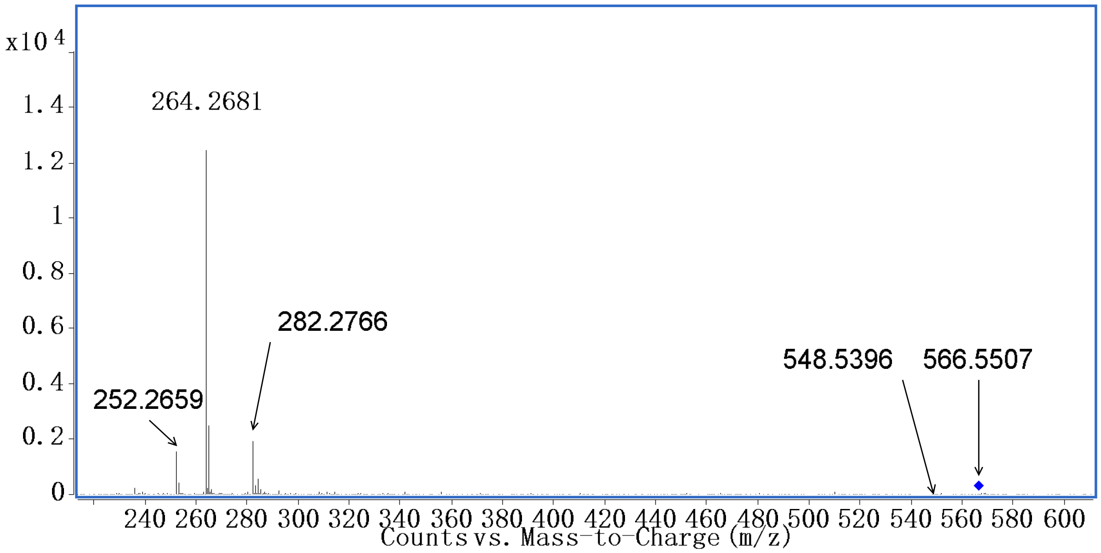

No.30 **Cer (d18:1/18:0)**: $[M+H]^+$ 566.5507, $[M-H_2O+H]^+$ 548.5396, [So (d18:1)-$H_2O+H]^+$ 282.2766, [So (d18:1)-$2H_2O+H]^+$ 264.2681, [So (d18:1)-H_2O-HCHO+H$]^+$ 252.2659, the collision energy was set as 40eV.

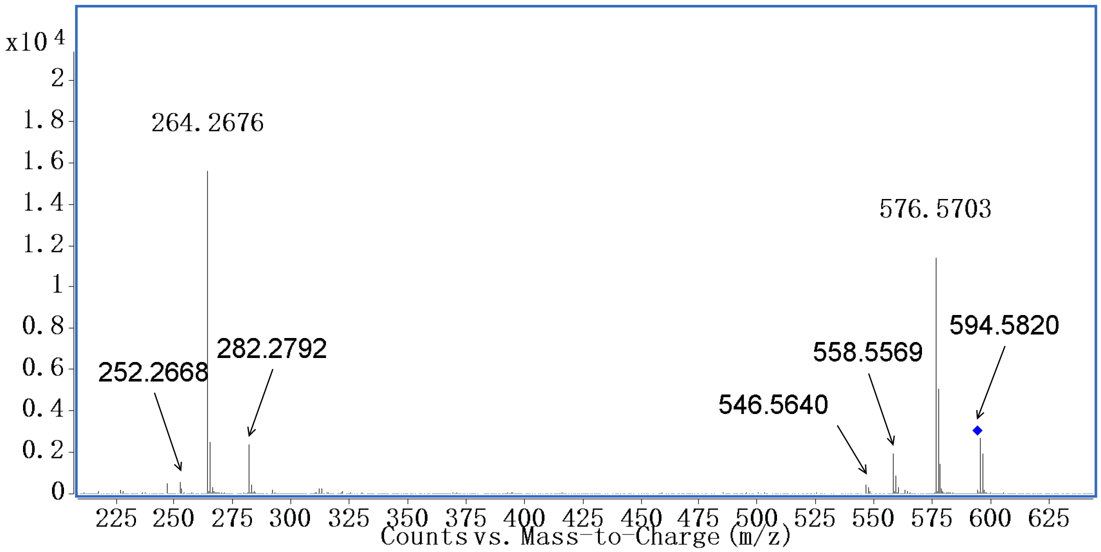

No.31 **Cer (d18:1/20:0)**: $[M+H]^+$ 594.5820, $[M-H_2O+H]^+$ 576.5703, $[M-2H_2O+H]^+$ 558.5569, $[M-H_2O$-HCHO+H$]^+$ 546.5640, [So (d18:1)-$H_2O+H]^+$ 282.2792, [So (d18:1)-$2H_2O+H]^+$ 264.2676, [So (d18:1)-H_2O-HCHO+H$]^+$ 252.2668, the collision energy was set as 20eV.

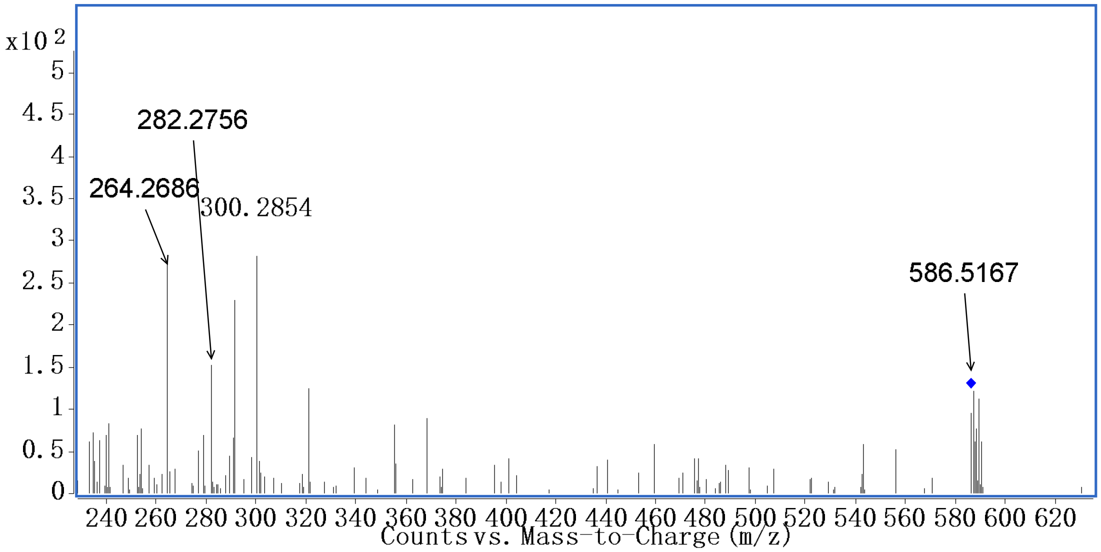

No.32 **Cer (d18:1/20:4)**: $[M+H]^+$ 586.5167, $[So\ (d18:1)+H]^+$ 300.2854, $[So\ (d18:1)-H_2O+H]^+$ 282.2756, $[So\ (d18:1)-2H_2O+H]^+$ 264.2686, the collision energy was set as 40eV.

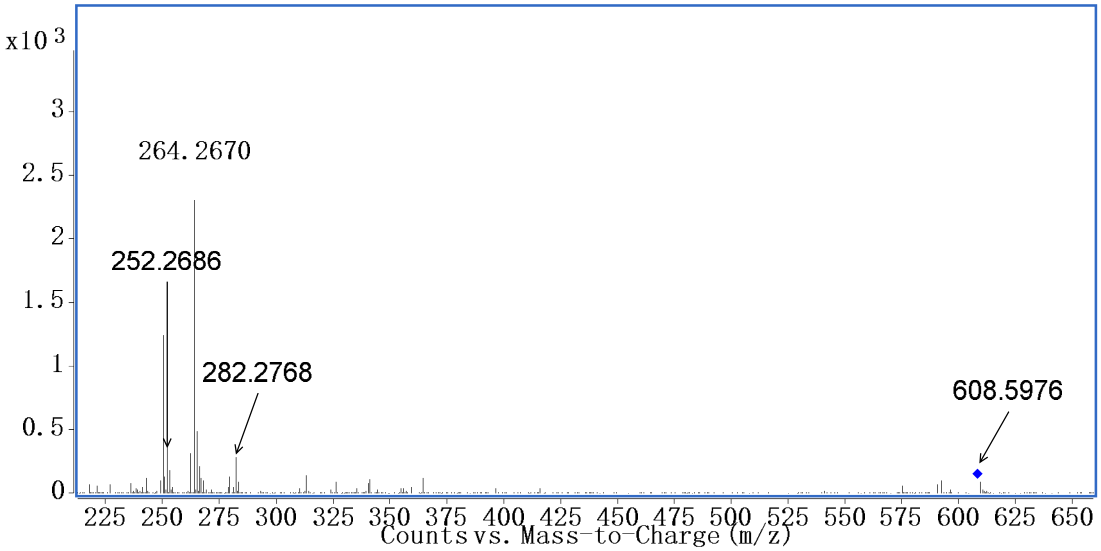

No.33 **Cer (d18:1/21:0)**: $[M+H]^+$ 608.5976, $[So\ (d18:1)-H_2O+H]^+$ 282.2768, $[So\ (d18:1)-2H_2O+H]^+$ 264.2670, $[So\ (d18:1)-H_2O-HCHO+H]^+$ 252.2686, the collision energy was set as 40eV.

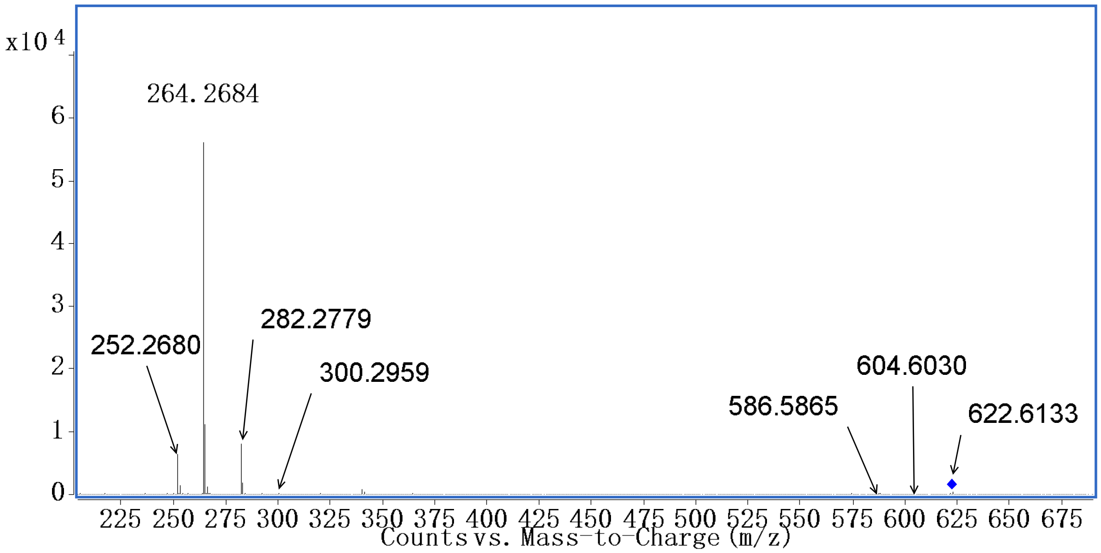

No.34 **Cer (d18:1/22:0)**: $[M+H]^+$ 622.6133, $[M-H_2O+H]^+$ 604.6030, $[M-2H_2O+H]^+$ 586.5865, [So (d18:1)+H]$^+$ 300.2959, [So (d18:1)-H_2O+H]$^+$ 282.2779, [So (d18:1)-$2H_2O$+H]$^+$ 264.2684, [So (d18:1)-H_2O-HCHO+H]$^+$ 252.2680, the collision energy was set as 40eV.

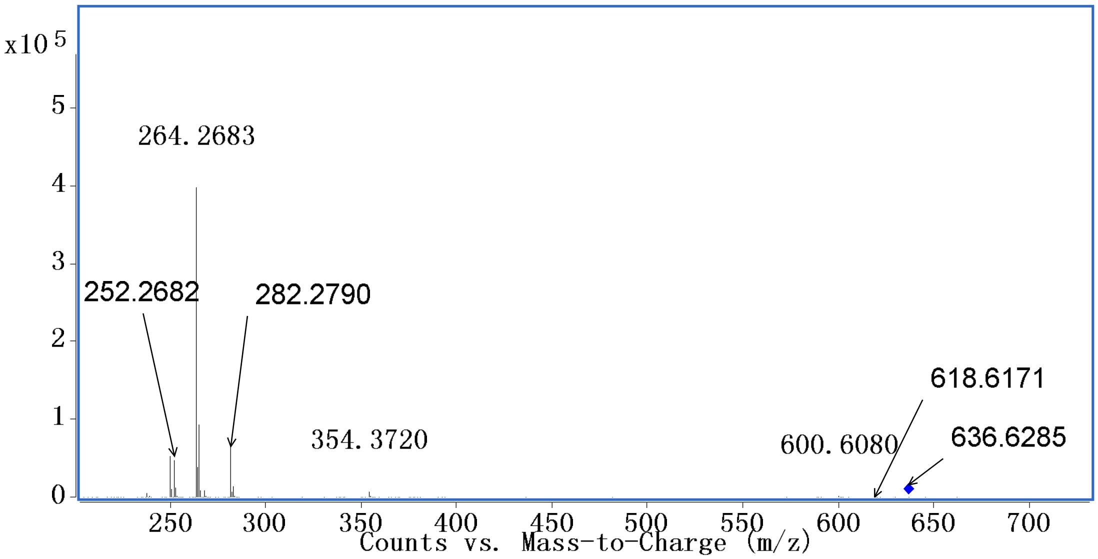

No.35 **Cer (d18:1/23:0)**: $[M+H]^+$ 636.6285, $[M-H_2O+H]^+$ 618.6171, $[M-2H_2O+H]^+$ 600.6080, [So (d18:1)-H_2O+H]$^+$ 282.2790, [So (d18:1)-$2H_2O$+H]$^+$ 264.2683, [So (d18:1)-H_2O-HCHO+H]$^+$ 252.2682, the collision energy was set as 40eV.

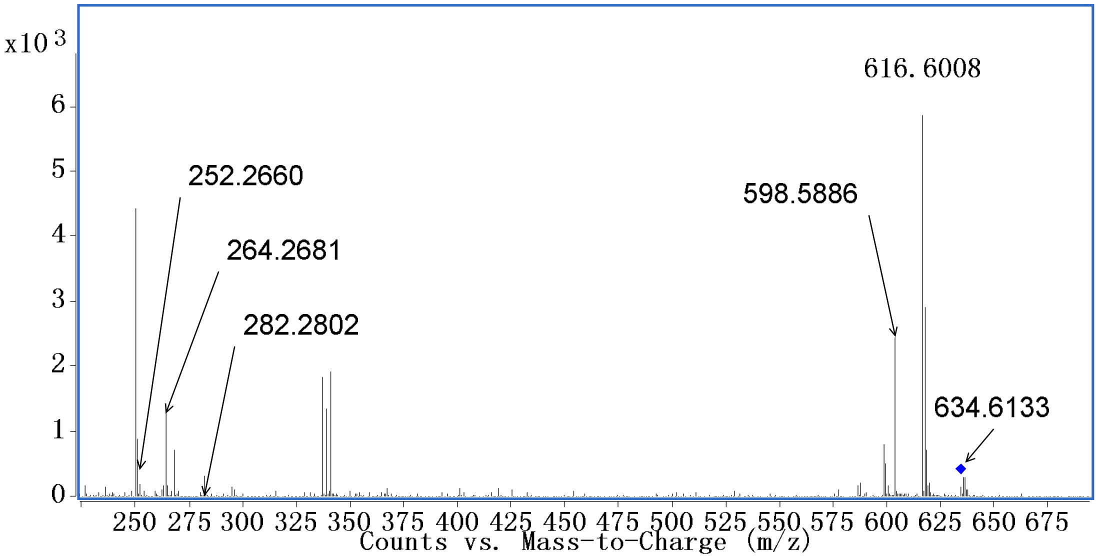

No.36 **Cer (d18:1/23:1)**: $[M+H]^+$ 634.6133, $[M-H_2O+H]^+$ 616.6008, $[M-2H_2O+H]^+$ 598.5886, [So (d18:1)-H_2O+H]$^+$ 282.2802, [So (d18:1)-$2H_2O$+H]$^+$ 264.2681, [So (d18:1)-H_2O-HCHO+H]$^+$ 252.2660, the collision energy was set as 20eV.

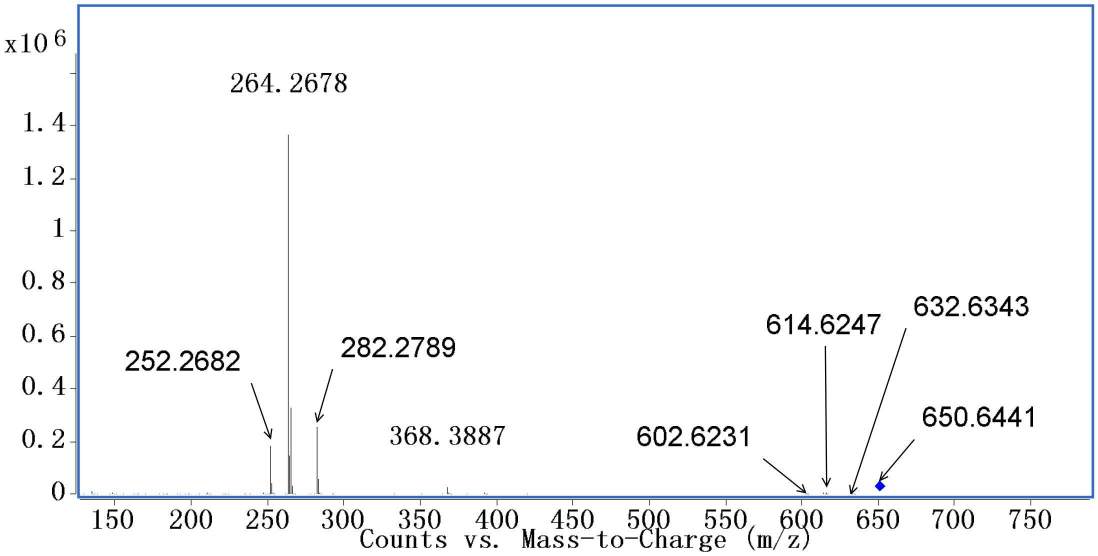

No.37 **Cer (d18:1/24:0)**: $[M+H]^+$ 650.6441, $[M-H_2O+H]^+$ 632.6343, $[M-2H_2O+H]^+$ 614.6247, $[M-H_2O-HCHO+H]^+$ 602.6231, [So (d18:1)-H_2O+H]$^+$ 282.2789, [So (d18:1)-$2H_2O$+H]$^+$ 264.2678, [So (d18:1)-H_2O-HCHO+H]$^+$ 252.2682, the collision energy was set as 40eV.

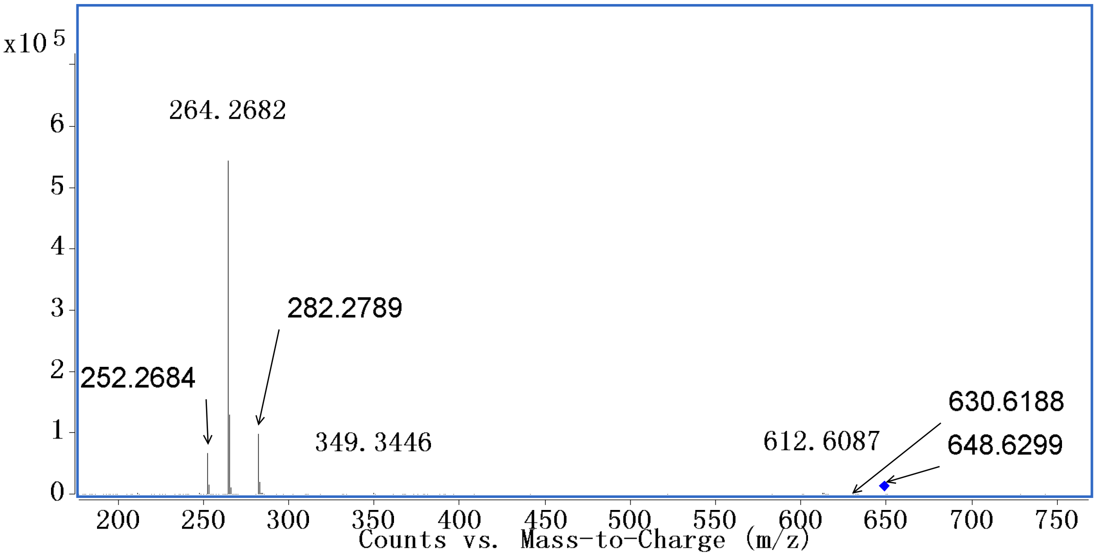

No.38 **Cer (d18:1/24:1)**: $[M+H]^+$ 648.6299, $[M-H_2O+H]^+$ 630.6188, $[M-2H_2O+H]^+$ 612.6087, [So (d18:1)-H_2O+H]$^+$ 282.2789, [So (d18:1)-$2H_2O$+H]$^+$ 264.2682, [So (d18:1)-H_2O-HCHO+H]$^+$ 252.2684, the collision energy was set as 40eV.

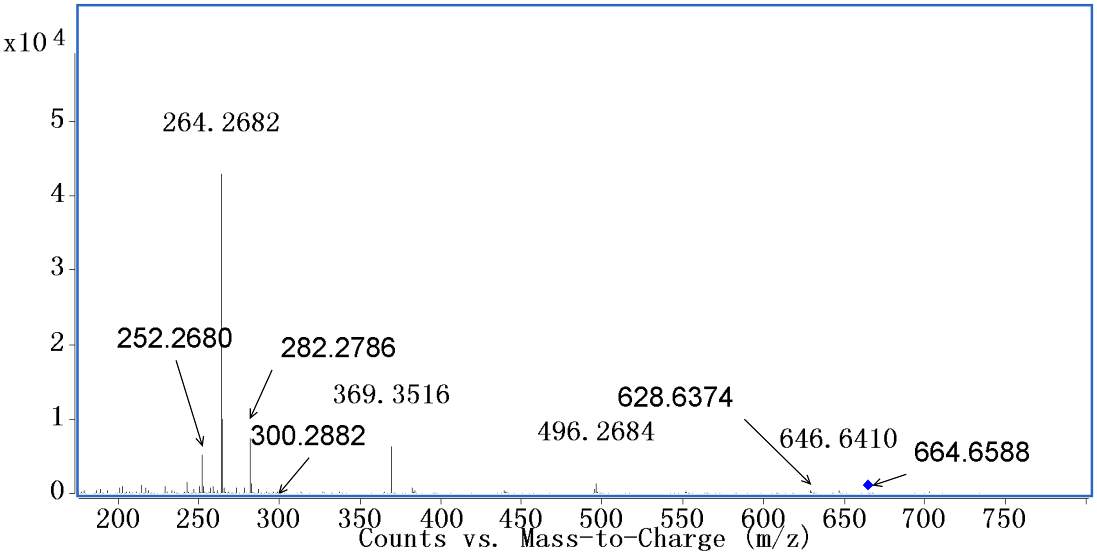

No.39 **Cer (d18:1/25:0)**: $[M+H]^+$ 664.6588, $[M-H_2O+H]^+$ 646.6410, $[M-2H_2O+H]^+$ 628.6374, [So (d18:1)+H]$^+$ 300.2882, [So (d18:1)-H_2O+H]$^+$ 282.2786, [So (d18:1)-$2H_2O$+H]$^+$ 264.2682, [So (d18:1)-H_2O-HCHO+H]$^+$ 252.2680, the collision energy was set as 40eV.

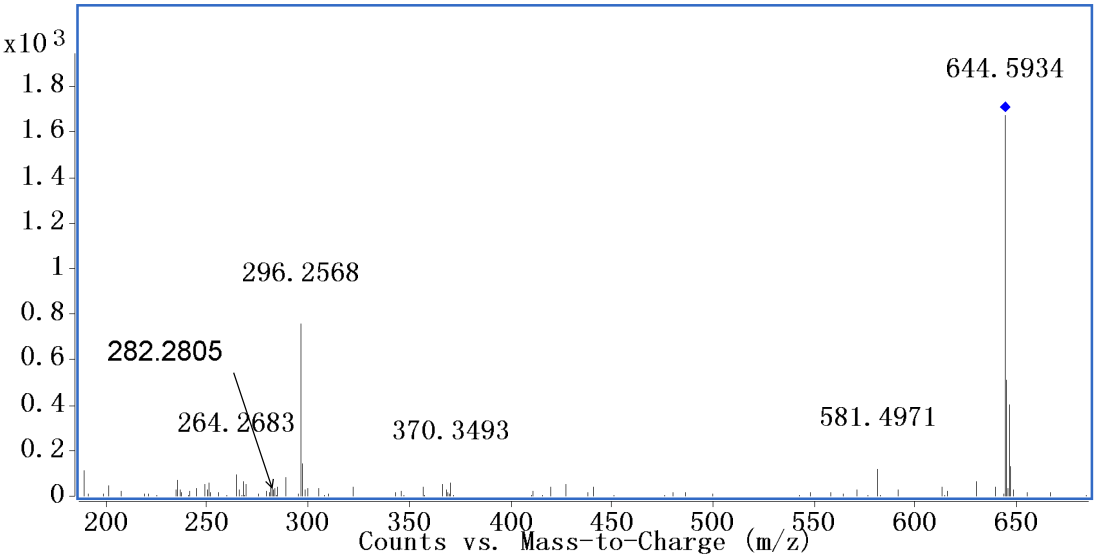

No.40 **Cer (d18:1/26:3)**: $[M+H]^+$ 644.5934, [So (d18:1)-$H_2O+H]^+$ 282.2805, [So (d18:1)-$2H_2O+H]^+$ 264.2683, the collision energy was set as 40eV.

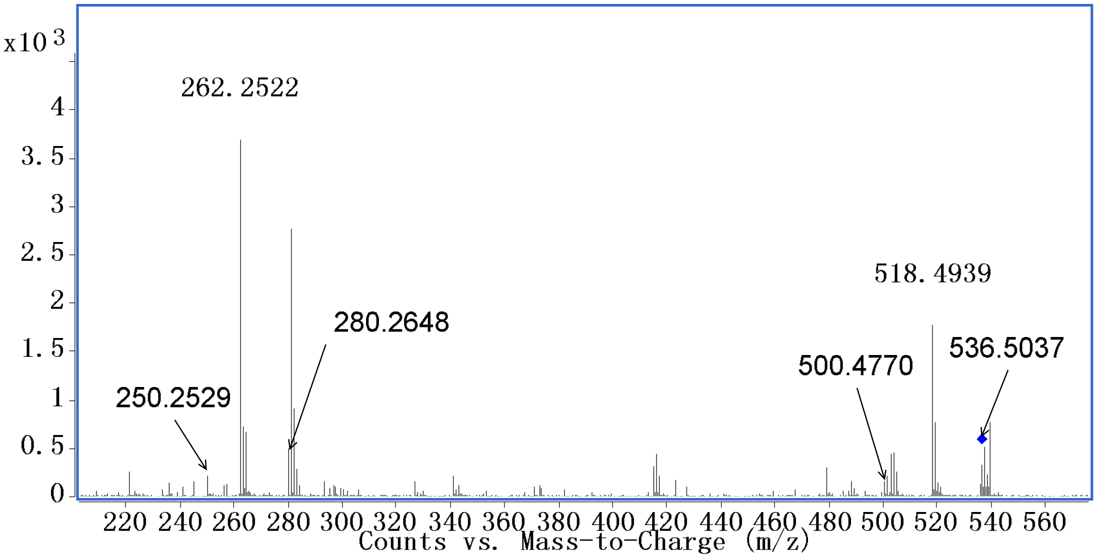

No.41 **Cer (d18:2/16:0)**: $[M+H]^+$ 536.5037, $[M-H_2O+H]^+$ 518.4939, $[M-2H_2O+H]^+$ 500.4770, [So (d18:2)-$H_2O+H]^+$ 280.2648, [So (d18:2)-$2H_2O+H]^+$ 262.2522, [So (d18:2)-H_2O-$HCHO+H]^+$ 250.2529, the collision energy was set as 20eV.

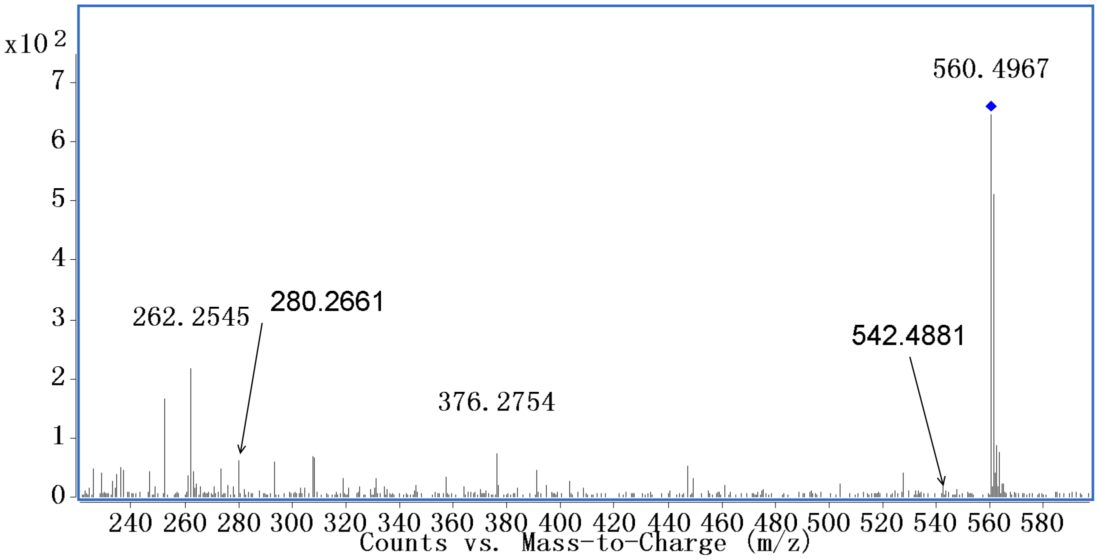

No.42 **Cer (d18:2/18:2)**: $[M+H]^+$ 560.4967, $[M-H_2O+H]^+$ 542.4881, [So (d18:2)-$H_2O+H]^+$ 280.2661, [So (d18:2)-$2H_2O+H]^+$ 262.2545, the collision energy was set as 40eV.

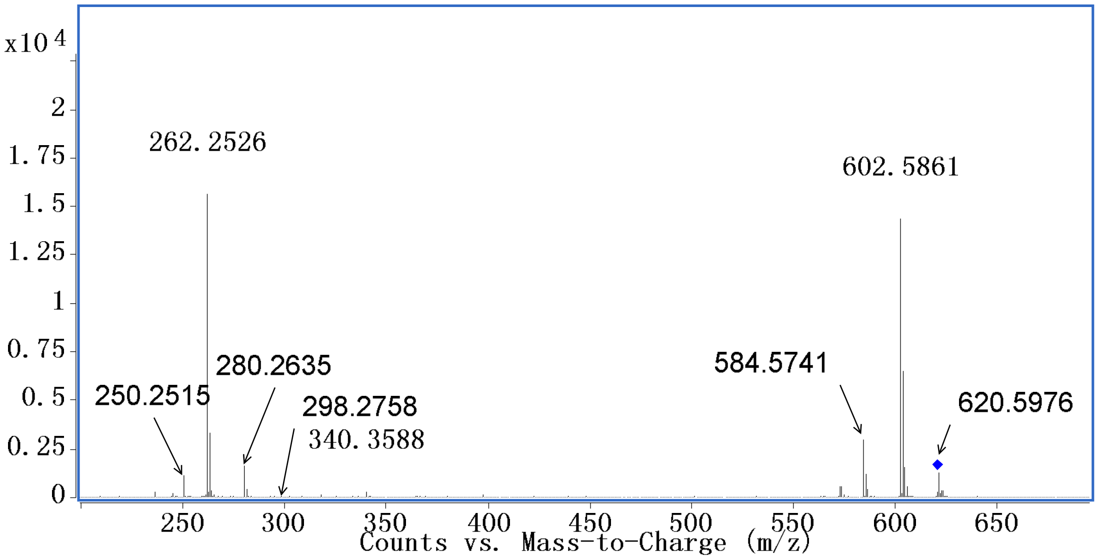

No.43 **Cer (d18:2/22:0)**: $[M+H]^+$ 620.5976, $[M-H_2O+H]^+$ 602.5861, $[M-2H_2O+H]^+$ 584.5741, [So (d18:2)+H]$^+$ 298.2758, [So (d18:2)-$H_2O+H]^+$ 280.2635, [So (d18:2)-$2H_2O+H]^+$ 262.2526, [So (d18:2)-H_2O-HCHO+H]$^+$ 250.2515, the collision energy was set as 20eV.

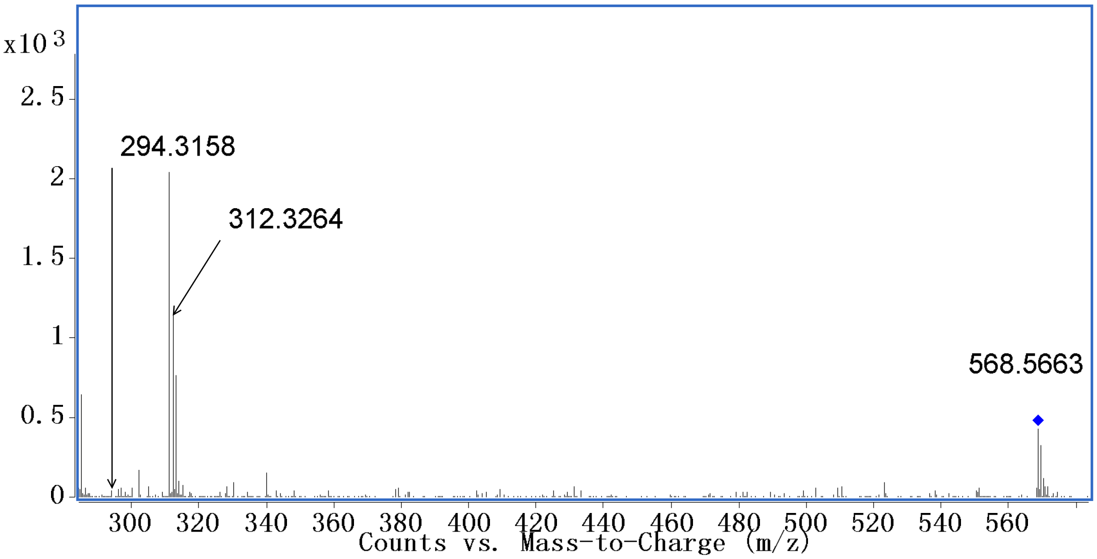

No.44 **Cer (d20:0/16:0)**: $[M+H]^+$ 568.5663, [Sa (d20:0)-H_2O+H]$^+$ 312.3264, [Sa (d20:0)-$2H_2O$+H]$^+$ 294.3158, the collision energy was set as 40eV.

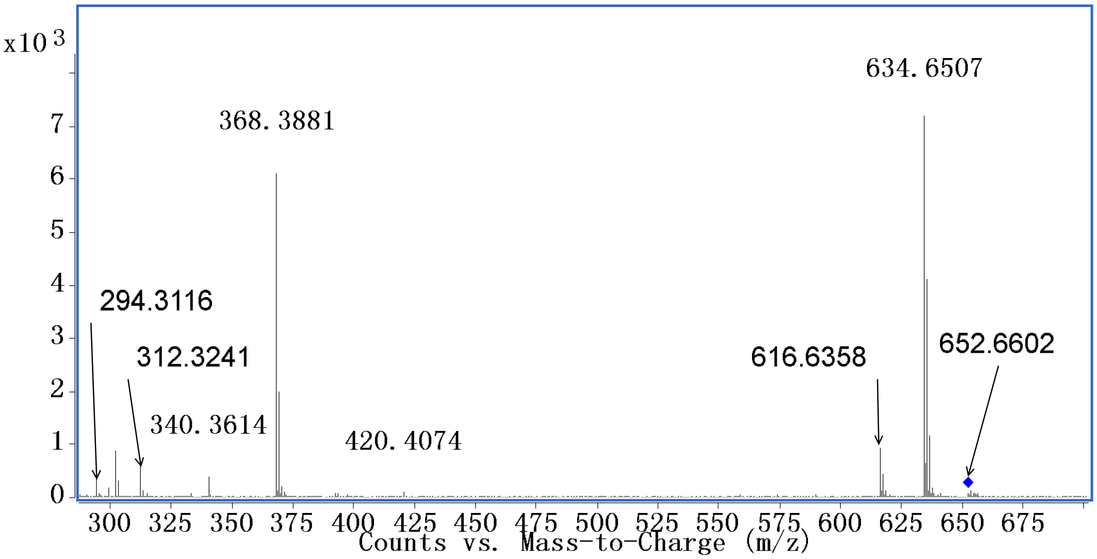

No.45 **Cer (d20:0/22:0)**: $[M+H]^+$ 652.6602, $[M-H_2O+H]^+$ 634.6507, $[M-2H_2O+H]^+$ 616.6358, [Sa (d20:0)-H_2O+H]$^+$ 312.3241, [Sa (d20:0)-$2H_2O$+H]$^+$ 294.3116, the collision energy was set as 40eV.

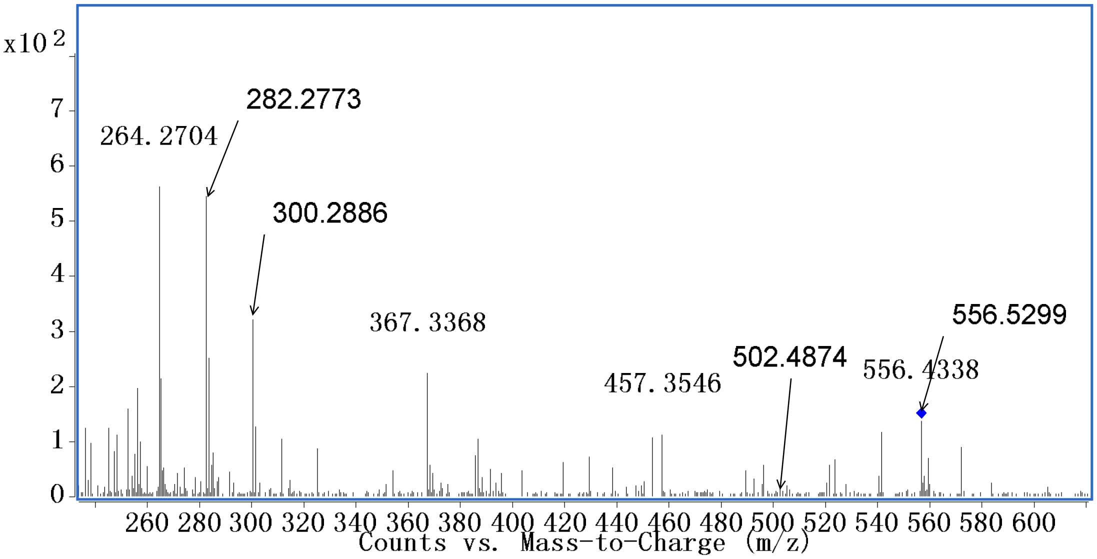

No.46 **Cer (t18:0/16:0)**: $[M+H]^+$ 556.5299, $[M-3H_2O+H]^+$ 502.4874, [Sa (t18:0)-H_2O+H]$^+$ 300.2886, [Sa (t18:0)-$2H_2O$+H]$^+$ 282.2773, [Sa (t18:0)-$3H_2O$+H]$^+$ 264.2704, the collision energy was set as 40eV.

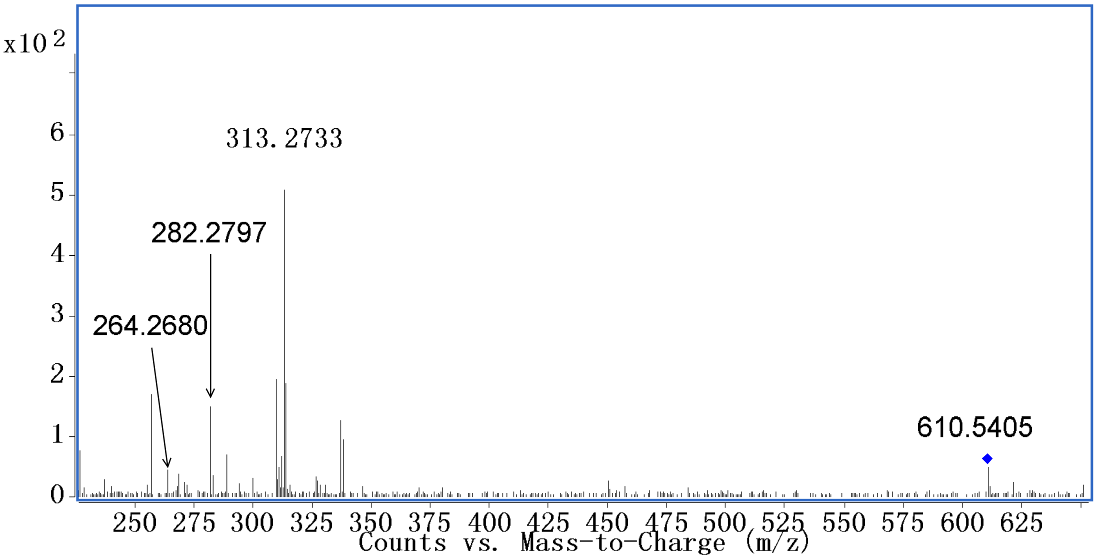

No.47 **Cer (t18:0/19:2)**: $[M+H]^+$ 610.5405, [Sa (t18:0)-$2H_2O$+H]$^+$ 282.2797, [Sa (t18:0)-$3H_2O$+H]$^+$ 264.2680, the collision energy was set as 40eV.

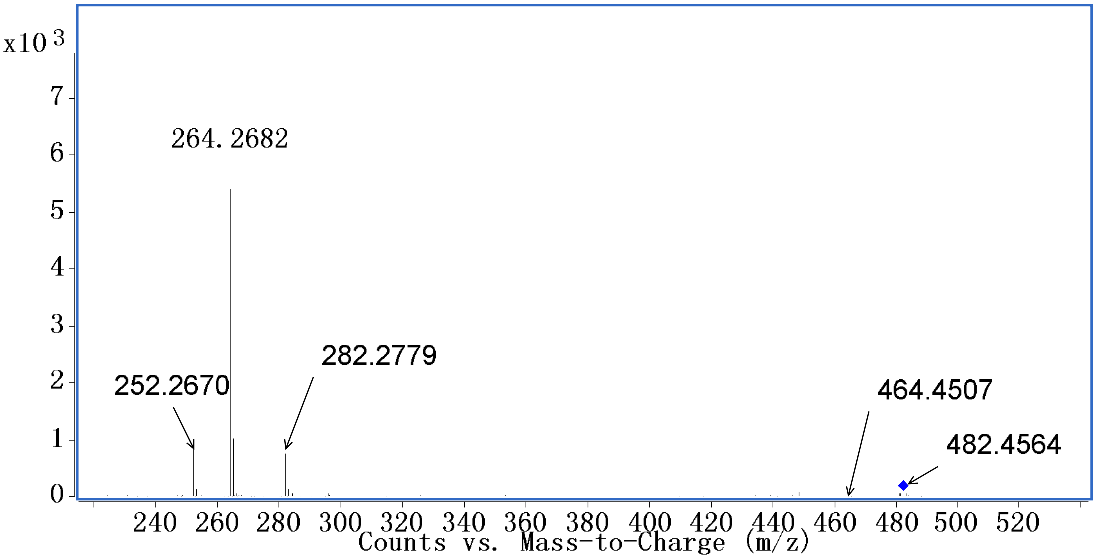

[IS-4] **Cer (d18:1/12:0)**: $[M+H]^+$ 482.4564, $[M-H_2O+H]^+$ 464.4507, [So (d18:1)-$H_2O+H]^+$ 282.2779, [So (d18:1)-$2H_2O+H]^+$ 264.2682, [So (d18:1)-H_2O-$HCHO+H]^+$ 252.2670, the collision energy was set as 40eV.

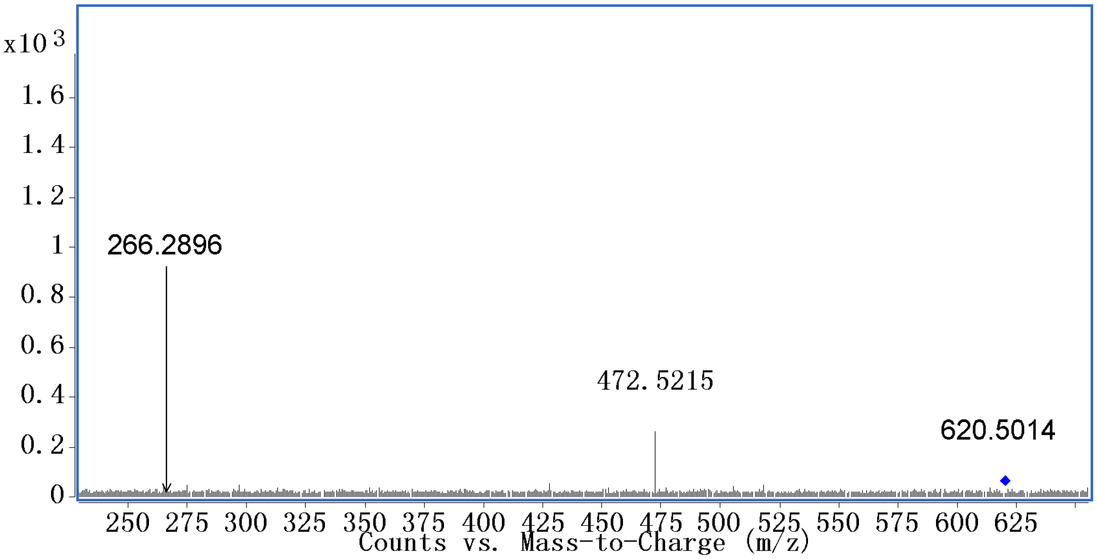

No.48 **C1P (d18:0/16:0)**: $[M+H]^+$ 620.5014, [Sa (d18:0)-$2H_2O+H]^+$ 266.2896, the collision energy was set as 40eV.

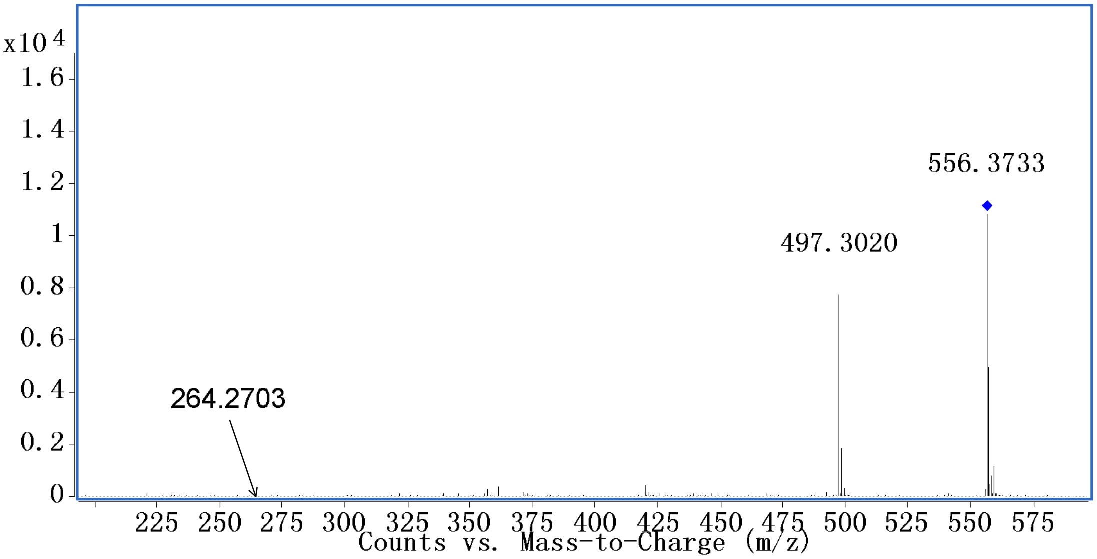

No.49 **C1P (d18:1/12:3)**: $[M+H]^+$ 556.3733, [So (d18:1)-$2H_2O$+H]$^+$ 264.2703, the collision energy was set as 40eV.

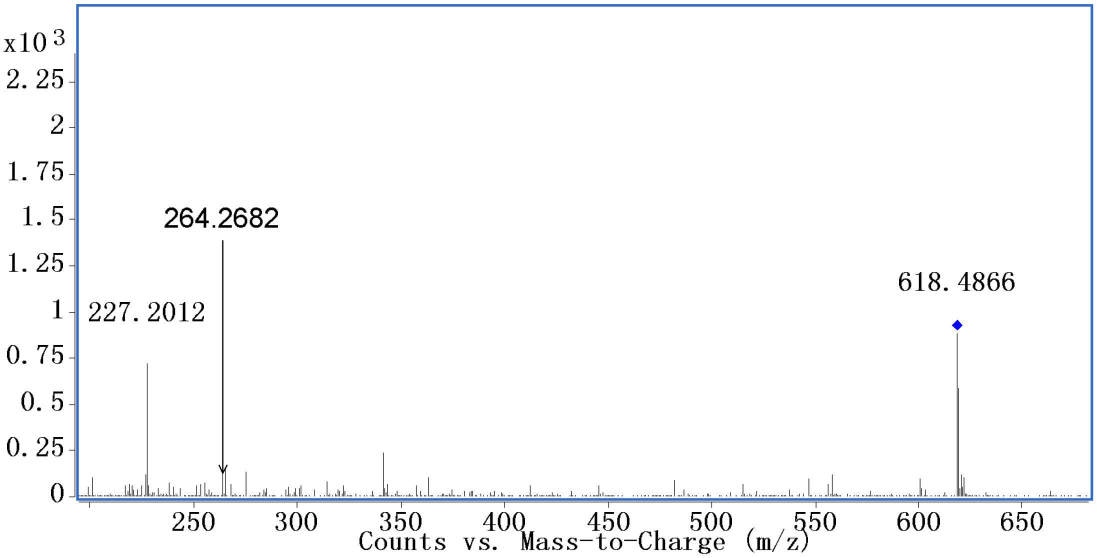

No.50 **C1P (d18:1/16:0)**: $[M+H]^+$ 618.4866, [So (d18:1)-$2H_2O$+H]$^+$ 264.2682, the collision energy was set as 40eV.

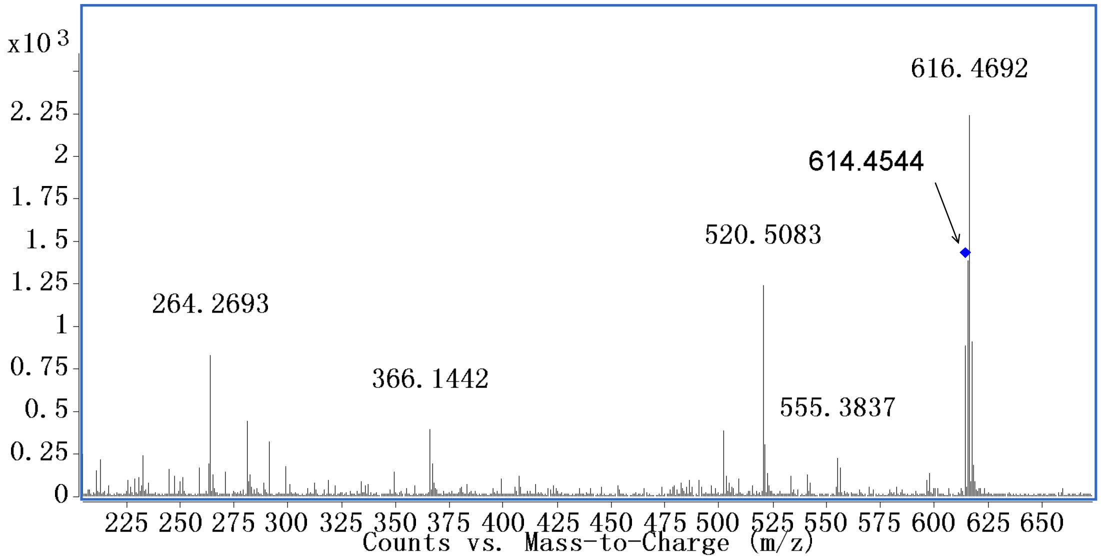

No.51 **C1P (d18:1/16:2)**: $[M+H]^+$ 614.4544, [So (d18:1)-$2H_2O$+H]$^+$ 264.2693, the collision energy was set as 40eV.

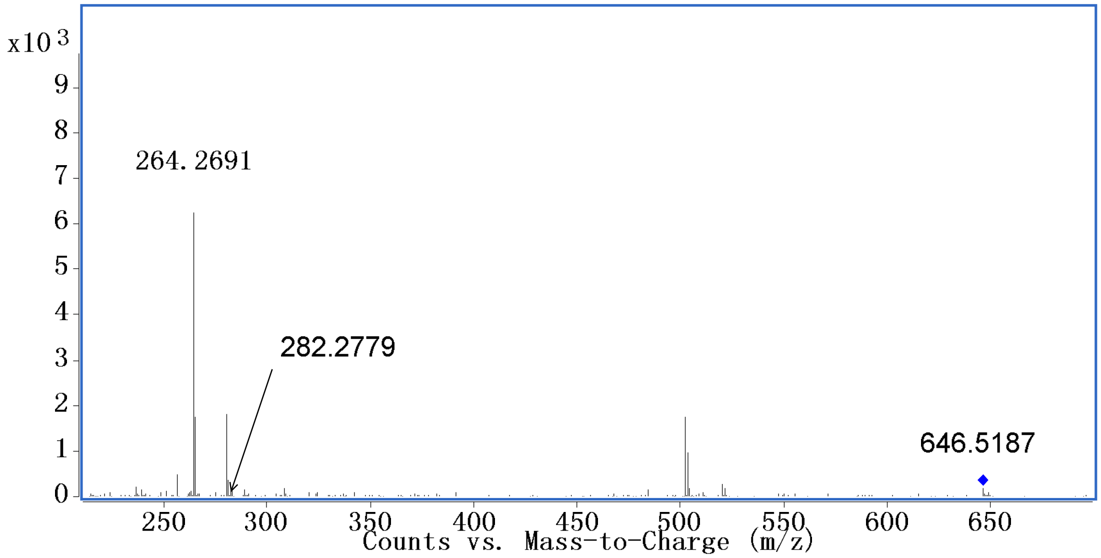

No.52 **C1P (d18:1/18:0)**: $[M+H]^+$ 646.5187, [So (d18:1)-H_2O+H]$^+$ 282.2779, [So (d18:1)-$2H_2O$+H]$^+$ 264.2691, the collision energy was set as 40eV.

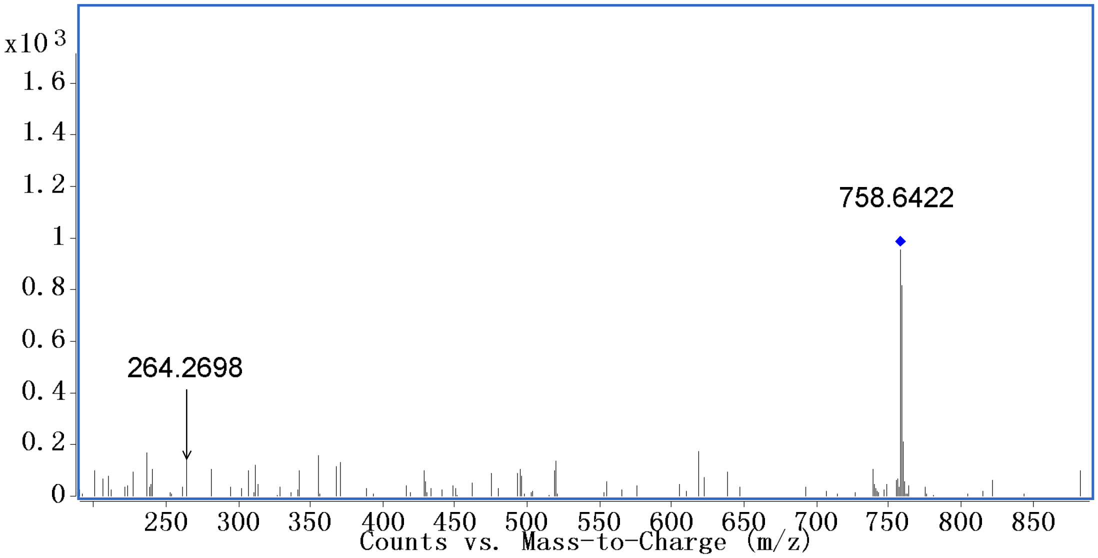

No.53 **C1P (d18:1/26:0)**: $[M+H]^+$ 758.6422, [So (d18:1)-$2H_2O$+H]$^+$ 264.2698, the collision energy was set as 40eV.

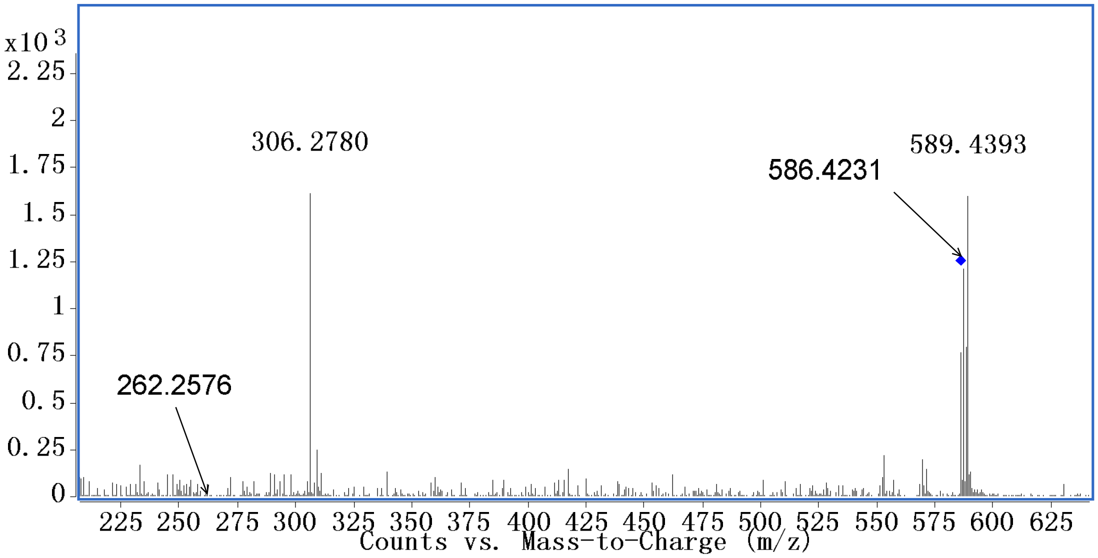

No.54 **C1P (d18:2/14:1)**: $[M+H]^+$ 586.4231, [So (d18:2)-$2H_2O$+H]$^+$ 262.2576, the collision energy was set as 40eV.

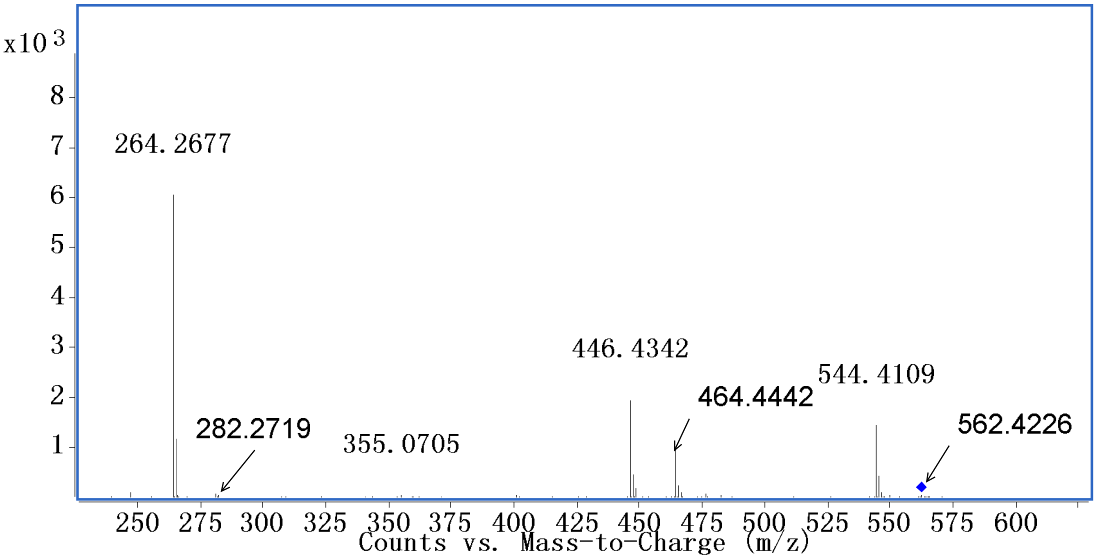

[IS-5] **C1P (d18:1/12:0)**: $[M+H]^+$ 562.4226, $[M-H_2O+H]^+$ 544.4109, $[M-H_3PO_4+H]^+$ 464.4442, $[M-H_3PO_4-H2O+H]^+$ 446.4342, [So (d18:1)-H_2O+H]$^+$ 282.2719, [So (d18:1)-2H_2O+H]$^+$ 264.2677, the collision energy was set as 40eV.

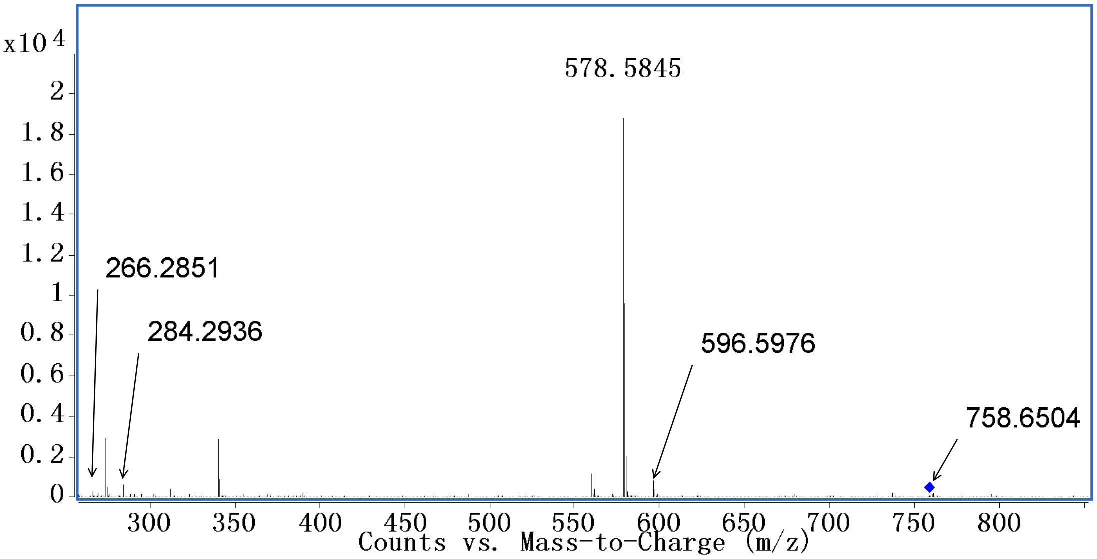

No.55 **HexCer (d18:0/20:0)**: $[M+H]^+$ 758.6504, $[M-Hex+H]^+$ 596.5976, $[M-Hex-H_2O+H]^+$ 578.5845, [Sa (d18:0)-H_2O+H]$^+$ 284.2936, [Sa (d18:0)-2H_2O+H]$^+$ 266.2851, the collision energy was set as 40eV.

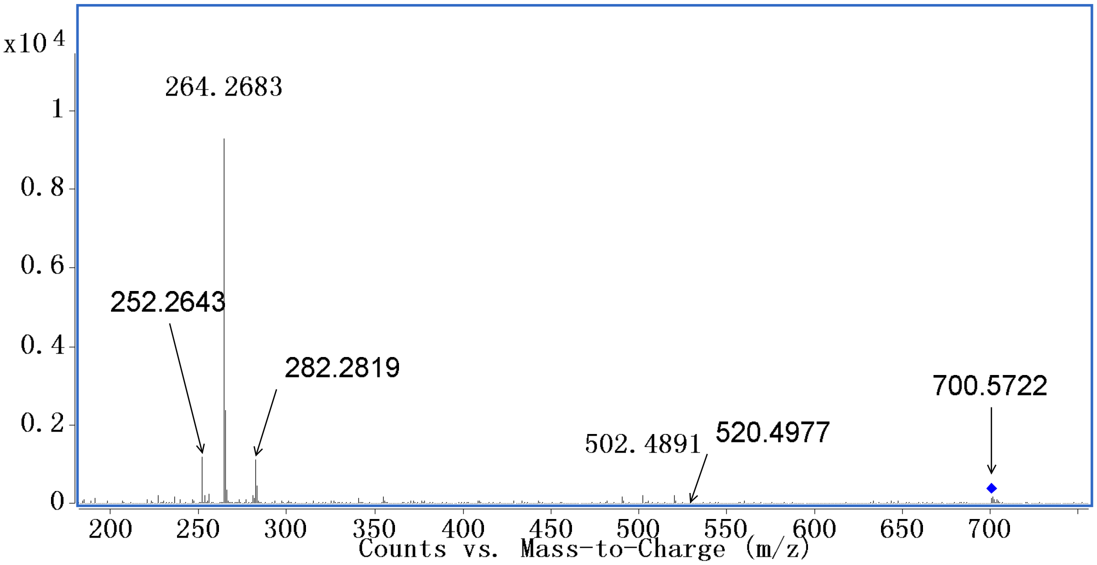

No.56 **HexCer (d18:1/16:0)**: $[M+H]^+$ 700.5722, $[M\text{-}Hex\text{-}H_2O+H]^+$ 520.4977, $[M\text{-}Hex\text{-}2H_2O+H]^+$ 502.4891, [So (d18:1)-$H_2O+H]^+$ 282.2819, [So (d18:1)-$2H_2O+H]^+$ 264.2683, [So (d18:1)-H_2O-HCHO+H$]^+$ 252.2643, the collision energy was set as 40eV.

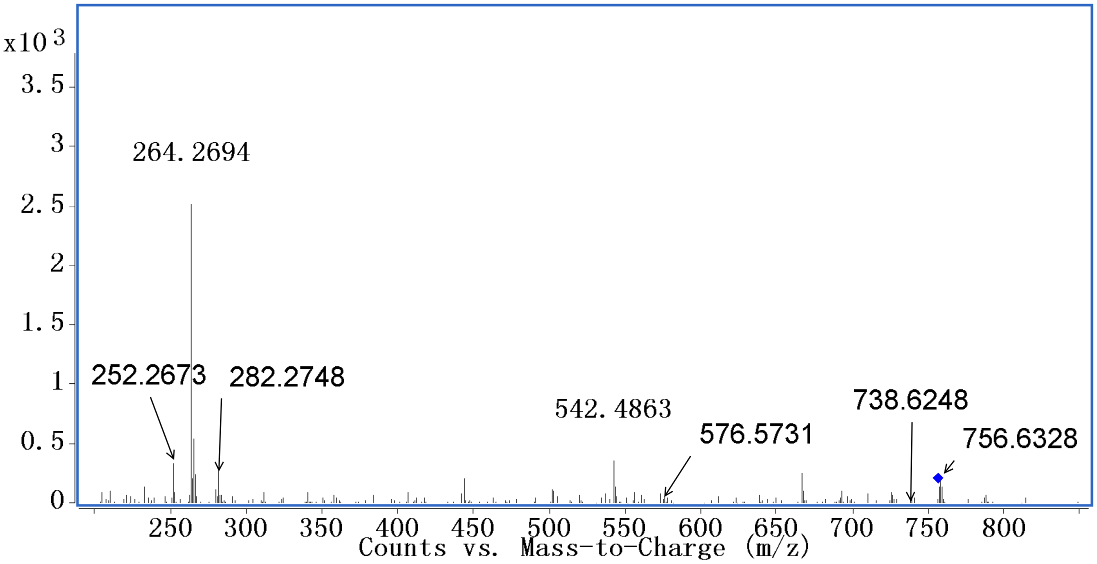

No.57 **HexCer (d18:1/20:0)**: $[M+H]^+$ 756.6328, $[M\text{-}H_2O+H]^+$ 738.6248, $[M\text{-}Hex\text{-}H_2O+H]^+$ 576.5731, [So (d18:1)-$H_2O+H]^+$ 282.2748, [So (d18:1)-$2H_2O+H]^+$ 264.2694, [So (d18:1)-H_2O-HCHO+H$]^+$ 252.2673, the collision energy was set as 40eV.

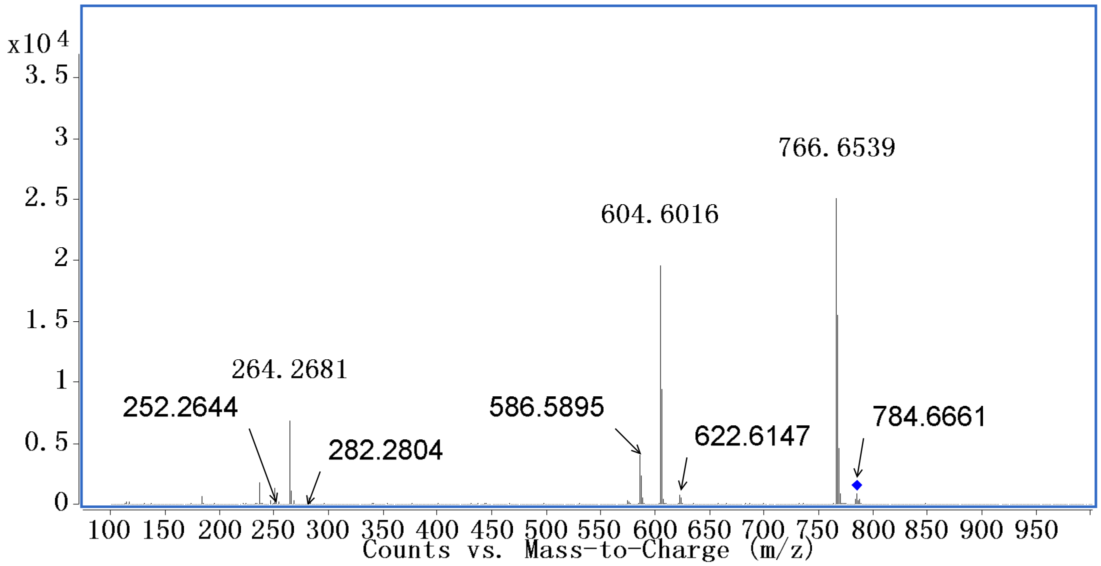

No.58 **HexCer (d18:1/22:0)**: [M+H]$^+$ 784.6661, [M-H$_2$O+H]$^+$ 766.6539, [M-Hex+H]$^+$ 622.6147, [M-Hex-H$_2$O+H]$^+$ 604.6016, [M-Hex-2H$_2$O+H]$^+$ 586.5895, [So (d18:1)-H$_2$O+H]$^+$ 282.2804, [So (d18:1)-2H$_2$O+H]$^+$ 264.2681, [So (d18:1)-H$_2$O-HCHO+H]$^+$ 252.2644, the collision energy was set as 20eV.

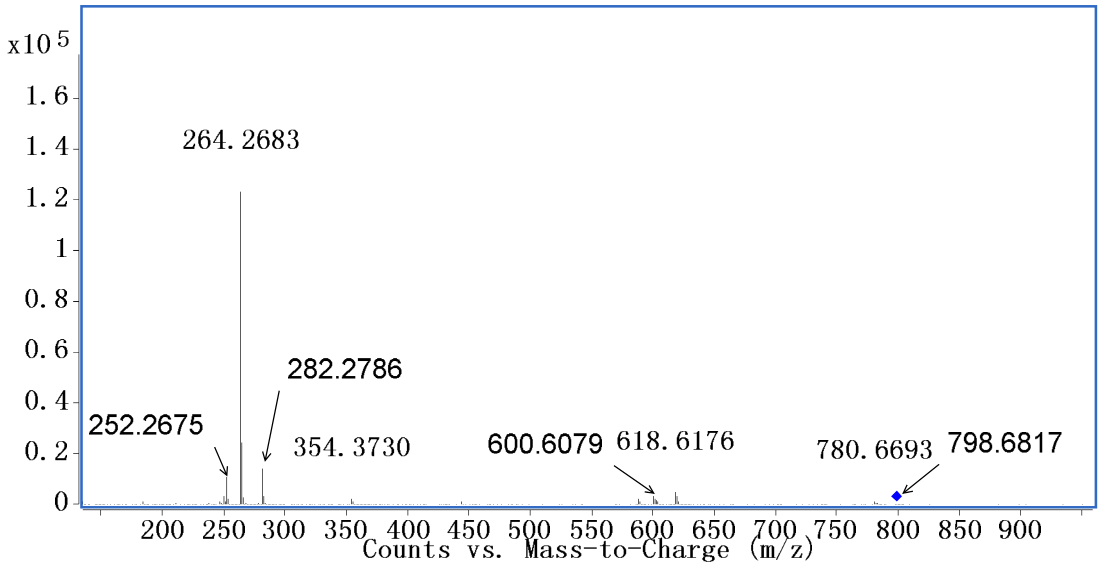

No.59 **HexCer (d18:1/23:0)**: [M+H]$^+$ 798.6817, [M-H$_2$O+H]$^+$ 780.6693, [M-Hex-H$_2$O+H]$^+$ 618.6176, [M-Hex-2H$_2$O+H]$^+$ 600.6079, [So (d18:1)-H$_2$O+H]$^+$ 282.2786, [So (d18:1)-2H$_2$O+H]$^+$ 264.2683, [So (d18:1)-H$_2$O-HCHO+H]$^+$ 252.2675, the collision energy was set as 40eV.

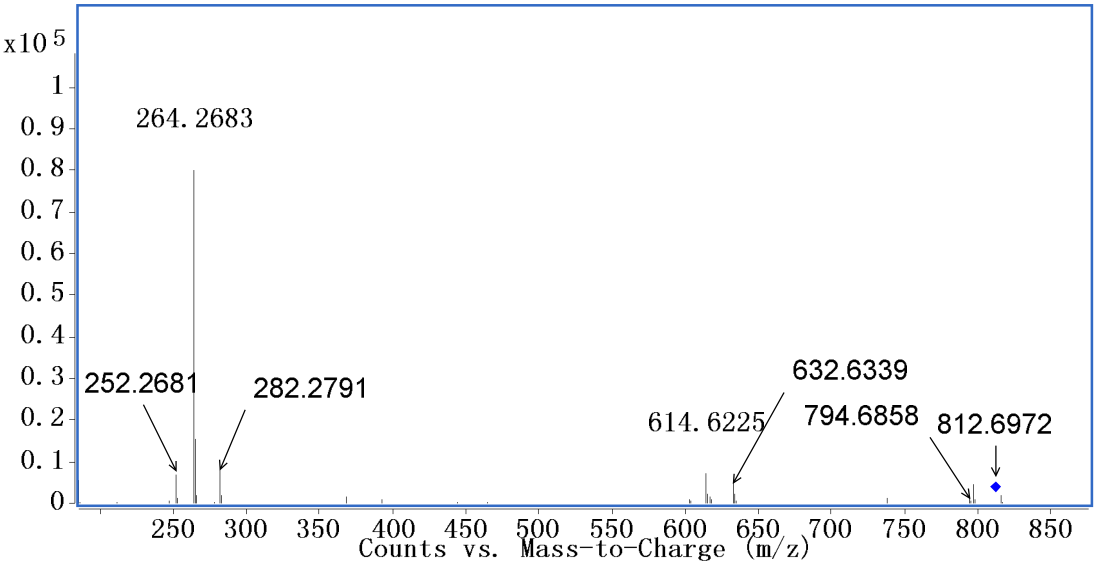

No.60 **HexCer (d18:1/24:0)**: $[M+H]^+$ 812.6972, $[M\text{-}H_2O+H]^+$ 794.6858, $[M\text{-}Hex\text{-}H_2O+H]^+$ 632.6339, $[M\text{-}Hex\text{-}2H_2O+H]^+$ 614.6225, [So (d18:1)-$H_2O+H]^+$ 282.2791, [So (d18:1)-$2H_2O+H]^+$ 264.2683, [So (d18:1)-H_2O-HCHO+H$]^+$ 252.2681, the collision energy was set as 40eV.

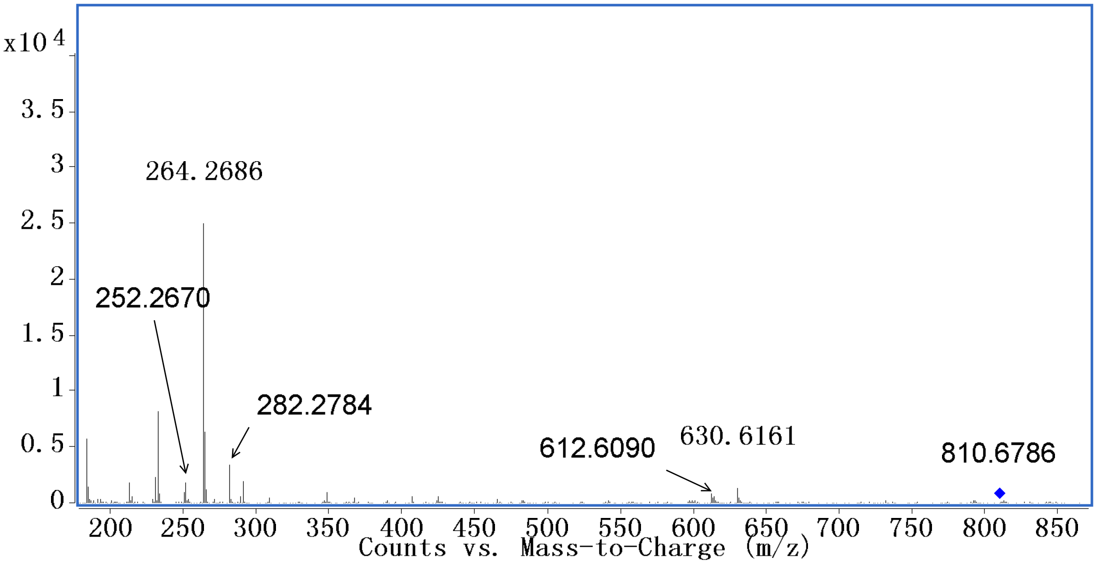

No.61 **HexCer (d18:1/24:1)**: $[M+H]^+$ 810.6786, $[M\text{-}Hex\text{-}H_2O+H]^+$ 630.6161, $[M\text{-}Hex\text{-}2H_2O+H]^+$ 612.6090, [So (d18:1)-$H_2O+H]^+$ 282.2784, [So (d18:1)-$2H_2O+H]^+$ 264.2686, [So (d18:1)-H_2O-HCHO+H$]^+$ 252.2670, the collision energy was set as 40eV.

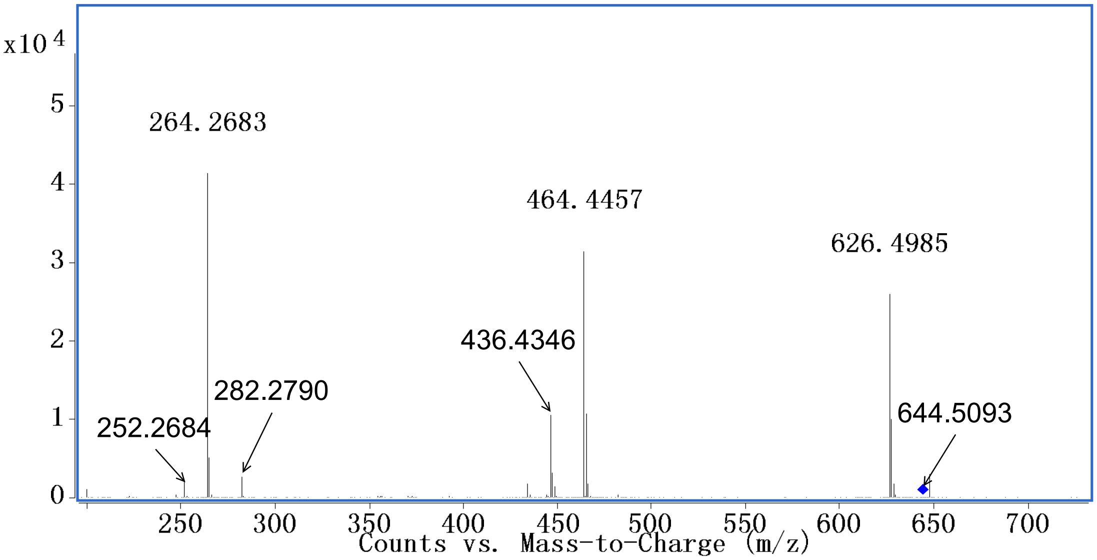

[IS-6] **HexCer (d18:1/12:0)**: $[M+H]^+$ 644.5093, $[M-H_2O+H]^+$ 626.4985, $[M-Hex-H_2O+H]^+$ 464.4457, $[M-Hex-2H_2O+H]^+$ 446.4346, [So (d18:1)-$H_2O+H]^+$ 282.2790, [So (d18:1)-$2H_2O+H]^+$ 264.2683, [So (d18:1)-H_2O-HCHO+H$]^+$ 252.2684, the collision energy was set as 40eV.

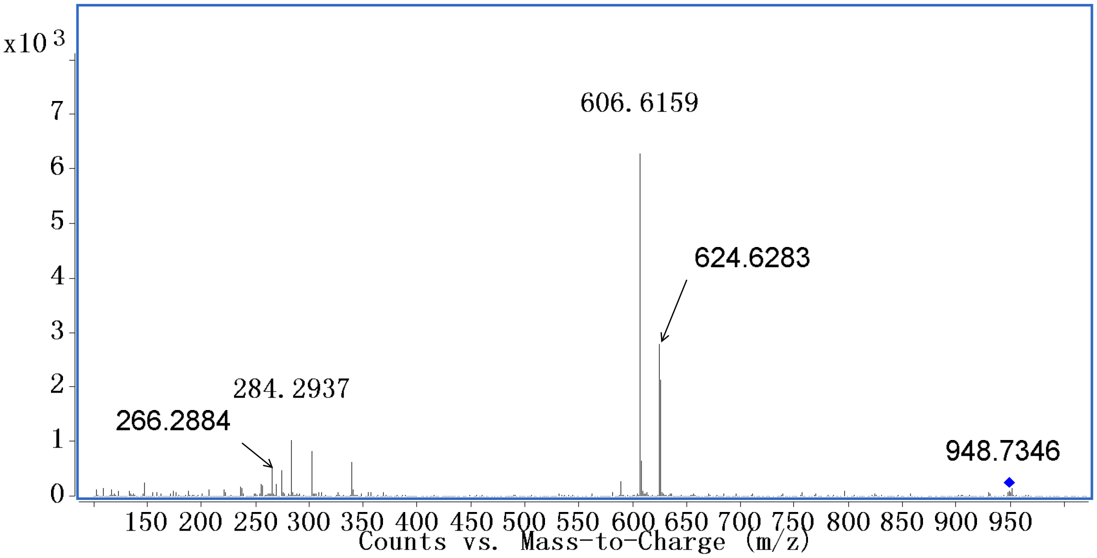

No.62 **LacCer (d18:0/22:0)**: $[M+H]^+$ 948.7346, $[M-2Hex+H]^+$ 624.6283, $[M-2Hex-H2O+H]^+$ 606.6159, [Sa (d18:0)-$H_2O+H]^+$ 284.2937, [Sa (d18:0)-$2H_2O+H]^+$ 266.2884, the collision energy was set as 40eV.

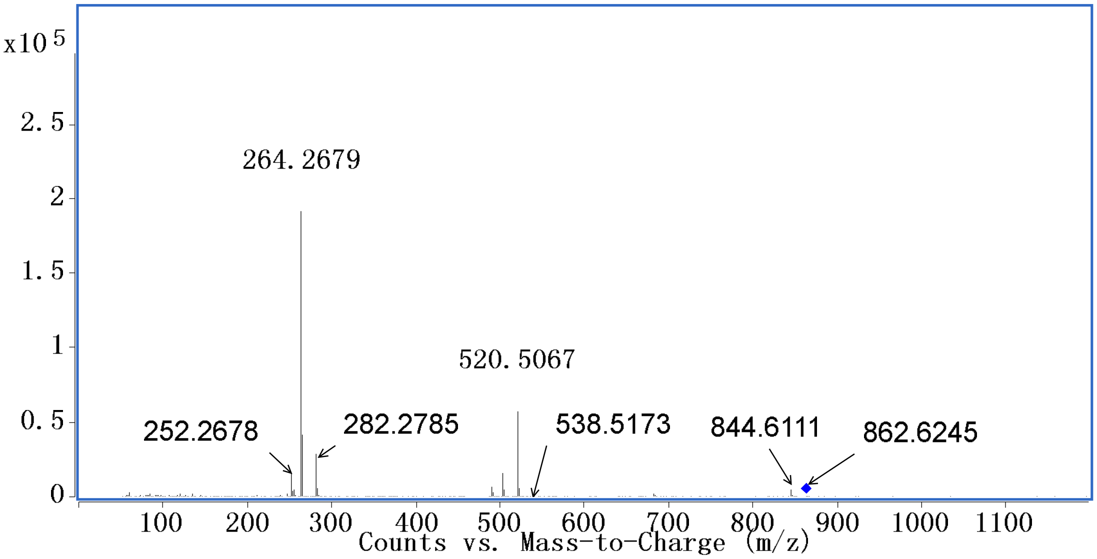

No.63 **LacCer (d18:1/16:0)**: $[M+H]^+$ 862.6245, $[M-H_2O+H]^+$ 844.6111, $[M-2Hex+H]^+$ 538.5173, $[M-2Hex-H_2O+H]^+$ 520.5067, [So (d18:1)-H_2O+H]$^+$ 282.2785, [So (d18:1)-$2H_2O$+H]$^+$ 264.2679, [So (d18:1)-H_2O-HCHO+H]$^+$ 252.2678, the collision energy was set as 40eV.

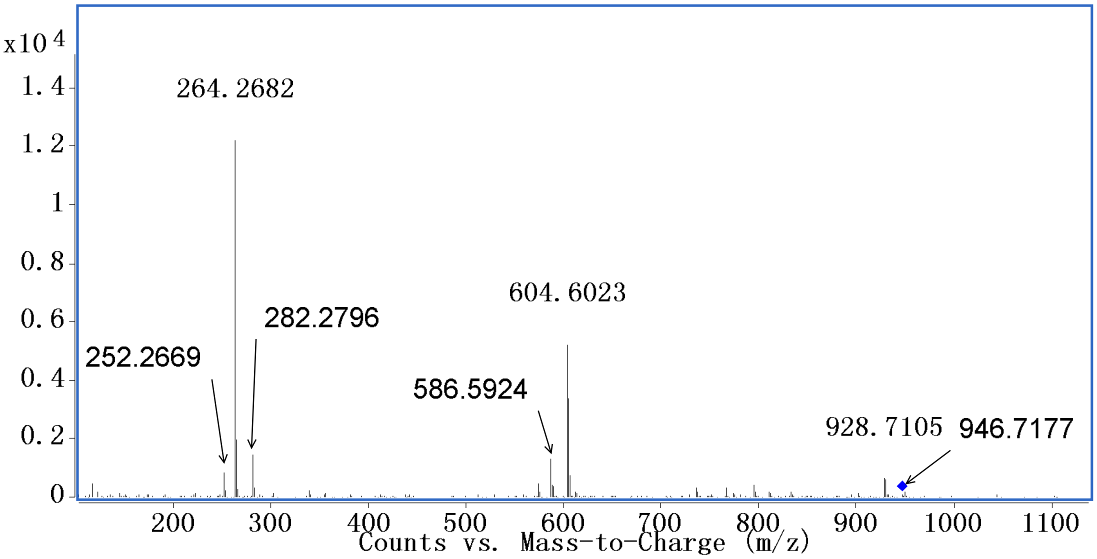

No.64 **LacCer (d18:1/22:0)**: $[M+H]^+$ 946.7177, $[M-H_2O+H]^+$ 928.7105, $[M-2Hex-H_2O+H]^+$ 604.6023, $[M-2Hex-2H_2O+H]^+$ 586.5924, [So (d18:1)-H_2O+H]$^+$ 282.2796, [So (d18:1)-$2H_2O$+H]$^+$ 264.2682, [So (d18:1)-H_2O-HCHO+H]$^+$ 252.2669, the collision energy was set as 40eV.

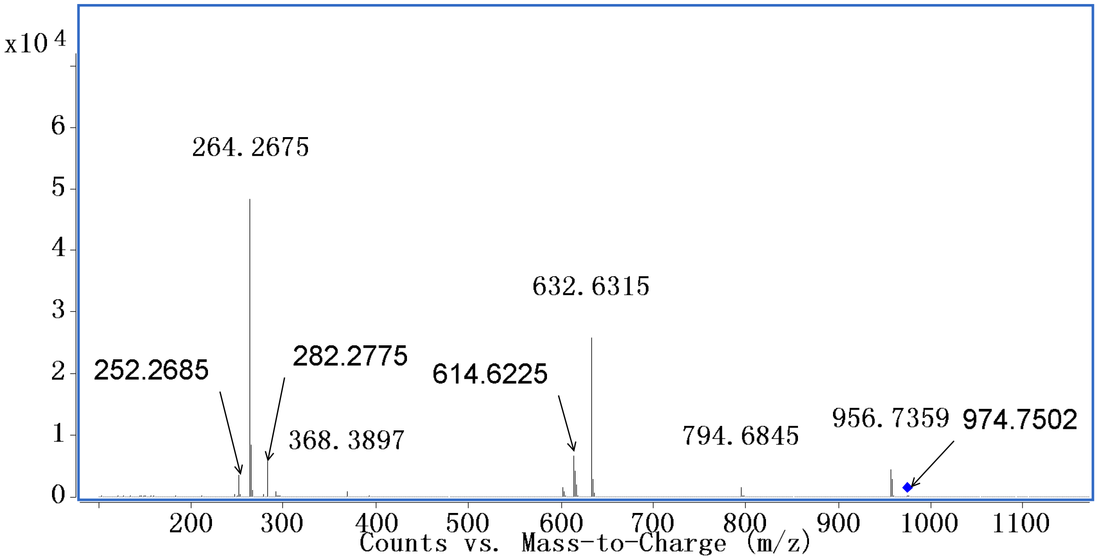

No.65 **LacCer (d18:1/24:0)**: $[M+H]^+$ 974.7502, $[M-H_2O+H]^+$ 956.7359, $[M-Hex-H_2O+H]^+$ 794.6845, $[M-2Hex-H_2O+H]^+$ 632.6315, $[M-2Hex-2H_2O+H]^+$ 614.6225, [So (d18:1)-$H_2O+H]^+$ 282.2775, [So (d18:1)-$2H_2O+H]^+$ 264.2675, [So (d18:1)-$H_2O-HCHO+H]^+$ 252.2685, the collision energy was set as 40eV.

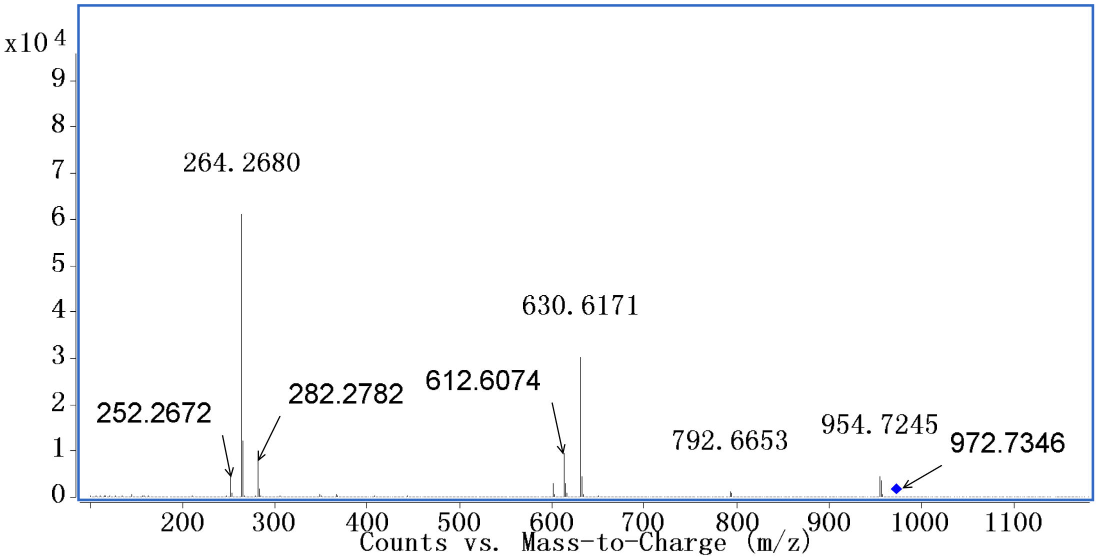

No.66 **LacCer (d18:1/24:1)**: $[M+H]^+$ 972.7346, $[M-H_2O+H]^+$ 954.7245, $[M-Hex-H_2O+H]^+$ 792.6653, $[M-2Hex-H_2O+H]^+$ 630.6171, $[M-2Hex-2H_2O+H]^+$ 612.6074, [So (d18:1)-$H_2O+H]^+$ 282.2782, [So (d18:1)-$2H_2O+H]^+$ 264.2680, [So (d18:1)-$H_2O-HCHO+H]^+$ 252.2672, the collision energy was set as 40eV.

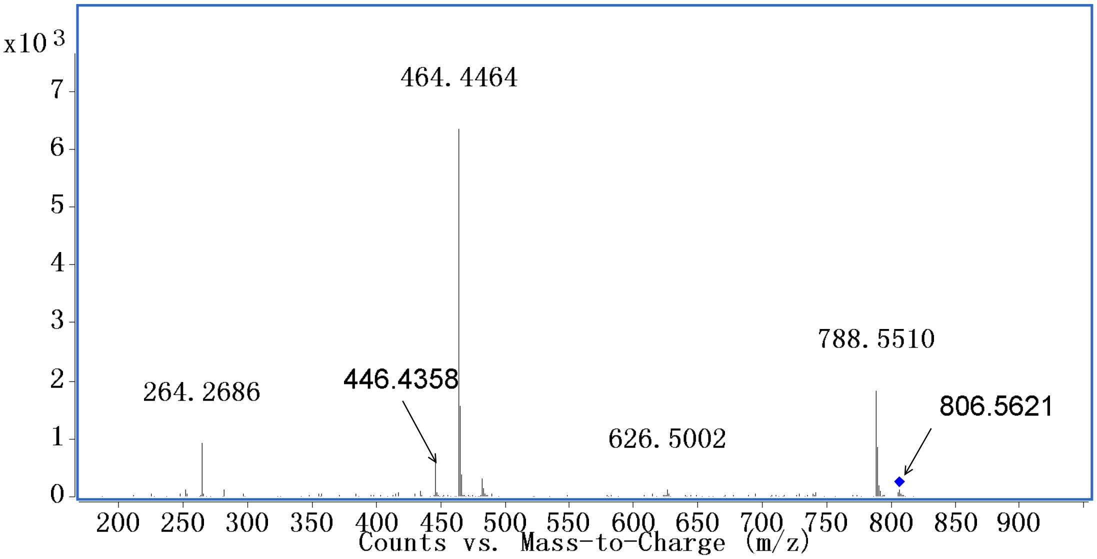

[IS-7] **LacCer (d18:1/12:0)**: $[M+H]^+$ 806.5621, $[M-H_2O+H]^+$ 788.5510, $[M-Hex-H_2O+H]^+$ 626.5002, $[M-2Hex-H_2O+H]^+$ 464.4464, $[M-2Hex-2H_2O+H]^+$ 446.4358, $[So\ (d18:1)-2H_2O+H]^+$ 264.2686, the collision energy was set as 40eV.

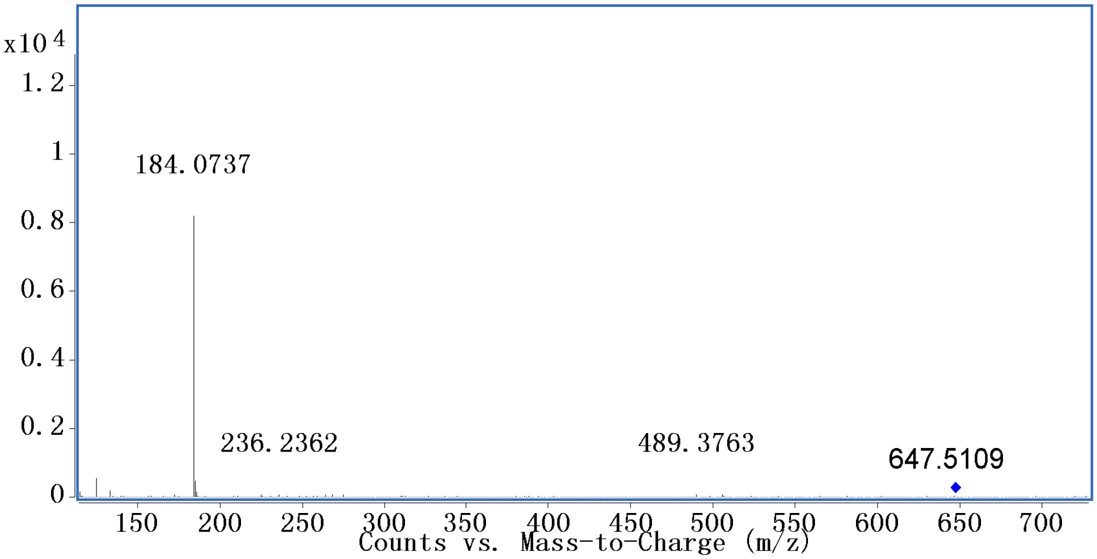

No.67 **SM (d16:1/14:0)**: $[M+H]^+$ 647.5109, $[So\ (d16:1)-2H_2O+H]^+$ 236.2362, $[phosphocholine+H]^+$ 184.0737, the collision energy was set as 40eV.

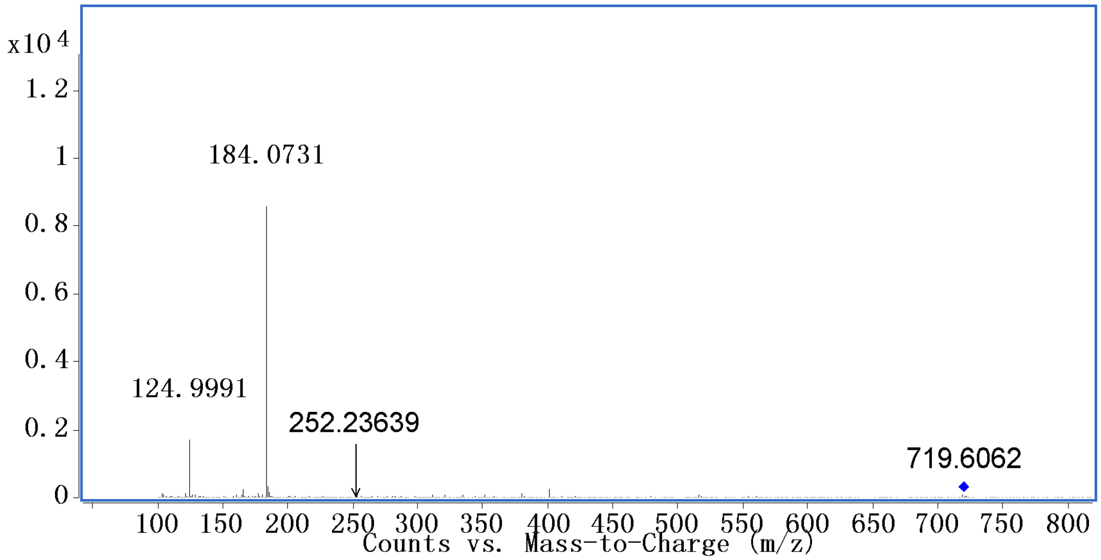

No.68 **SM (d17:0/18:0)**: $[M+H]^+$ 719.6062, [Sa (d17:0)-$2H_2O$+H]$^+$ 252.2691, [phosphocholine+H]$^+$ 184.0731, the collision energy was set as 40eV.

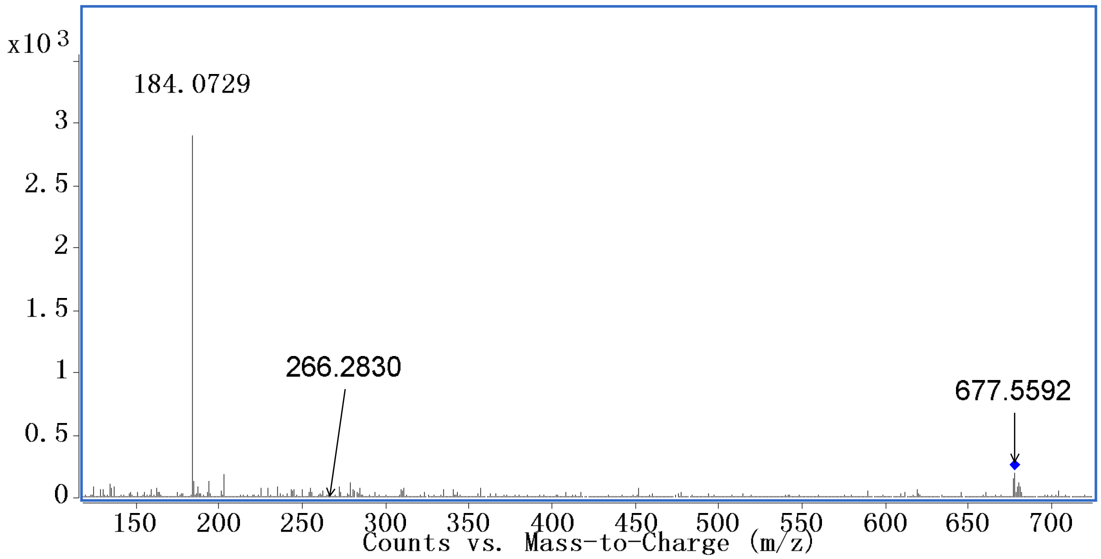

No.69 **SM (d18:0/14:0)**: $[M+H]^+$ 677.5592, [Sa (d18:0)-$2H_2O$+H]$^+$ 266.2830, [phosphocholine+H]$^+$ 184.0729, the collision energy was set as 40eV.

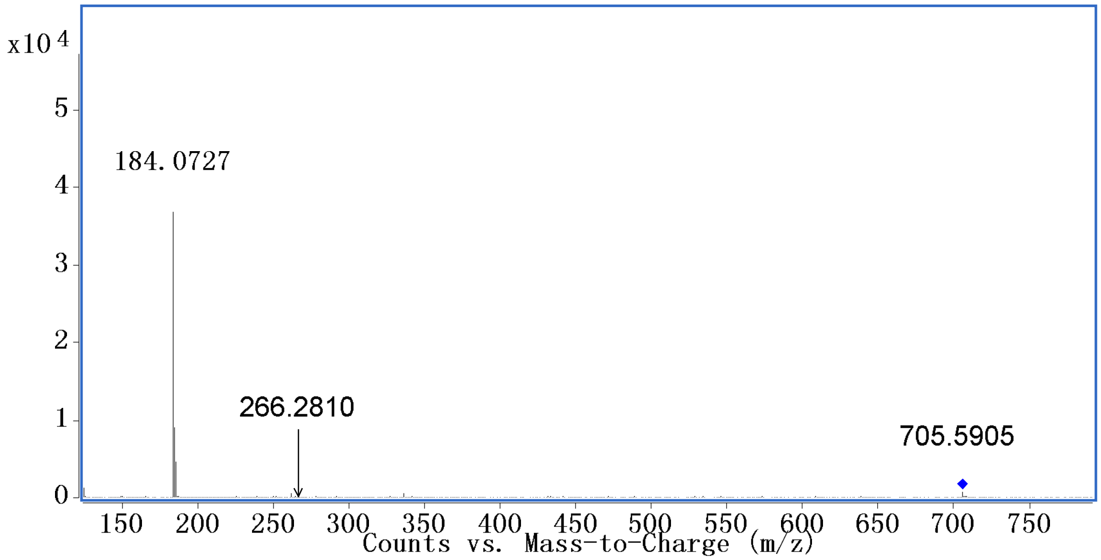

No.70 **SM (d18:0/16:0)**: $[M+H]^+$ 705.5905, $[Sa\ (d18{:}0)\text{-}2H_2O+H]^+$ 266.2810, $[\text{phosphocholine}+H]^+$ 184.0727, the collision energy was set as 40eV.

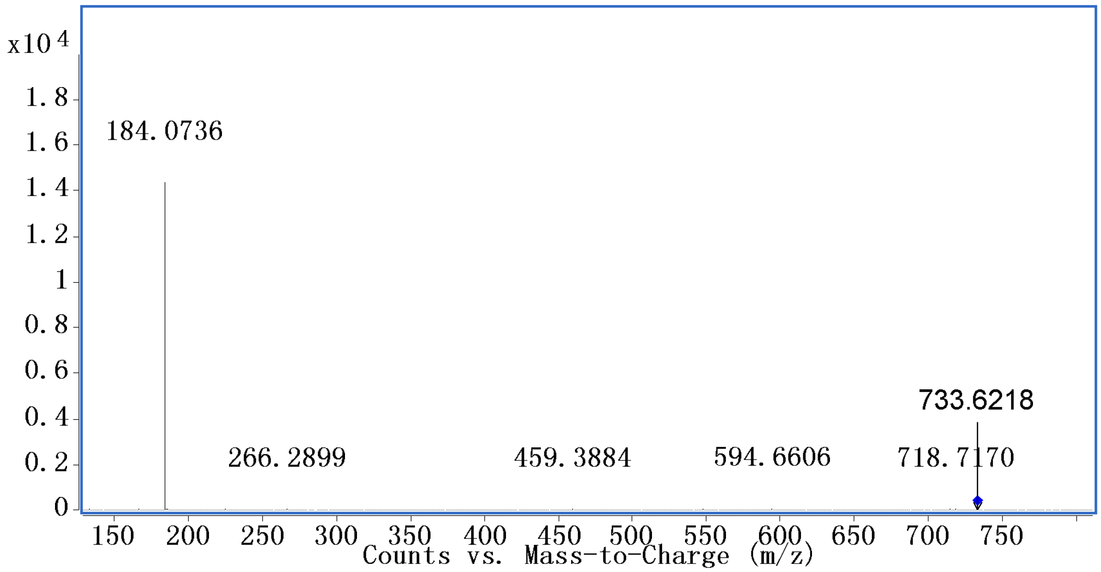

No.71 **SM (d18:0/18:0)**: $[M+H]^+$ 733.6218, $[Sa\ (d18{:}0)\text{-}2H_2O+H]^+$ 266.2899, $[\text{phosphocholine}+H]^+$ 184.0736, the collision energy was set as 40eV.

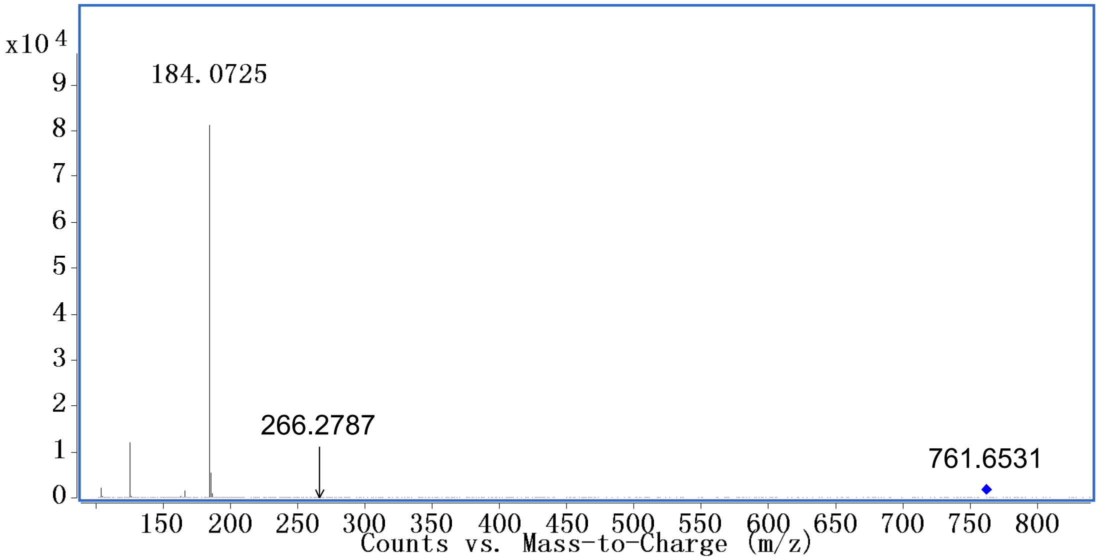

No.72 **SM (d18:0/20:0)**: $[M+H]^+$ 761.6531, [Sa (d18:0)-$2H_2O$+H]$^+$ 266.2787, [phosphocholine+H]$^+$ 184.0725, the collision energy was set as 40eV.

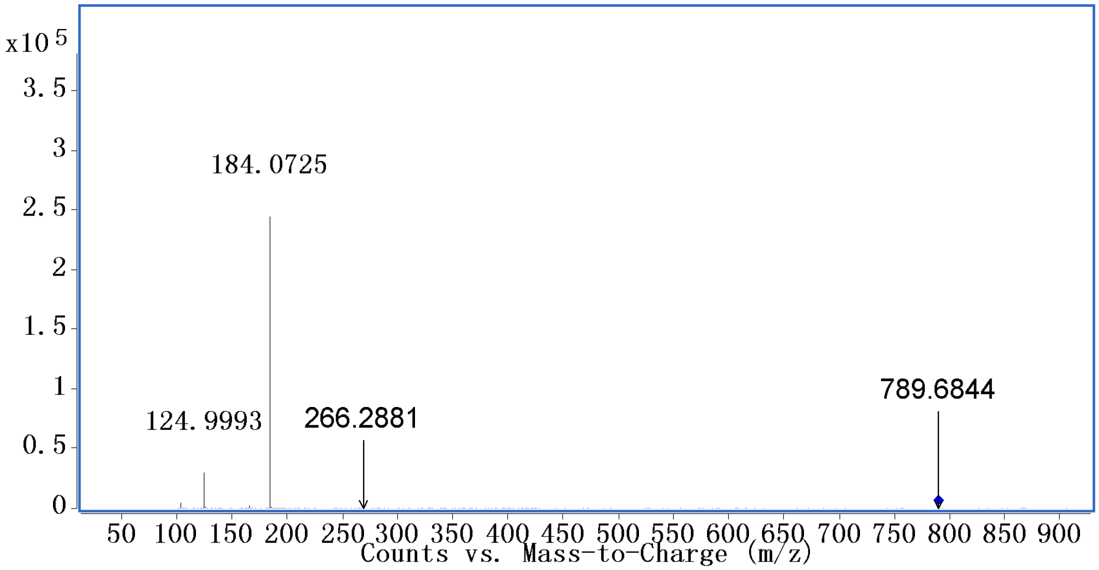

No.73 **SM (d18:0/22:0)**: $[M+H]^+$ 789.6844, [Sa (d18:0)-$2H_2O$+H]$^+$ 266.2881, [phosphocholine+H]$^+$ 184.0725, the collision energy was set as 40eV.

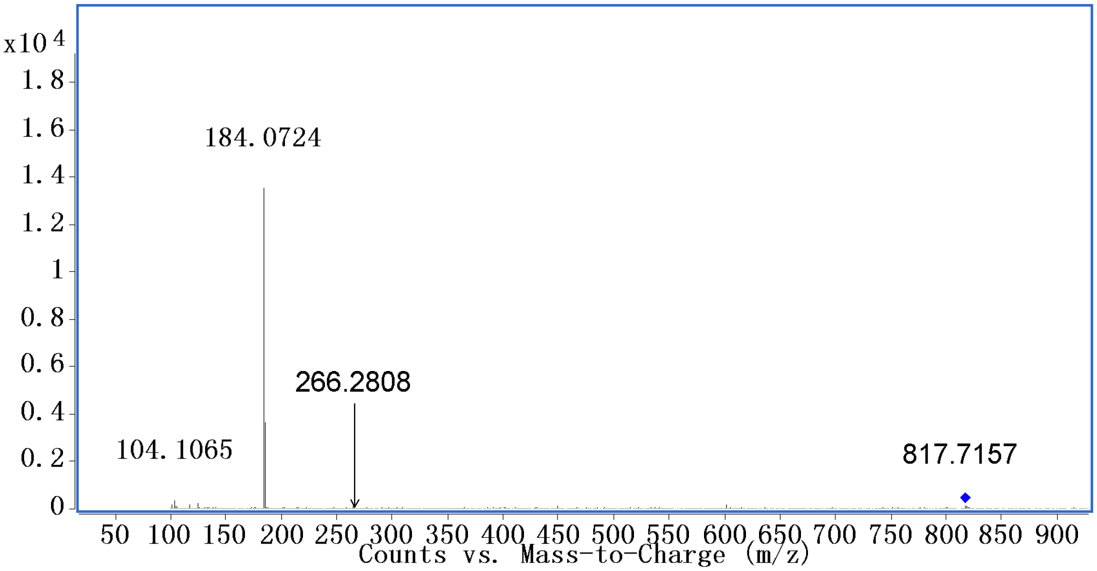

No.74 **SM (d18:0/24:0)**: $[M+H]^+$ 817.7157, $[Sa\ (d18:0)\text{-}2H_2O+H]^+$ 266.2808, $[phosphocholine+H]^+$ 184.0724, the collision energy was set as 40eV.

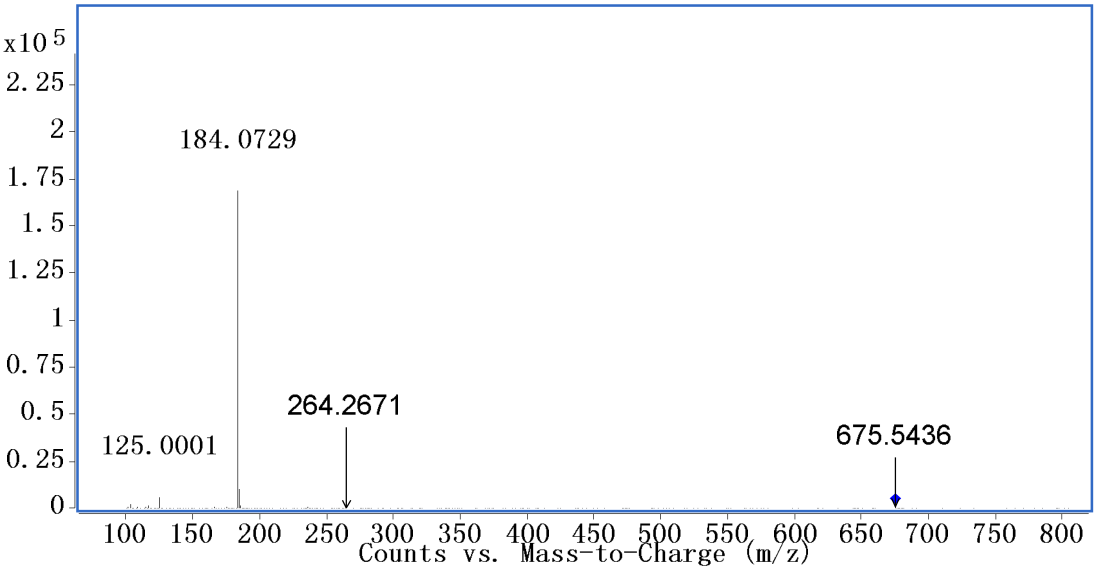

No.75 **SM (d18:1/14:0)**: $[M+H]^+$ 675.5436, $[So\ (d18:1)\text{-}2H_2O+H]^+$ 264.2671, $[phosphocholine+H]^+$ 184.0729, the collision energy was set as 40eV.

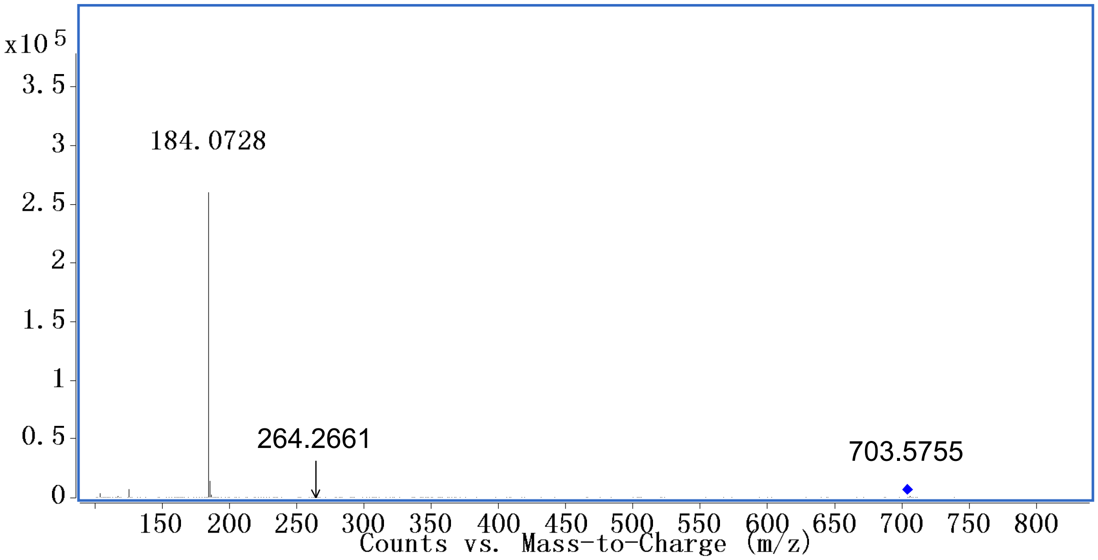

No.76 **SM (d18:1/16:0)**: $[M+H]^+$ 703.5755, [So (d18:1)-$2H_2O$+H]$^+$ 264.2661, [phosphocholine+H]$^+$ 184.078, the collision energy was set as 40eV.

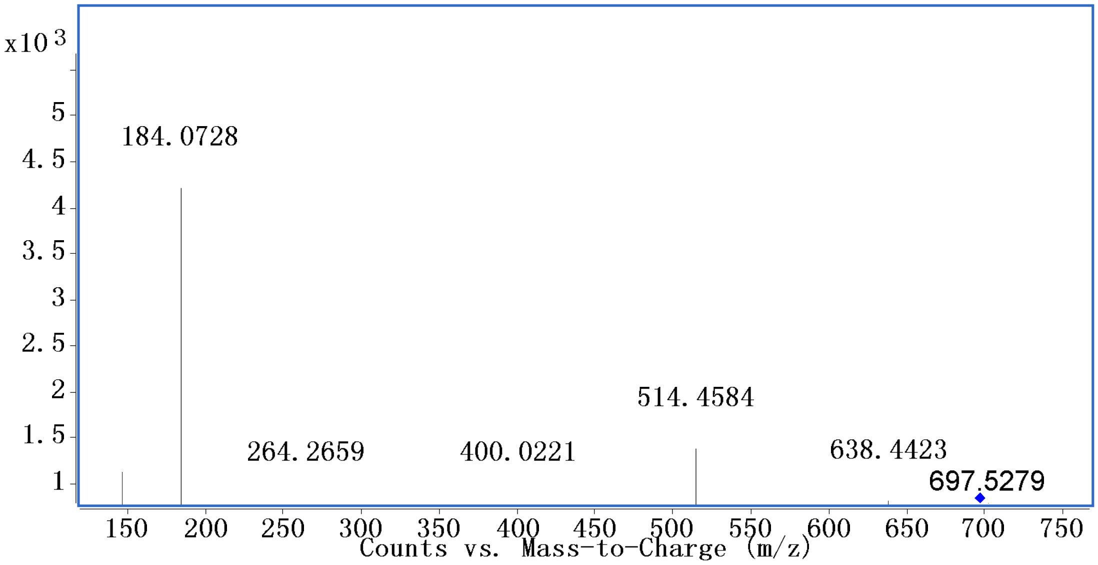

No.77 **SM (d18:1/16:3)**: $[M+H]^+$ 697.5279, [So (d18:1)-$2H_2O$+H]$^+$ 264.2659, [phosphocholine+H]$^+$ 184.0728, the collision energy was set as 40eV.

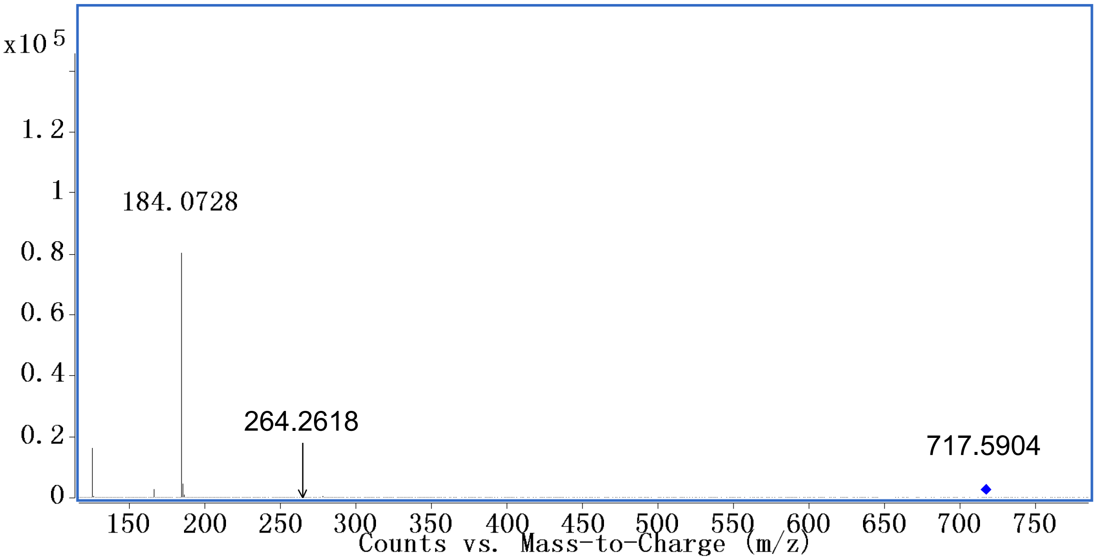

No.78 **SM (d18:1/17:0)**: $[M+H]^+$ 717.5904, [So (d18:1)-2H_2O+H]$^+$ 264.2618, [phosphocholine+H]$^+$ 184.0728, the collision energy was set as 40eV.

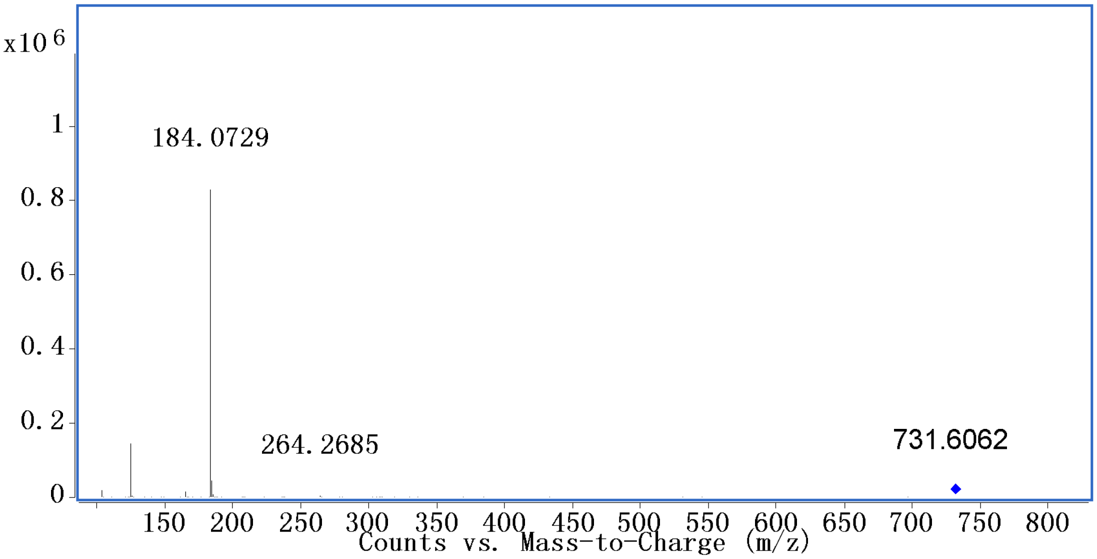

No.79 **SM (d18:1/18:0)**: $[M+H]^+$ 731.6062, [So (d18:1)-2H_2O+H]$^+$ 264.2685, [phosphocholine+H]$^+$ 184.0729, the collision energy was set as 40eV.

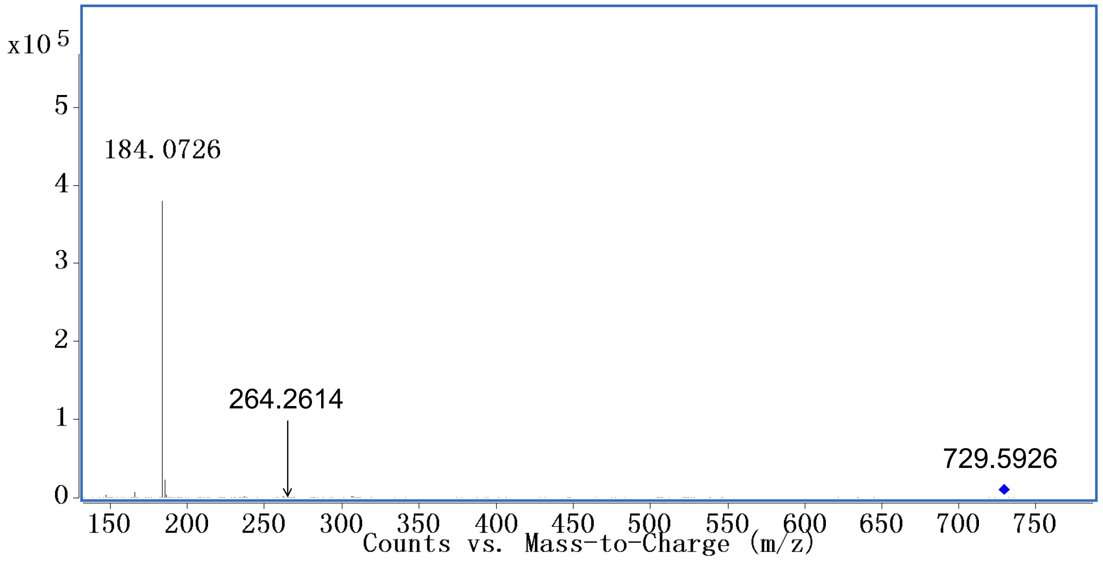

No.80 **SM (d18:1/18:1)**: $[M+H]^+$ 729.5926, $[So\ (d18:1)-2H_2O+H]^+$ 264.2614, $[phosphocholine+H]^+$ 184.0726, the collision energy was set as 40eV.

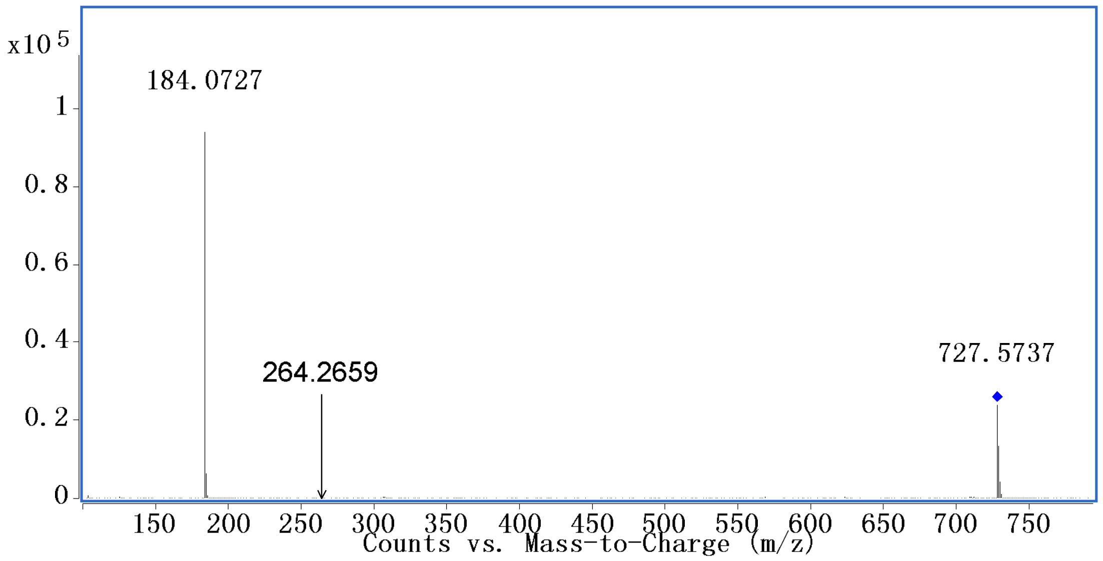

No.81 **SM (d18:1/18:2)**: $[M+H]^+$ 727.5737, $[So\ (d18:1)-2H_2O+H]^+$ 264.2659, $[phosphocholine+H]^+$ 184.0727, the collision energy was set as 40eV.

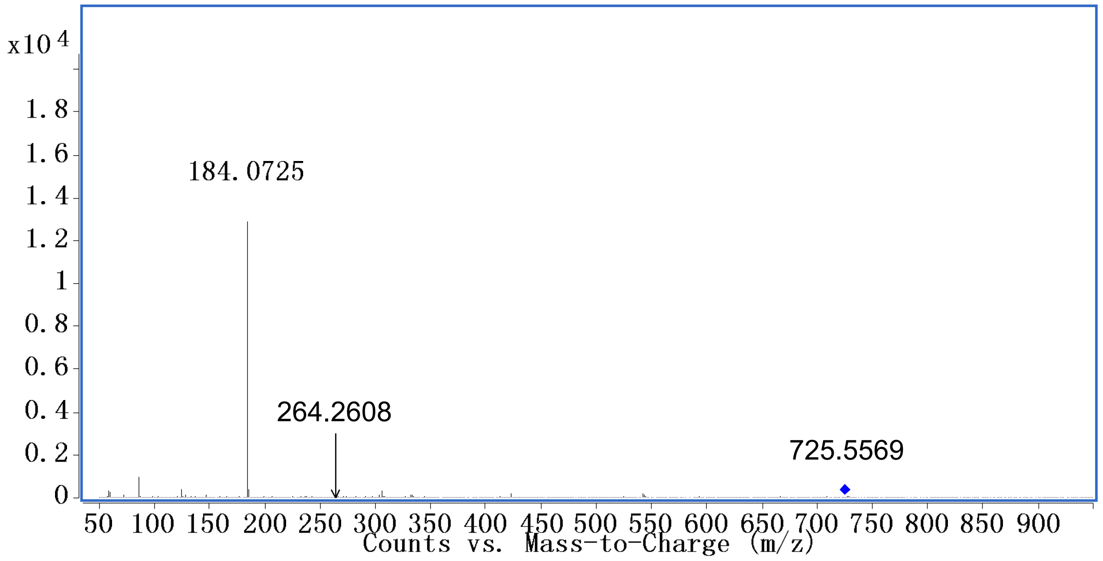

No.82 **SM (d18:1/18:3)**: $[M+H]^+$ 725.5569, [So (d18:1)-$2H_2O+H]^+$ 264.2608, $[\text{phosphocholine}+H]^+$ 184.0725, the collision energy was set as 40eV.

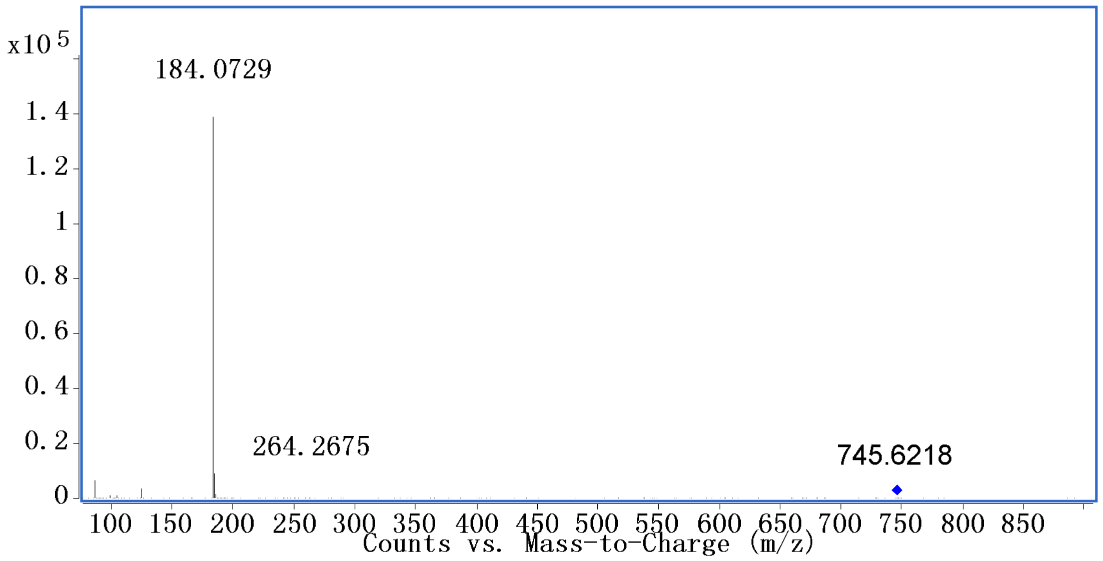

No.83 **SM (d18:1/19:0)**: $[M+H]^+$ 745.6218, [So (d18:1)-$2H_2O+H]^+$ 264.2675, $[\text{phosphocholine}+H]^+$ 184.079, the collision energy was set as 40eV.

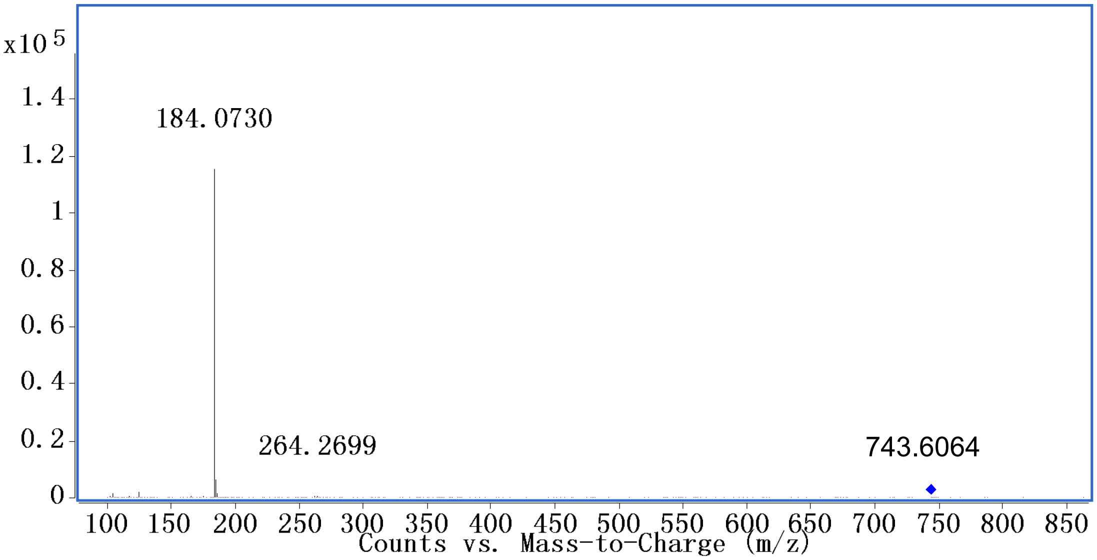

No.84 **SM (d18:1/19:1)**: $[M+H]^+$ 743.6064, $[So (d18:1)-2H_2O+H]^+$ 264.2699, $[phosphocholine+H]^+$ 184.0730, the collision energy was set as 40eV.

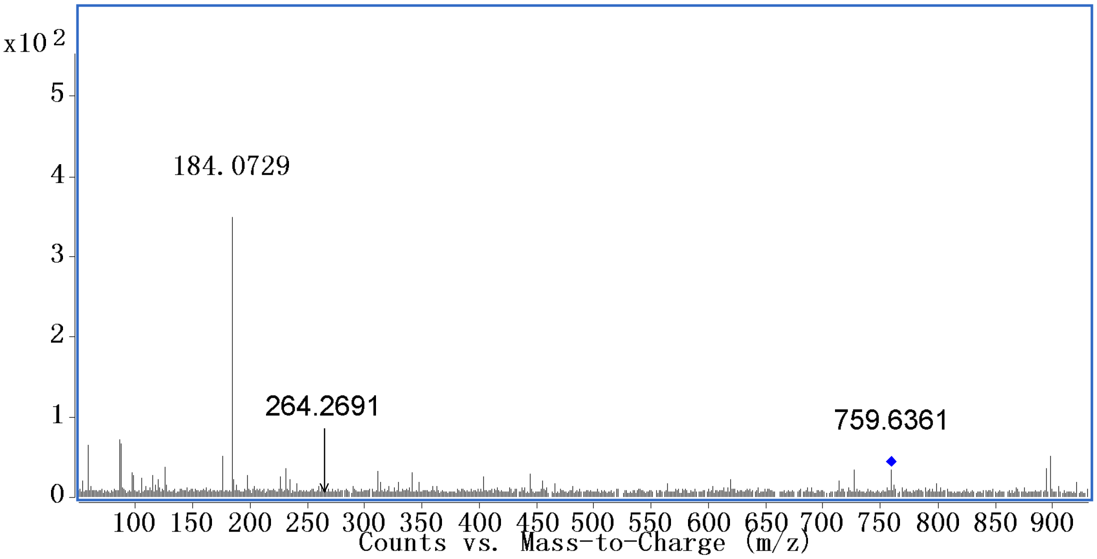

No.85 **SM (d18:1/20:0)**: $[M+H]^+$ 759.6361, $[So (d18:1)-2H_2O+H]^+$ 264.2691, $[phosphocholine+H]^+$ 184.0729, the collision energy was set as 40eV.

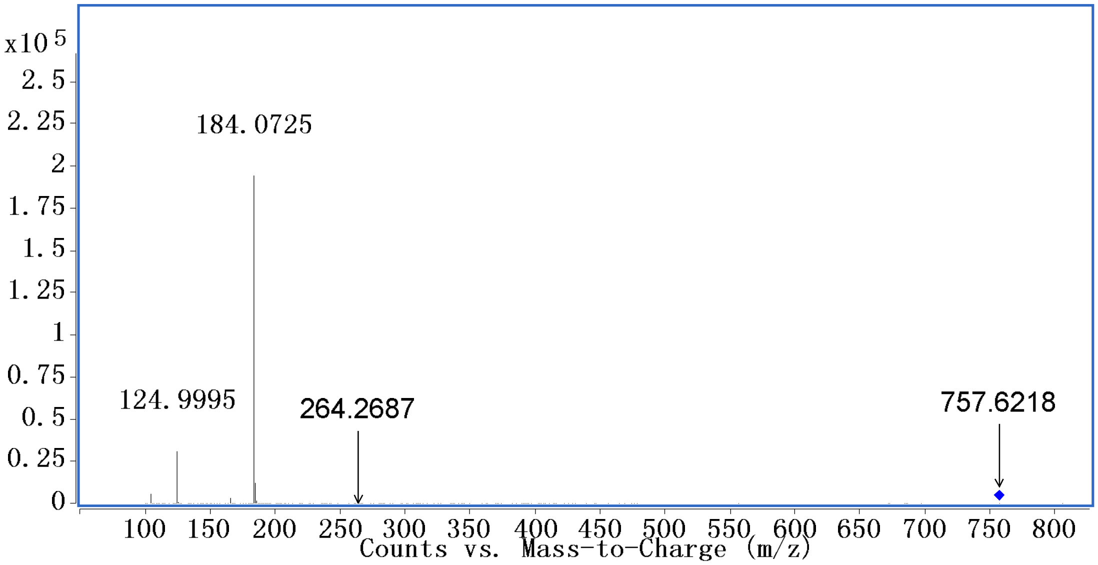

No.86 **SM (d18:1/20:1)**: $[M+H]^+$ 757.6218, [So (d18:1)-$2H_2O$+H]$^+$ 264.2687, [phosphocholine+H]$^+$ 184.0725, the collision energy was set as 40eV.

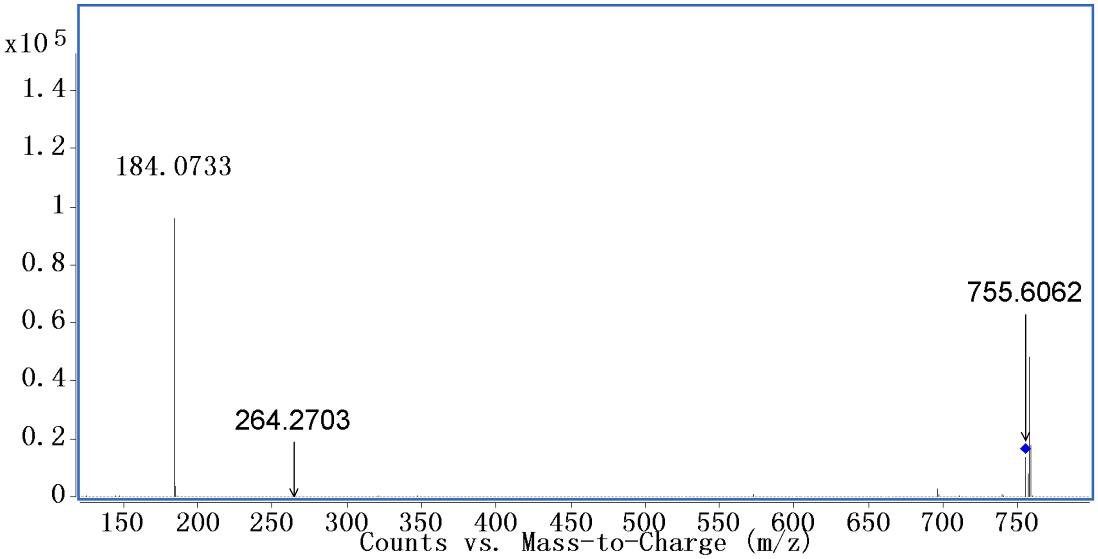

No.87 **SM (d18:1/20:2)**: $[M+H]^+$ 755.6062, [So (d18:1)-$2H_2O$+H]$^+$ 264.2703, [phosphocholine+H]$^+$ 184.0733, the collision energy was set as 20eV.

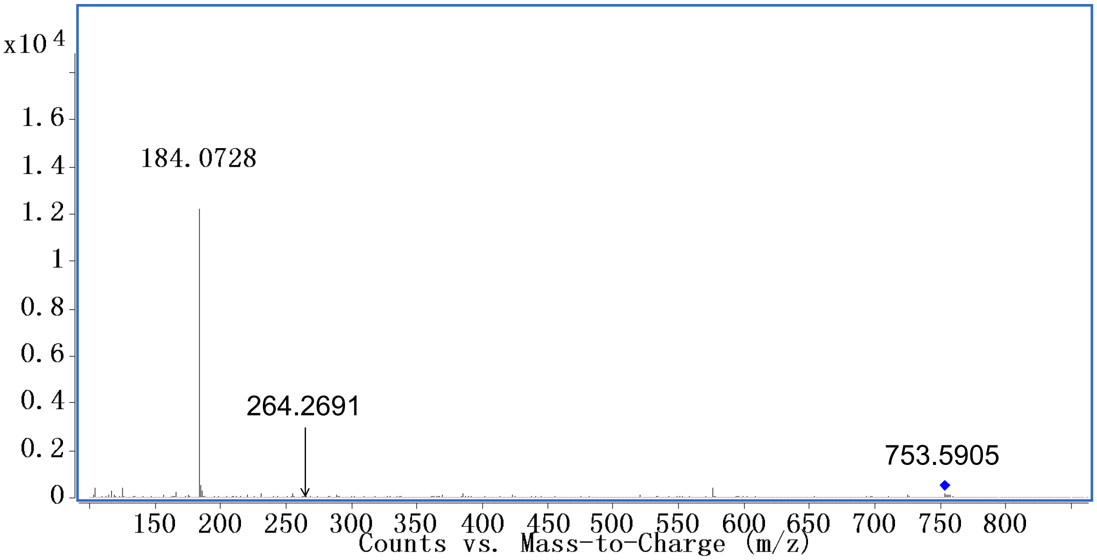

No.88 **SM (d18:1/20:3)**: $[M+H]^+$ 753.5905, [So (d18:1)-2H_2O+H]$^+$ 264.2691, [phosphocholine+H]$^+$ 184.0728, the collision energy was set as 40eV.

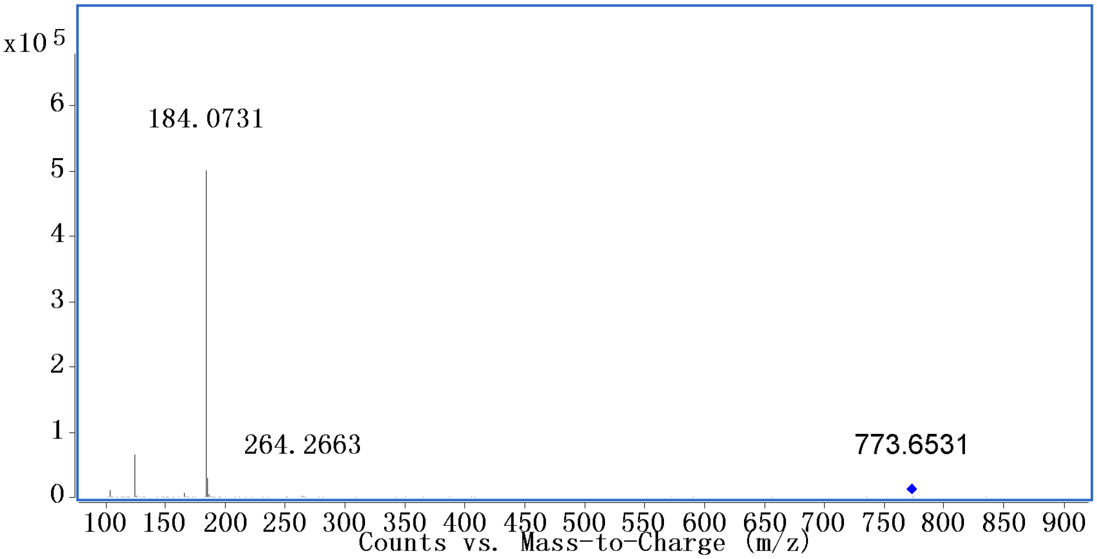

No.89 **SM (d18:1/21:0)**: $[M+H]^+$ 773.6531, [So (d18:1)-2H_2O+H]$^+$ 264.2663, [phosphocholine+H]$^+$ 184.0731, the collision energy was set as 40eV.

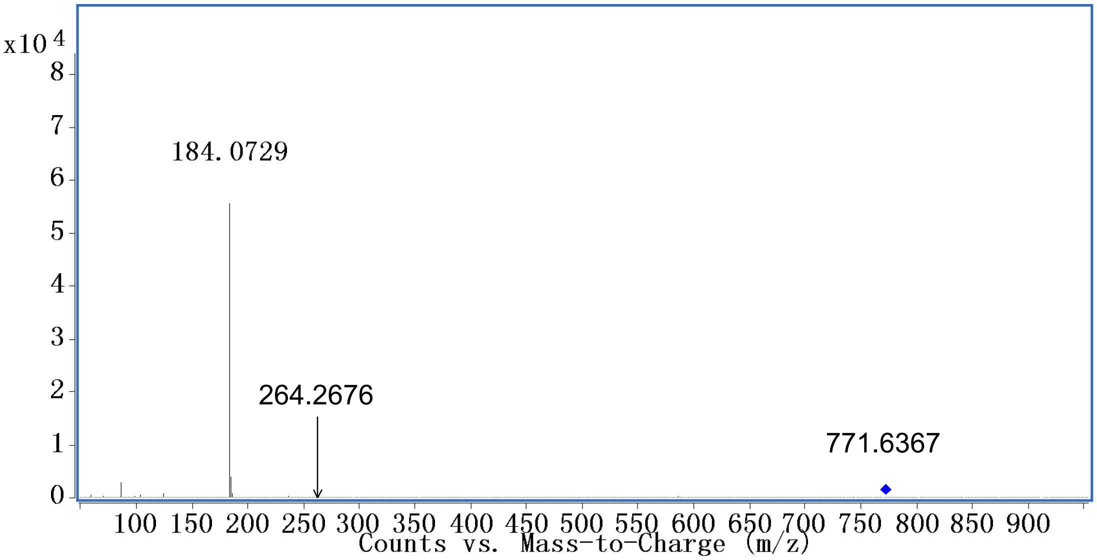

No.90 **SM (d18:1/21:1)**: $[M+H]^+$ 771.6367, [So (d18:1)-$2H_2O$+H]$^+$ 264.2676, [phosphocholine+H]$^+$ 184.0729, the collision energy was set as 40eV.

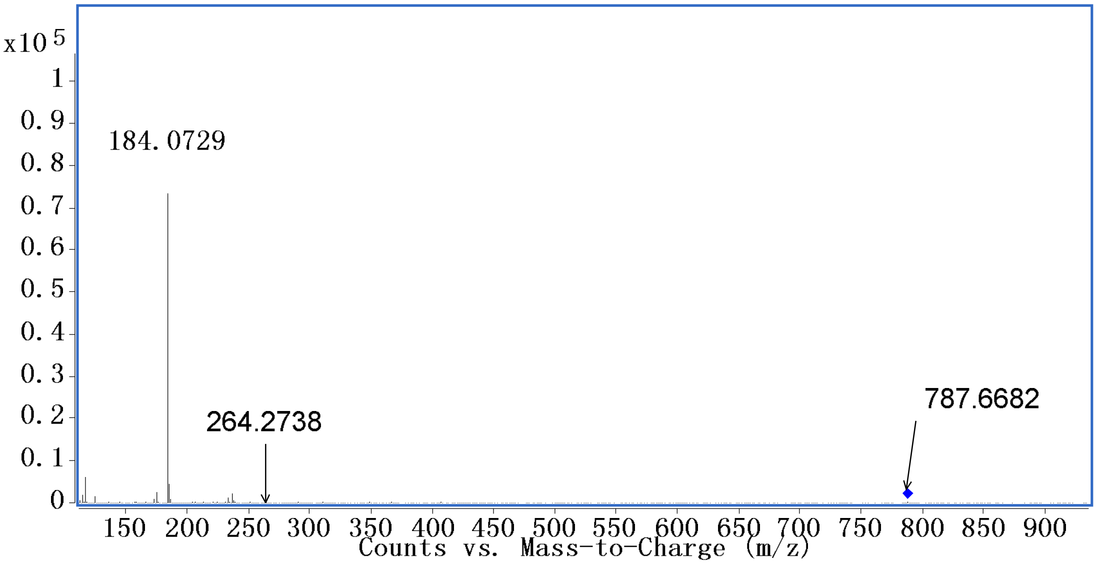

No.91 **SM (d18:1/22:0)**: $[M+H]^+$ 787.6682, [So (d18:1)-$2H_2O$+H]$^+$ 264.2738, [phosphocholine+H]$^+$ 184.0729, the collision energy was set as 40eV.

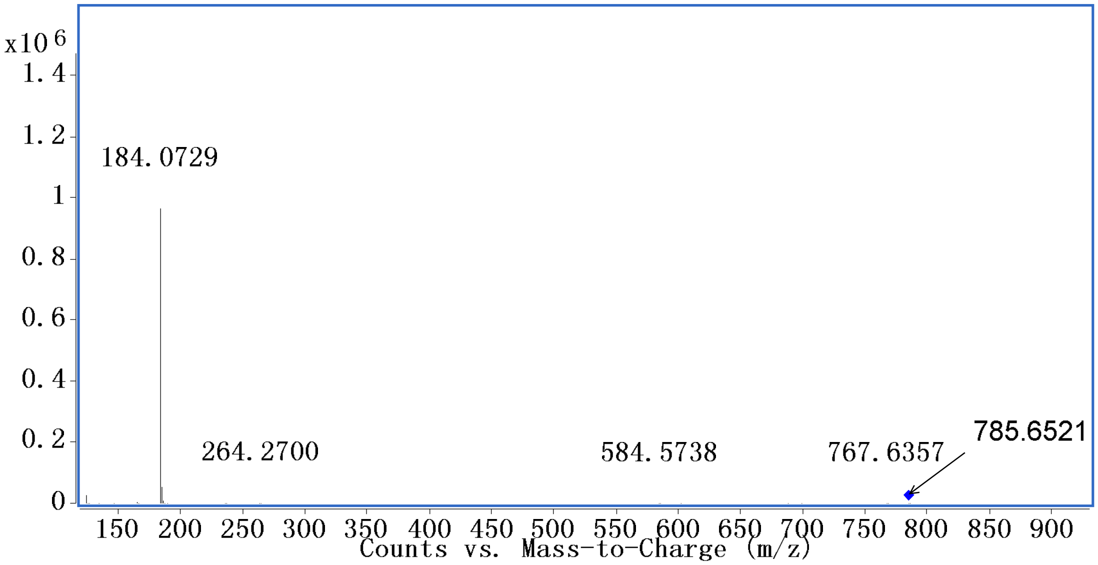

No.92 **SM (d18:1/22:1)**: $[M+H]^+$ 785.6521, $[So (d18:1)-2H_2O+H]^+$ 264.2700, $[phosphocholine+H]^+$ 184.0729, the collision energy was set as 40eV.

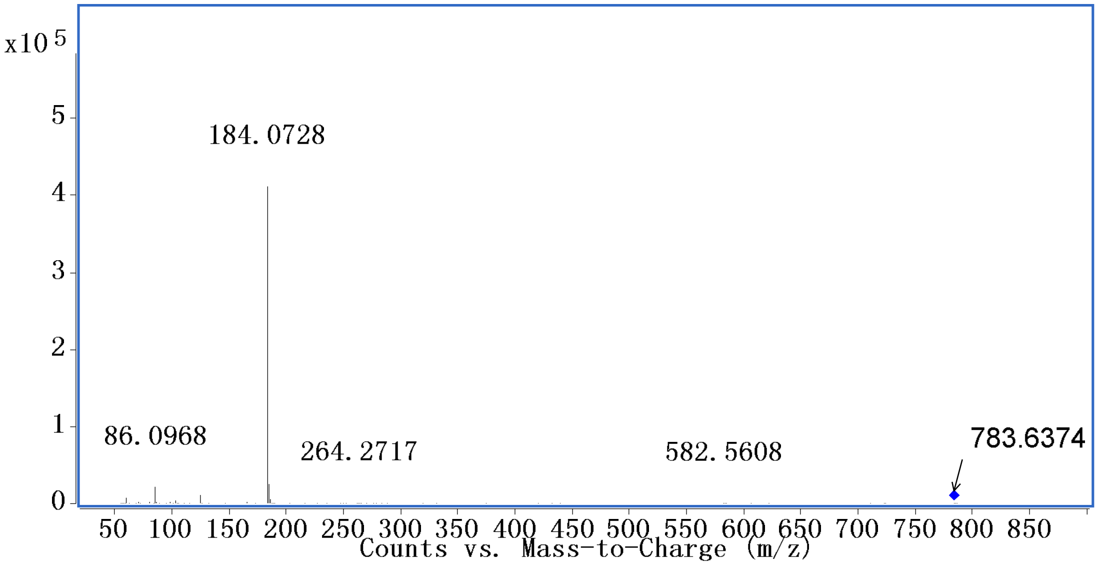

No.93 **SM (d18:1/22:2)**: $[M+H]^+$ 783.6374, $[So (d18:1)-2H_2O+H]^+$ 264.2717, $[phosphocholine+H]^+$ 184.0728, the collision energy was set as 40eV.

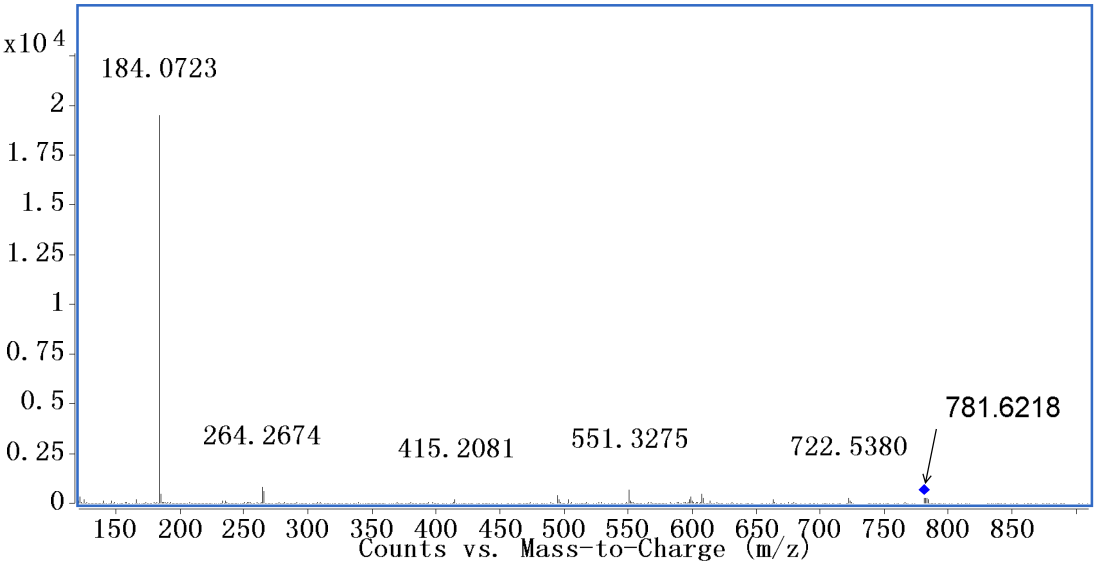

No.94 **SM (d18:1/22:3)**: $[M+H]^+$ 781.6218, [So (d18:1)-$2H_2O$+H]$^+$ 264.2674, [phosphocholine+H]$^+$ 184.0723, the collision energy was set as 40eV.

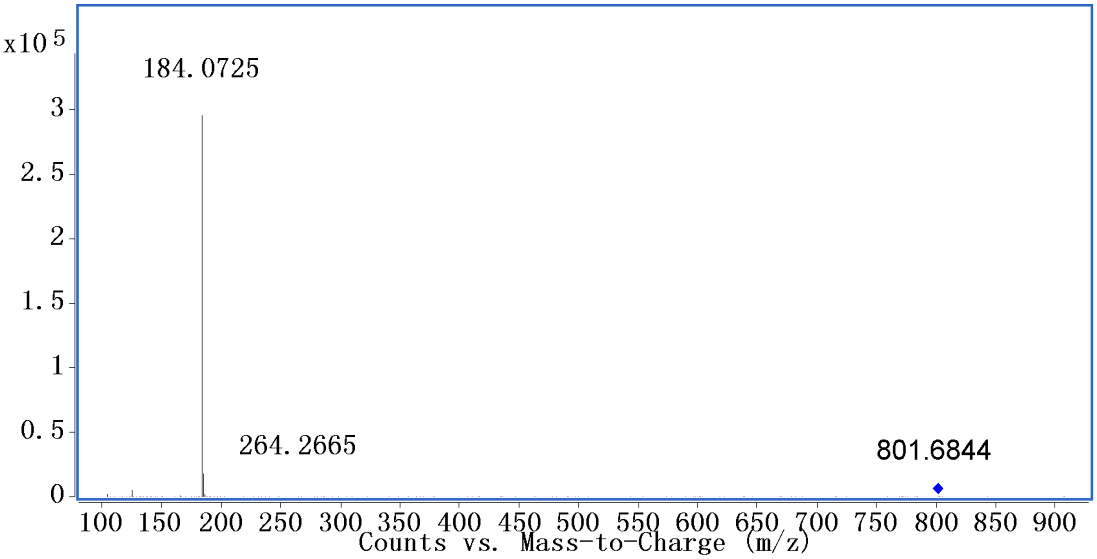

No.95 **SM (d18:1/23:0)**: $[M+H]^+$ 801.6844, [So (d18:1)-$2H_2O$+H]$^+$ 264.2665, [phosphocholine+H]$^+$ 184.0725, the collision energy was set as 40eV.

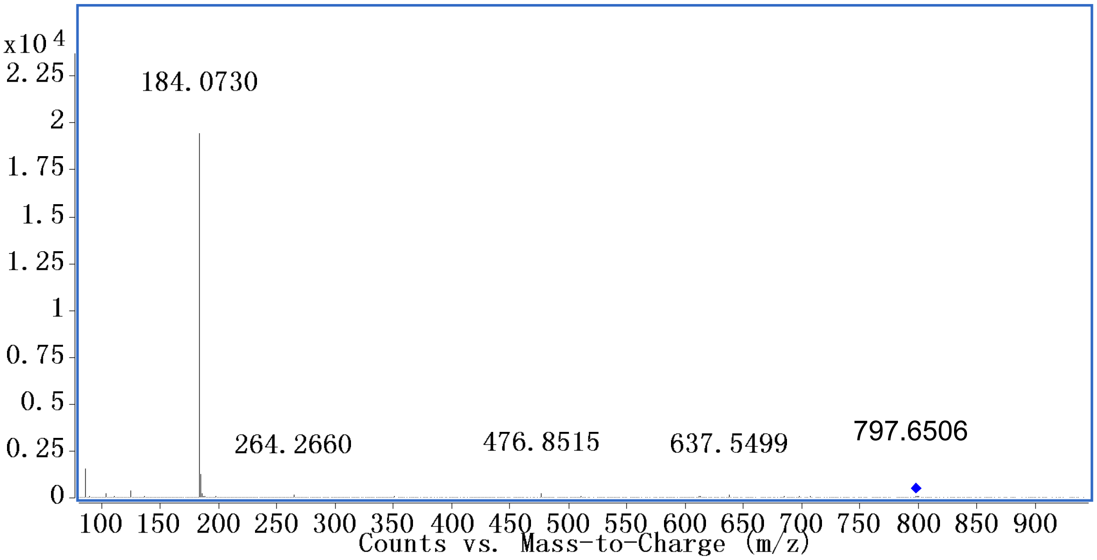

No.96 **SM (d18:1/23:2)**: $[M+H]^+$ 797.6506, [So (d18:1)-2H_2O+H]$^+$ 264.2660, [phosphocholine+H]$^+$ 184.0730, the collision energy was set as 40eV.

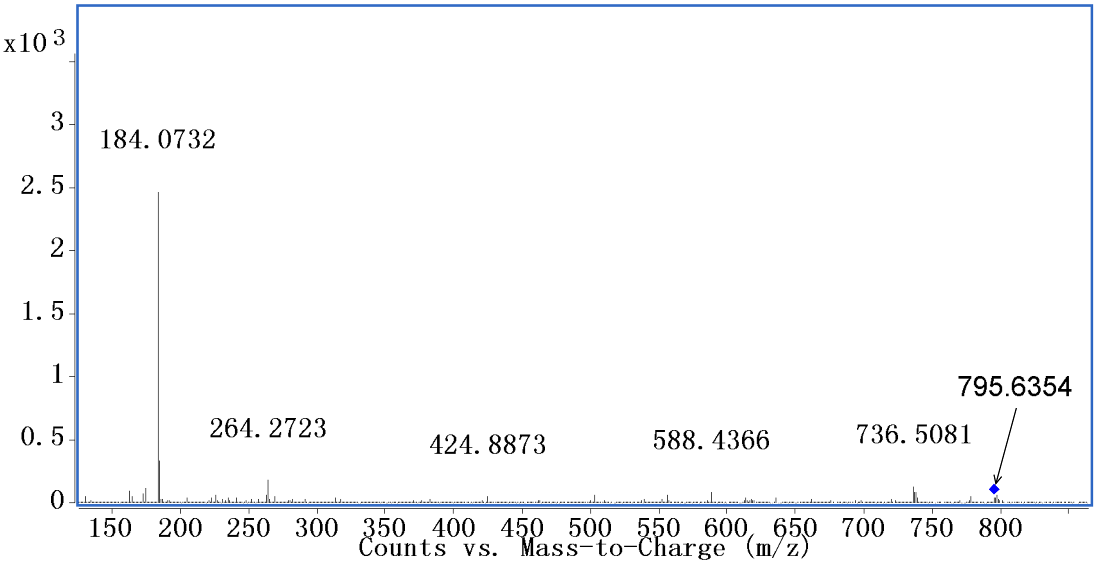

No.97 **SM (d18:1/23:3)**: $[M+H]^+$ 795.6354, [So (d18:1)-2H_2O+H]$^+$ 264.2723, [phosphocholine+H]$^+$ 184.0732, the collision energy was set as 40eV.

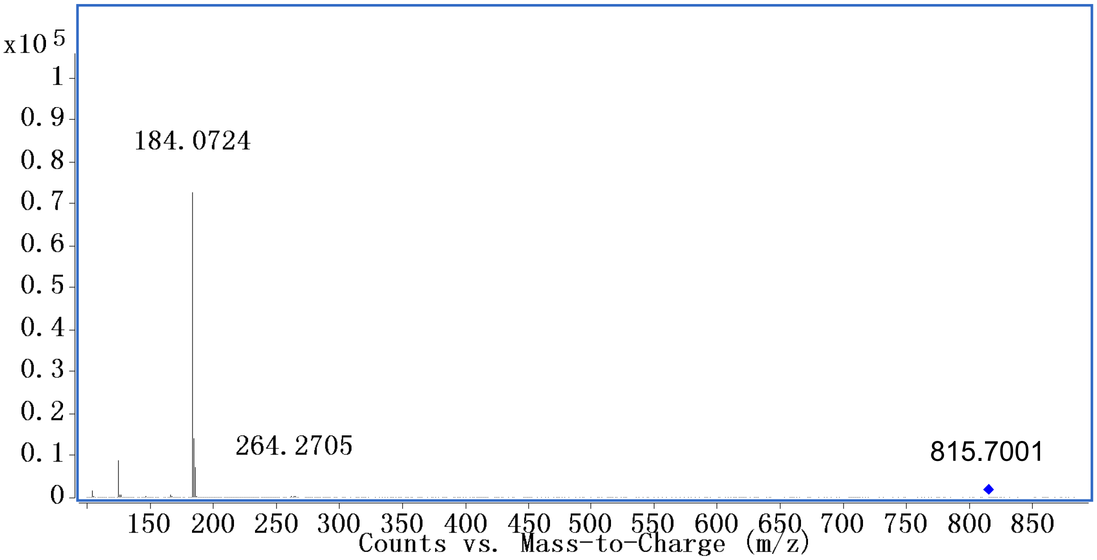

No.98 **SM (d18:1/24:0)**: $[M+H]^+$ 815.7001, [So (d18:1)-2H_2O+H]$^+$ 264.2705, [phosphocholine+H]$^+$ 184.0724, the collision energy was set as 40eV.

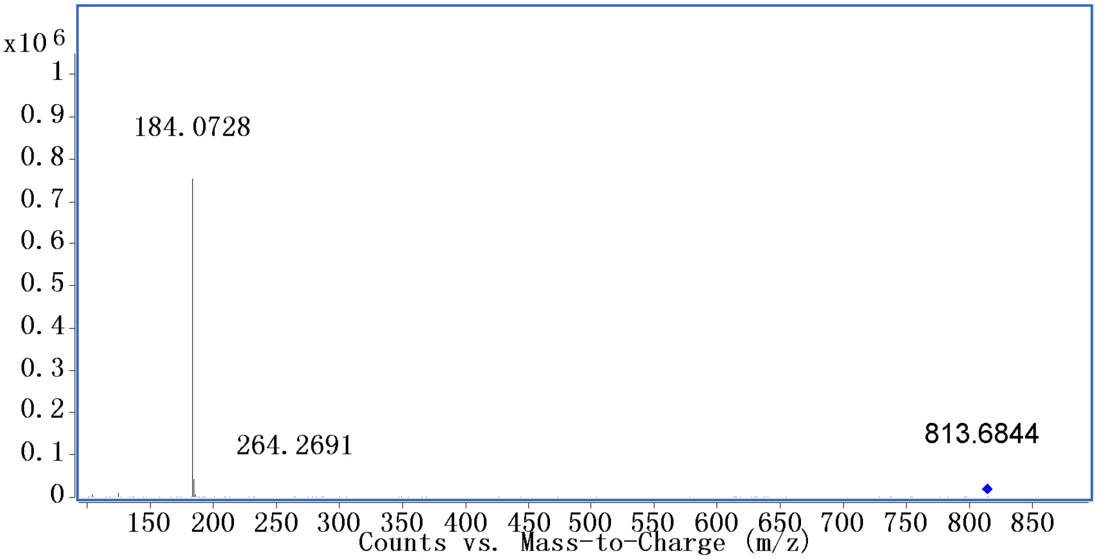

No.99 **SM (d18:1/24:1)**: $[M+H]^+$ 813.6844, [So (d18:1)-2H_2O+H]$^+$ 264.2691, [phosphocholine+H]$^+$ 184.0728, the collision energy was set as 40eV.

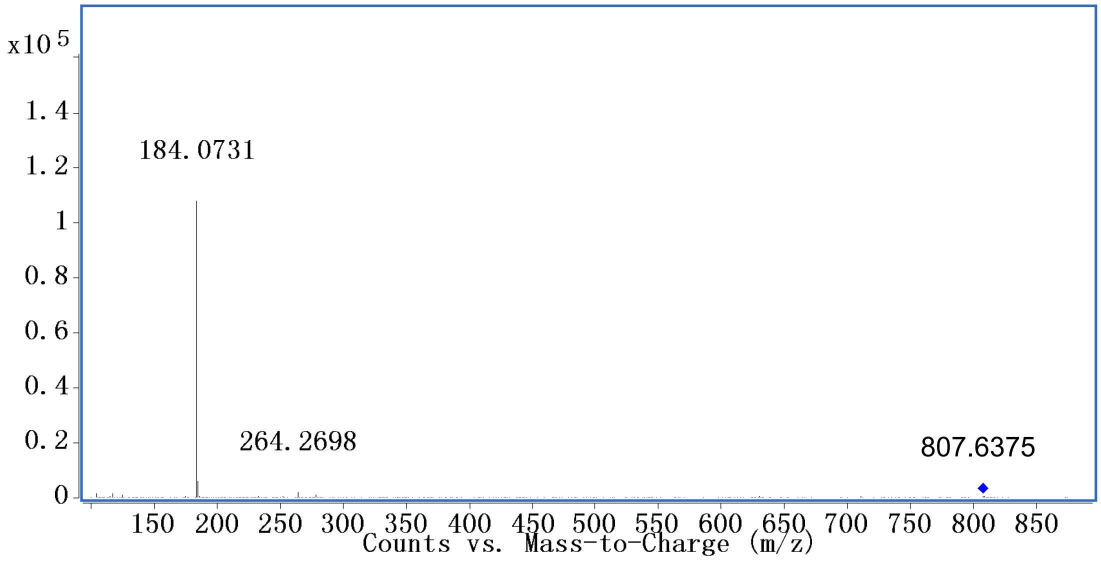

No.100 **SM (d18:1/24:4)**: $[M+H]^+$ 807.6375, [So (d18:1)-$2H_2O$+H]$^+$ 264.2698, [phosphocholine+H]$^+$ 184.0731, the collision energy was set as 40eV.

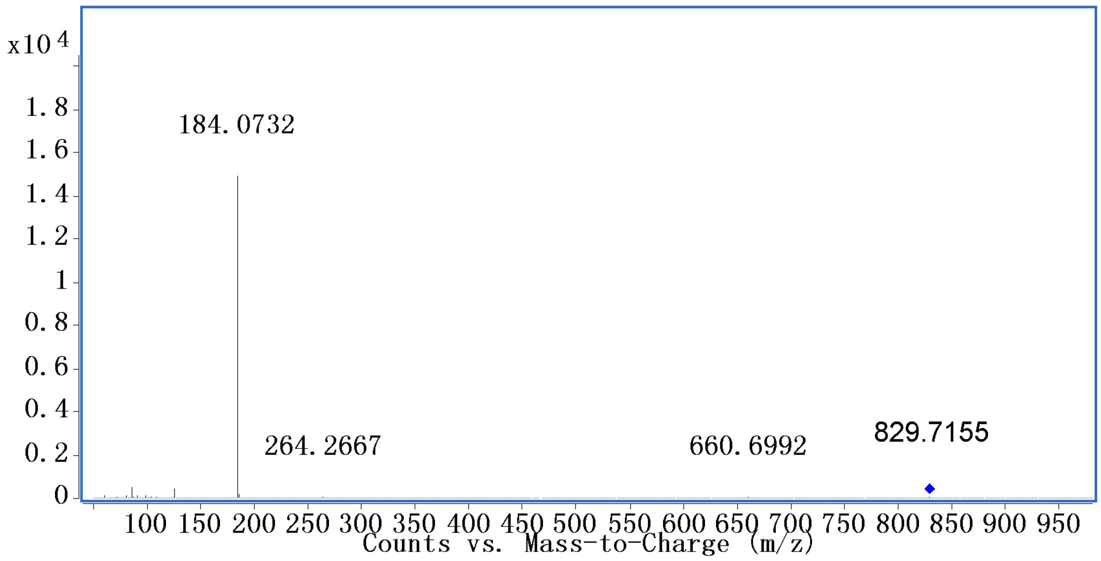

No.101 **SM (d18:1/25:0):** $[M+H]^+$ 829.7155, [So (d18:1)-$2H_2O$+H]$^+$ 264.2667, [phosphocholine+H]$^+$ 184.0732, the collision energy was set as 40eV.

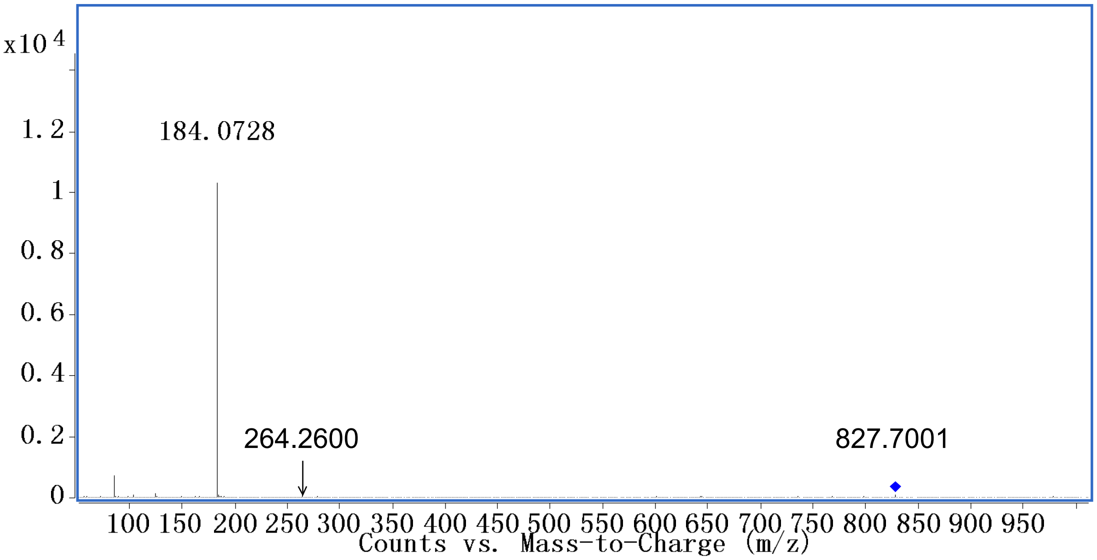

No.102 **SM (d18:1/25:1)**: $[M+H]^+$ 827.7001, [So (d18:1)-$2H_2O$+H]$^+$ 264.2600, [phosphocholine+H]$^+$ 184.0728, the collision energy was set as 40eV.

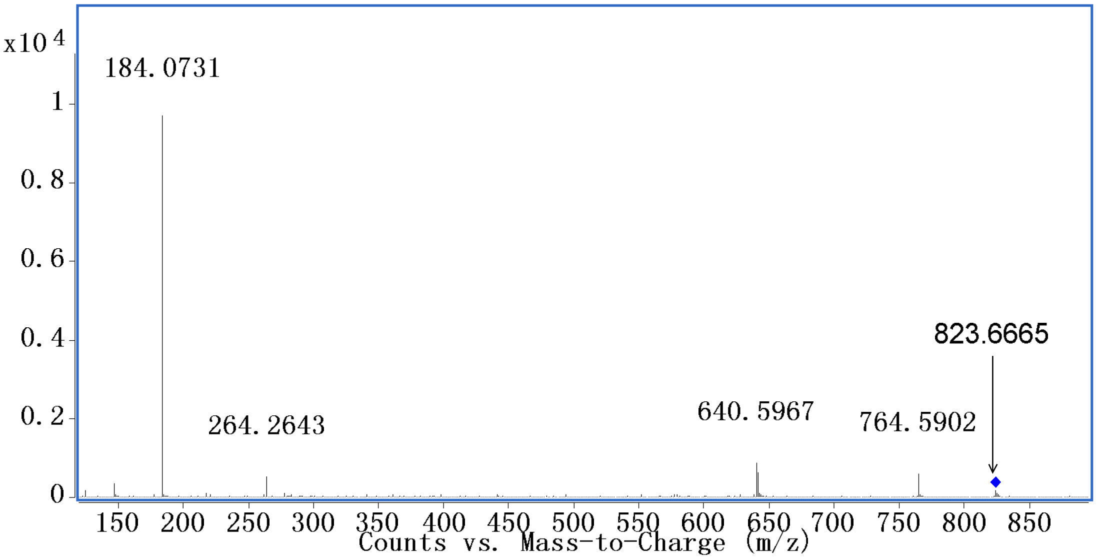

No.103 **SM (d18:1/25:3)**: $[M+H]^+$ 823.6665, [So (d18:1)-$2H_2O$+H]$^+$ 264.2643, [phosphocholine+H]$^+$ 184.0731, the collision energy was set as 40eV.

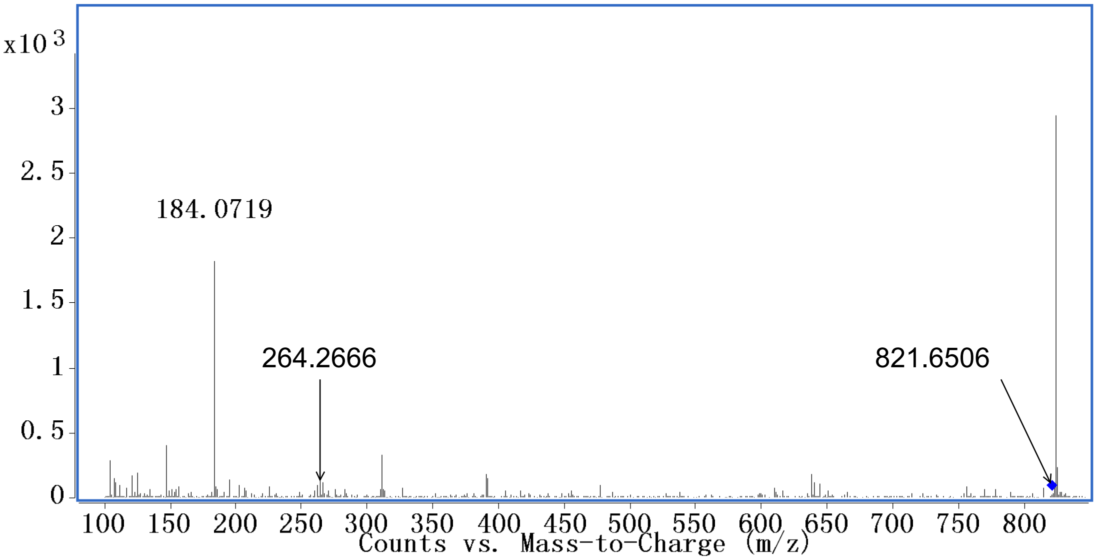

No.104 **SM (d18:1/25:4)**: $[M+H]^+$ 821.6506, [So (d18:1)-$2H_2O$+H]$^+$ 264.2666, [phosphocholine+H]$^+$ 184.0719, the collision energy was set as 40eV.

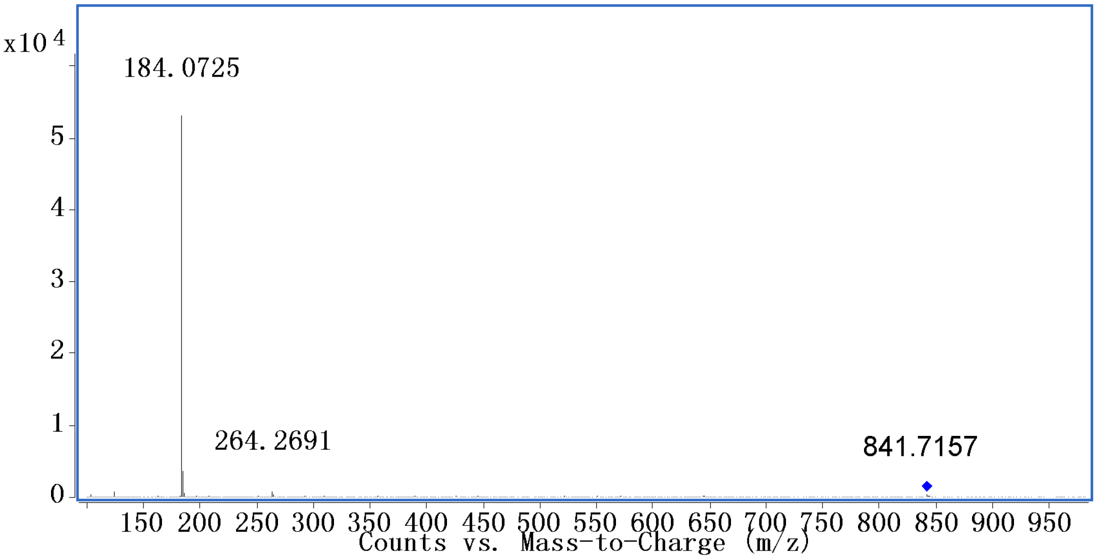

No.105 **SM (d18:1/26:1)**: $[M+H]^+$ 841.7157, [So (d18:1)-$2H_2O$+H]$^+$ 264.2691, [phosphocholine+H]$^+$ 184.0725, the collision energy was set as 40eV.

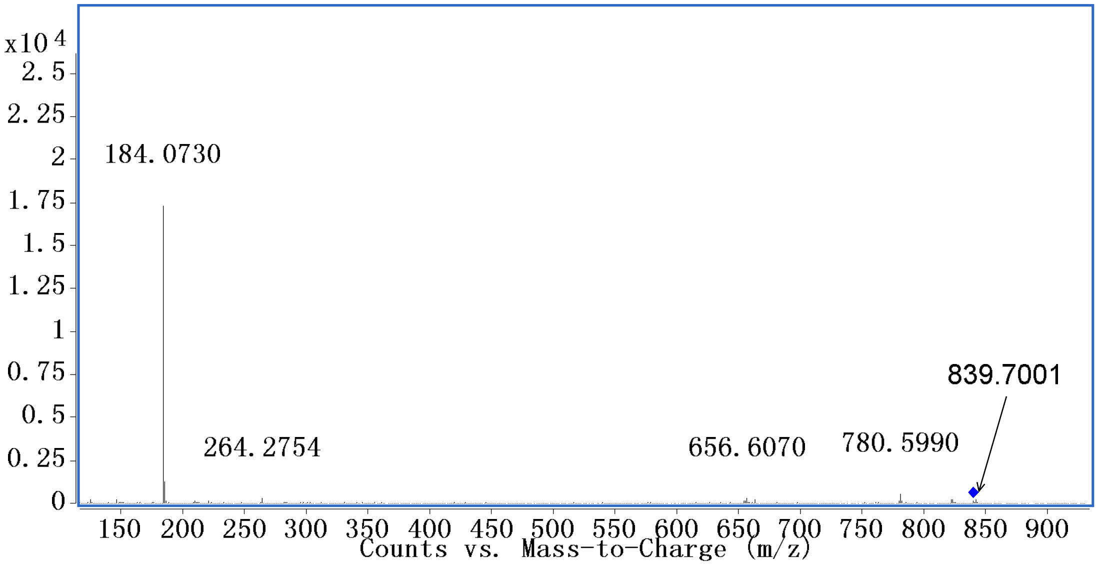

No.106 **SM (d18:1/26:2)**: $[M+H]^+$ 839.7001, [So (d18:1)-$2H_2O$+H]$^+$ 264.2754, [phosphocholine+H]$^+$ 184.0730, the collision energy was set as 40eV.

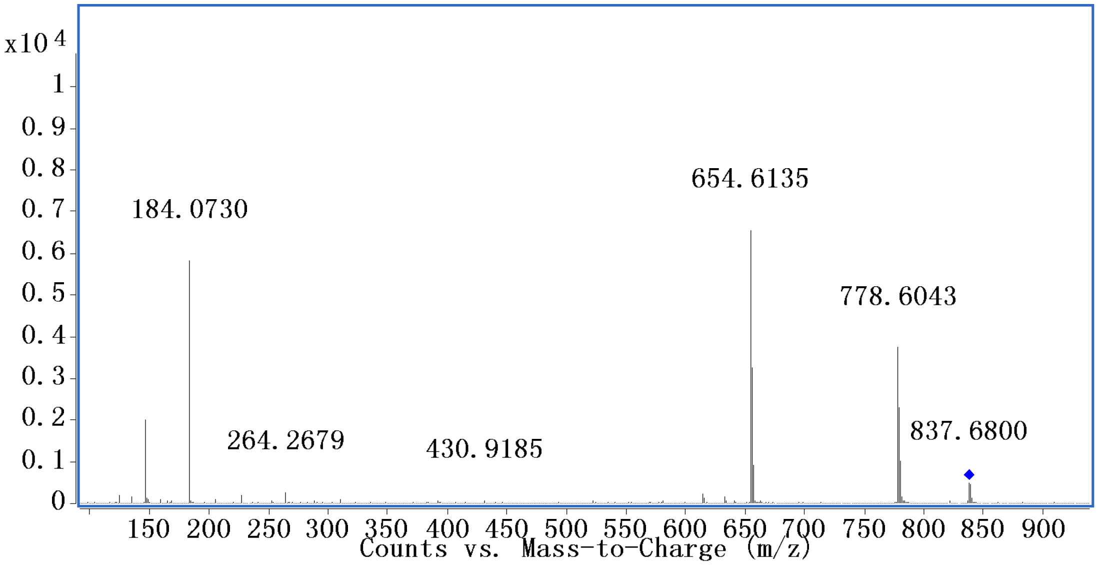

No.107 **SM (d18:1/26:3)**: $[M+H]^+$ 837.6800, [So (d18:1)-$2H_2O$+H]$^+$ 264.2679, [phosphocholine+H]$^+$ 184.0730, the collision energy was set as 40eV.

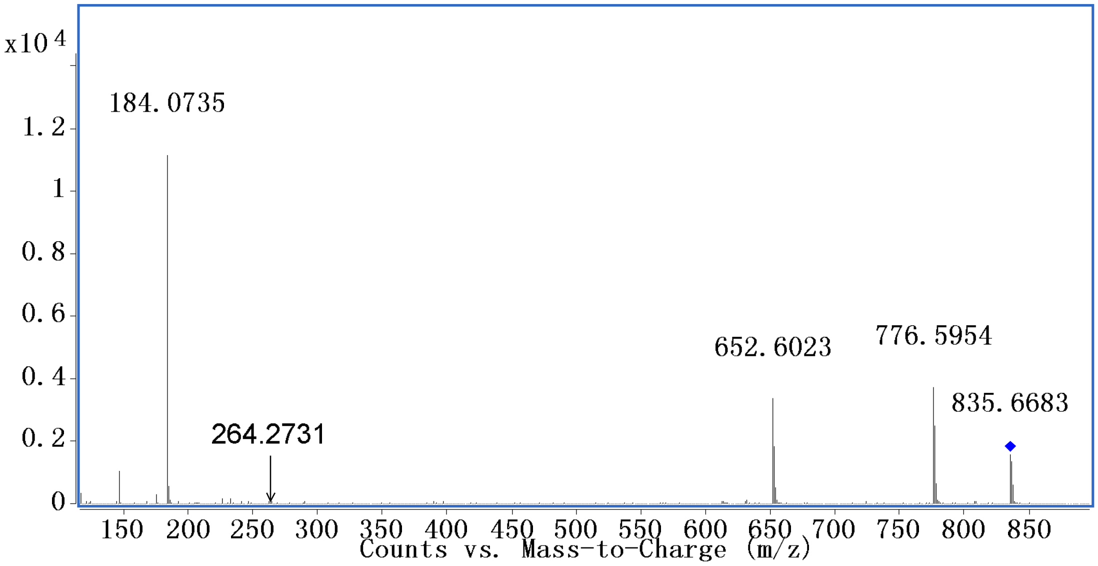

No.108 **SM (d18:1/26:4)**: $[M+H]^+$ 835.6683, [So (d18:1)-$2H_2O$+H]$^+$ 264.2731, [phosphocholine+H]$^+$ 184.0735, the collision energy was set as 40eV.

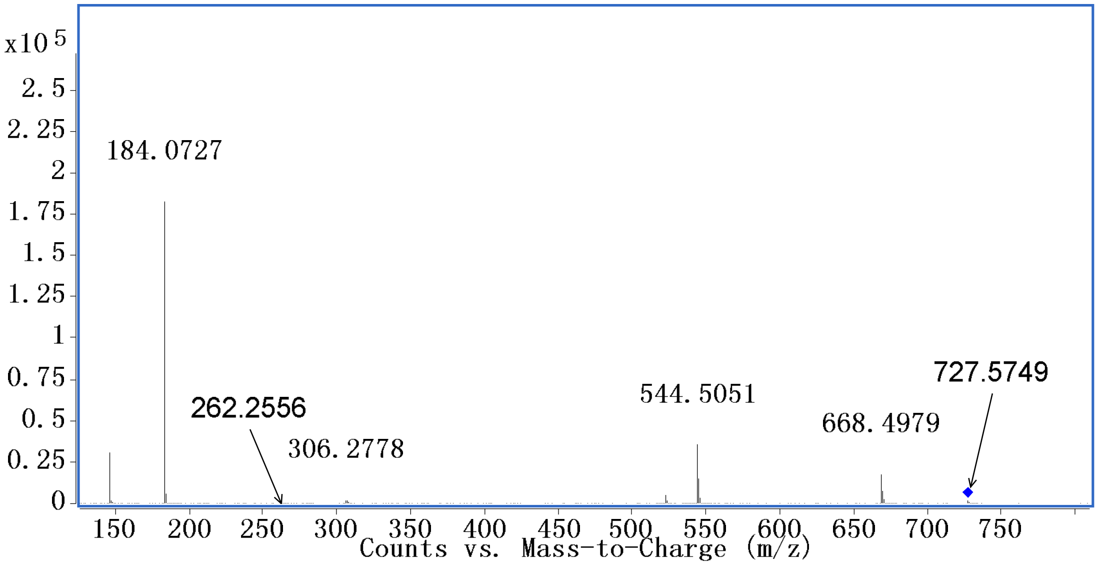

No.109 **SM (d18:2/18:1)**: $[M+H]^+$ 727.5749, [So (d18:2)-$2H_2O$+H]$^+$ 262.2556, [phosphocholine+H]$^+$ 184.0727, the collision energy was set as 40eV.

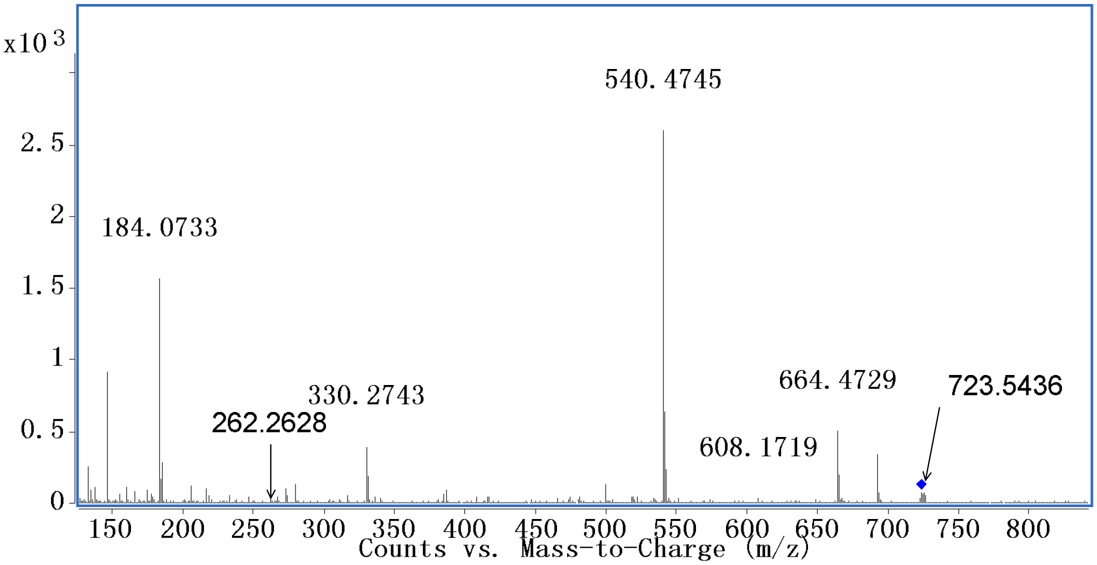

No.110 **SM (d18:2/18:3)**: $[M+H]^+$ 723.5436, [So (d18:2)-2H_2O+H]$^+$ 262.2628, [phosphocholine+H]$^+$ 184.0733, the collision energy was set as 40eV.

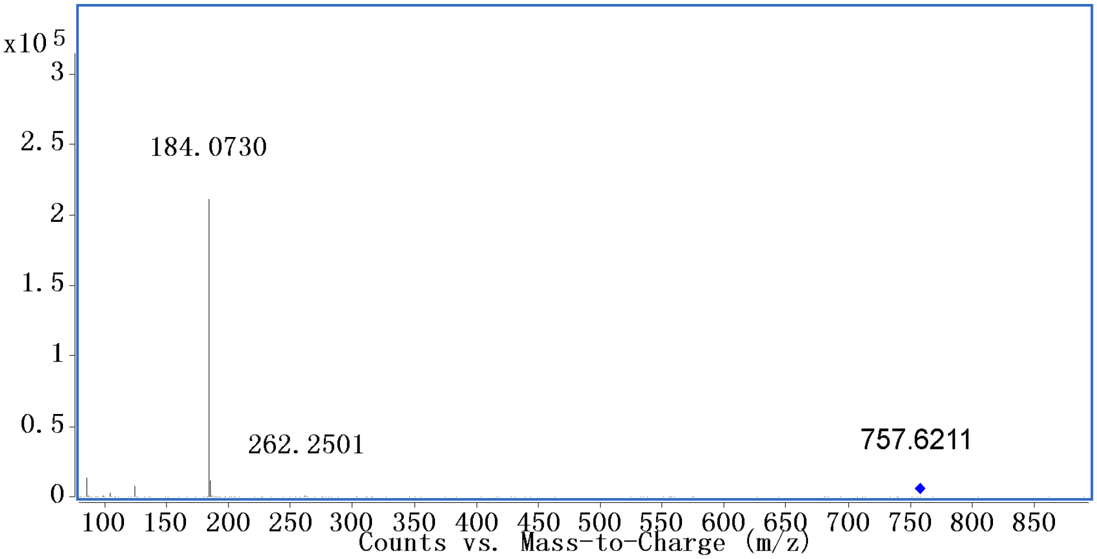

No.111 **SM (d18:2/20:0)**: $[M+H]^+$ 757.6211, [So (d18:2)-2H_2O+H]$^+$ 262.2501, [phosphocholine+H]$^+$ 184.0730, the collision energy was set as 40eV.

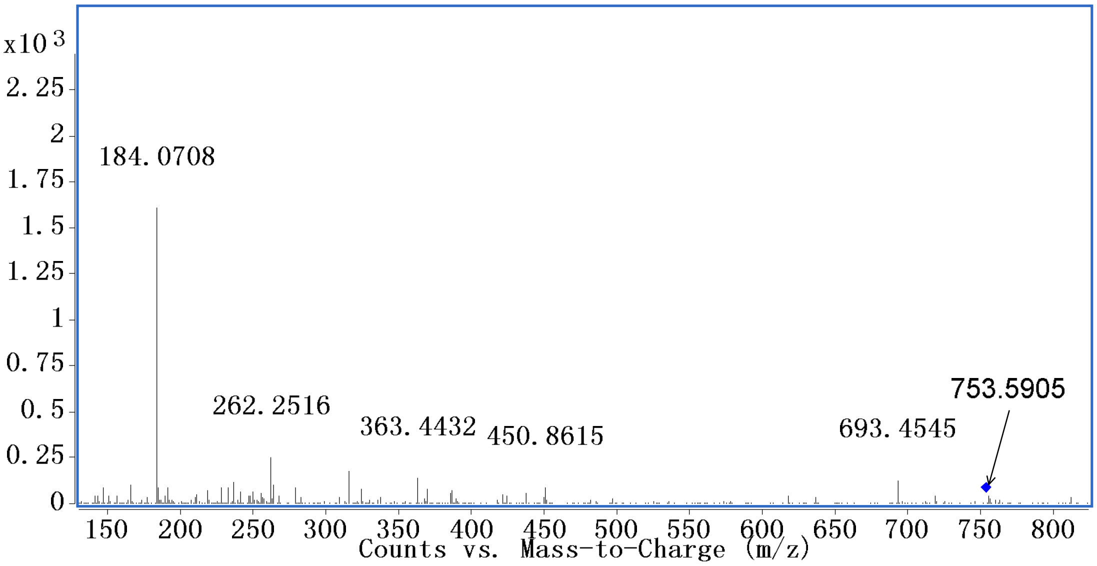

No.112 **SM (d18:2/20:2)**: $[M+H]^+$ 753.5905, [So (d18:2)-$2H_2O$+H]$^+$ 262.2516, [phosphocholine+H]$^+$ 184.0708, the collision energy was set as 40eV.

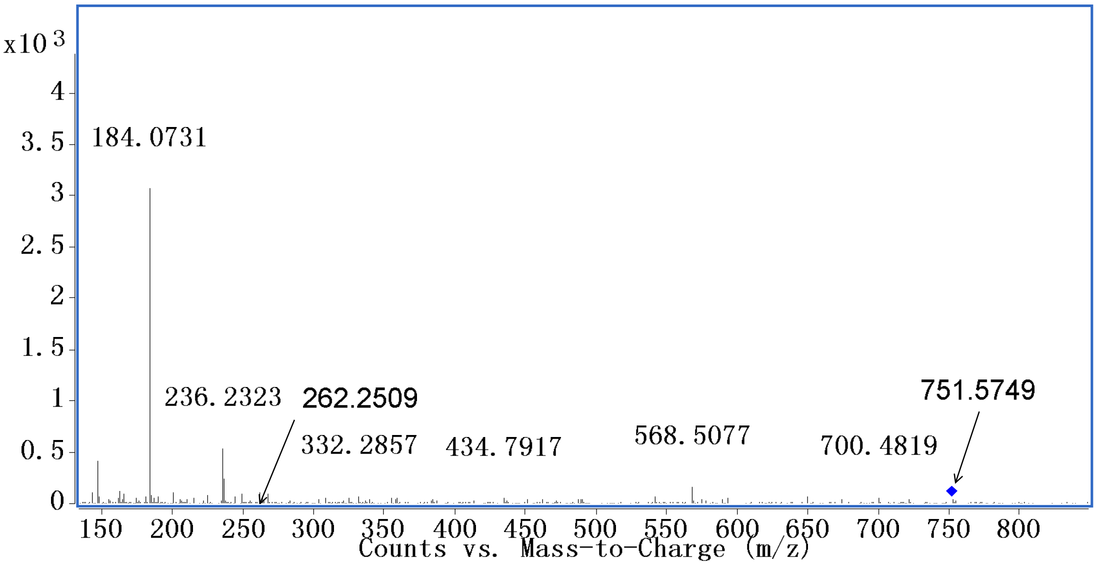

No.113 **SM (d18:2/20:3)**: $[M+H]^+$ 751.5749, [So (d18:2)-$2H_2O$+H]$^+$ 262.2509, [phosphocholine+H]$^+$ 184.0731, the collision energy was set as 40eV.

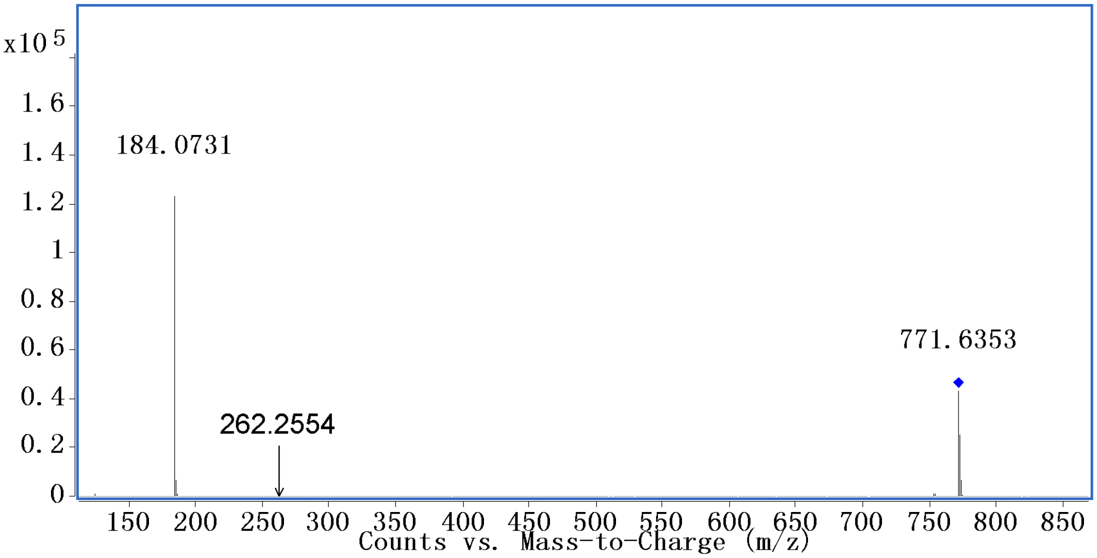

No.114 **SM (d18:2/21:0)**: $[M+H]^+$ 771.6353, [So (d18:2)-$2H_2O$+H]$^+$ 262.2554, [phosphocholine+H]$^+$ 184.0731, the collision energy was set as 20eV.

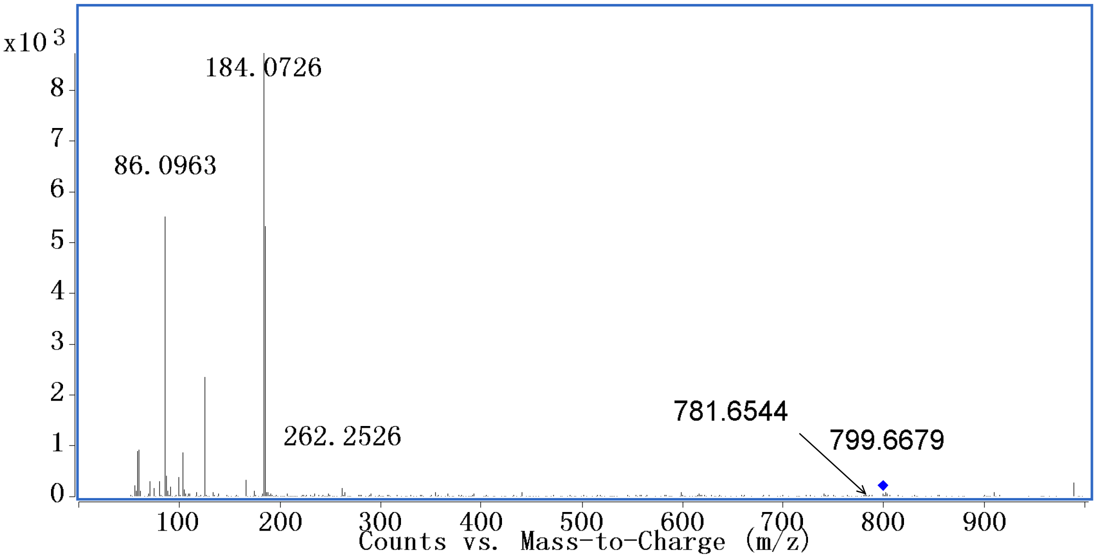

No.115 **SM (d18:2/23:0)**: $[M+H]^+$ 799.6679, [M-H_2O+H]$^+$ 781.6544, [So (d18:2)-$2H_2O$+H]$^+$ 262.2526, [phosphocholine+H]$^+$ 184.0726, the collision energy was set as 40eV.

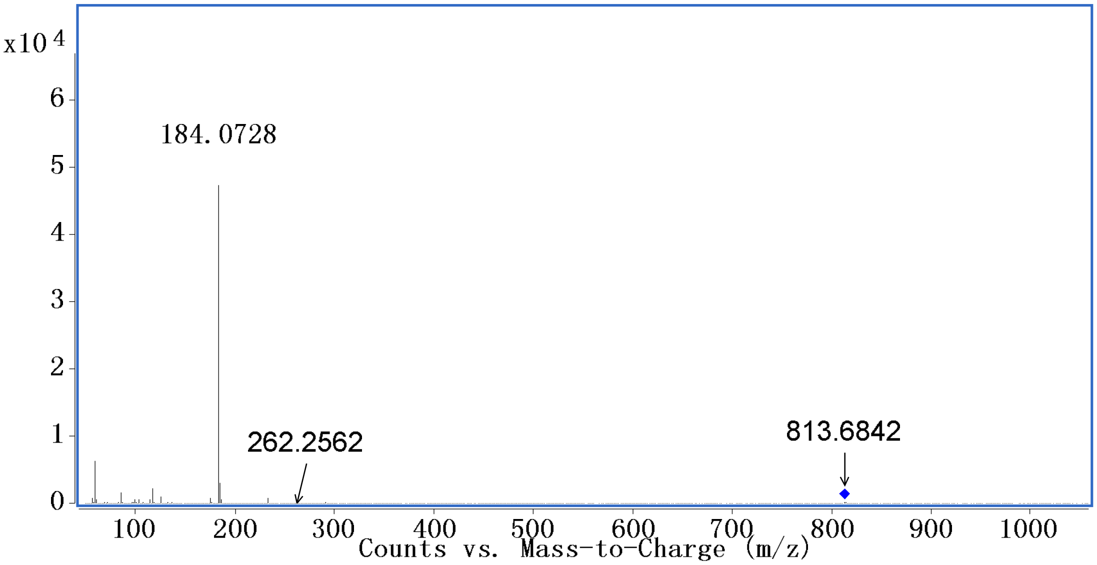

No.116 **SM (d18:2/24:0)**: $[M+H]^+$ 813.6842, [So (d18:2)-$2H_2O$+H]$^+$ 262.2562, [phosphocholine+H]$^+$ 184.0728, the collision energy was set as 40eV.

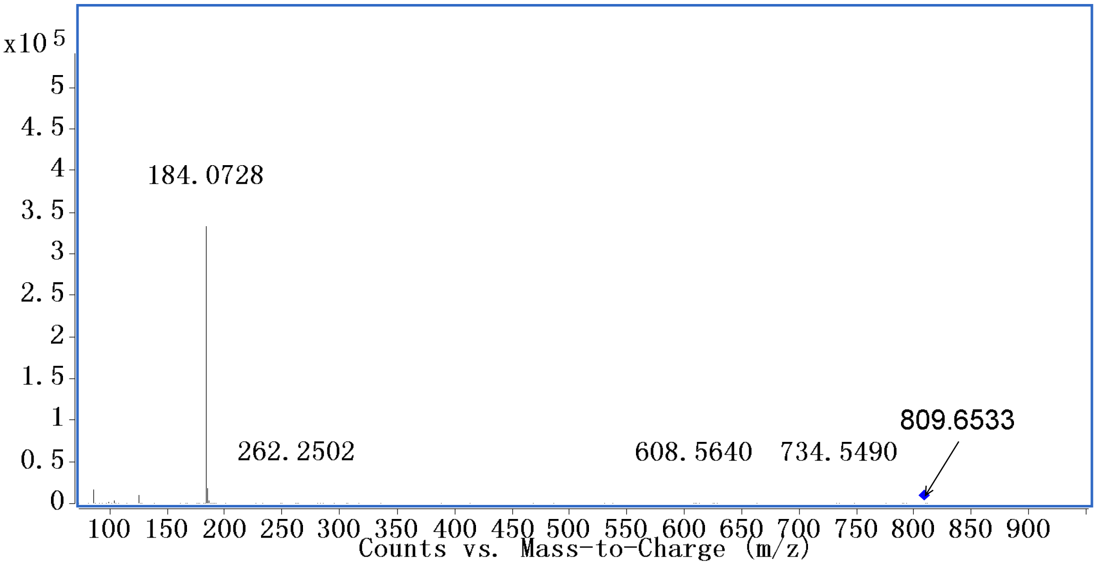

No.117 **SM (d18:2/24:2)**: $[M+H]^+$ 809.6533, [So (d18:2)-$2H_2O$+H]$^+$ 262.2502, [phosphocholine+H]$^+$ 184.0728, the collision energy was set as 40eV.

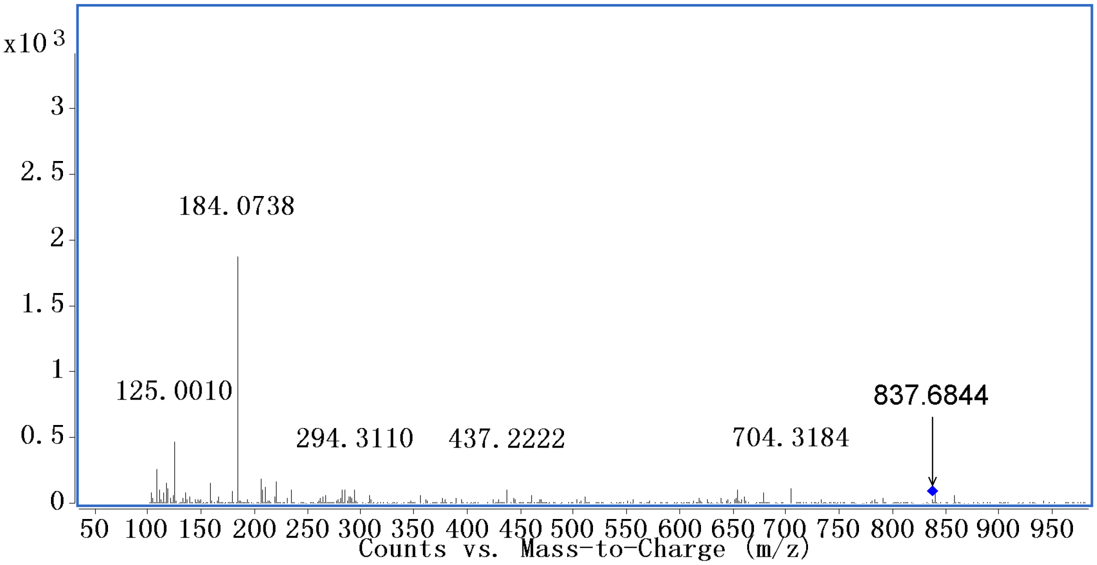

No.118 **SM (d20:0/24:4)**: $[M+H]^+$ 837.6844, [Sa (d20:0)-$2H_2O$+H]$^+$ 294.3110, [phosphocholine+H]$^+$ 184.0738, the collision energy was set as 40eV.

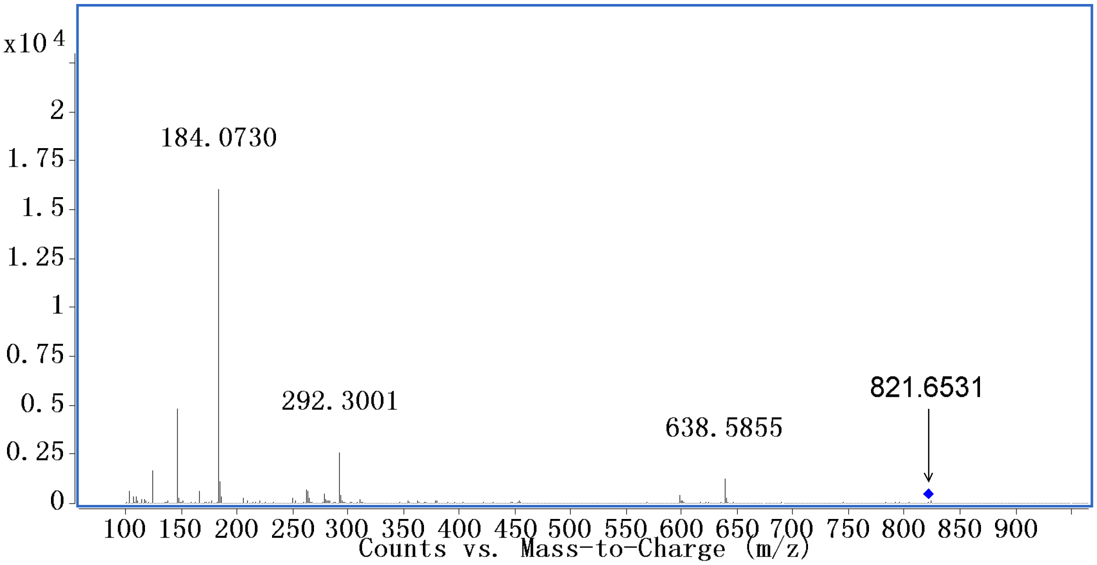

No.119 **SM (d20:1/23:4)**: $[M+H]^+$ 821.6531, [So (d20:1)-$2H_2O$+H]$^+$ 292.3001, [phosphocholine+H]$^+$ 184.0730, the collision energy was set as 40eV.

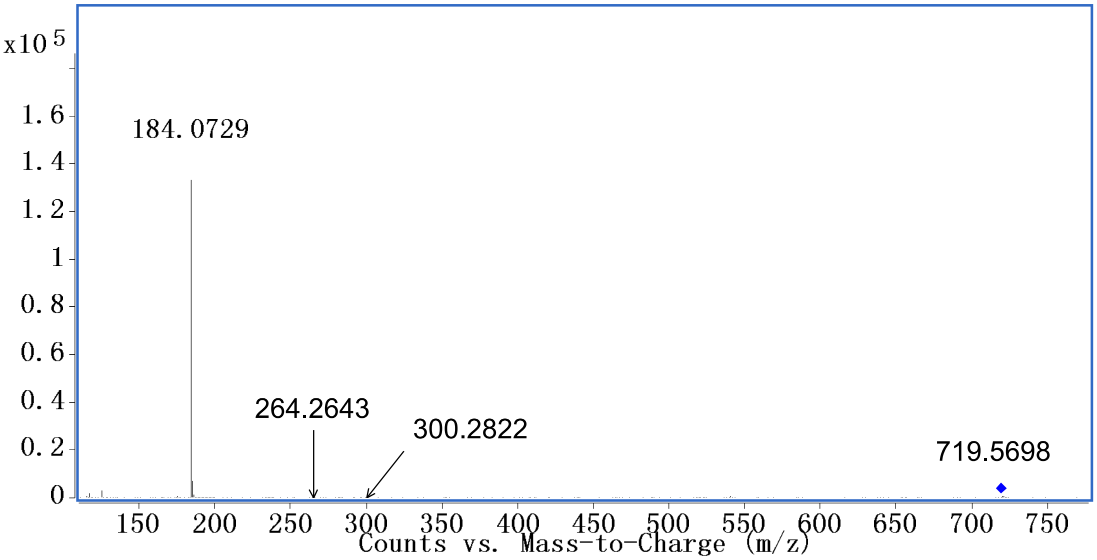

No.120 **SM (t18:0/16:1)**: $[M+H]^+$ 719.5698, [Sa (t18:0)-H_2O+H]$^+$ 300.2822, [Sa (t18:0)-3H_2O+H]$^+$ 264.2643, [phosphocholine+H]$^+$ 184.0729, the collision energy was set as 40eV.

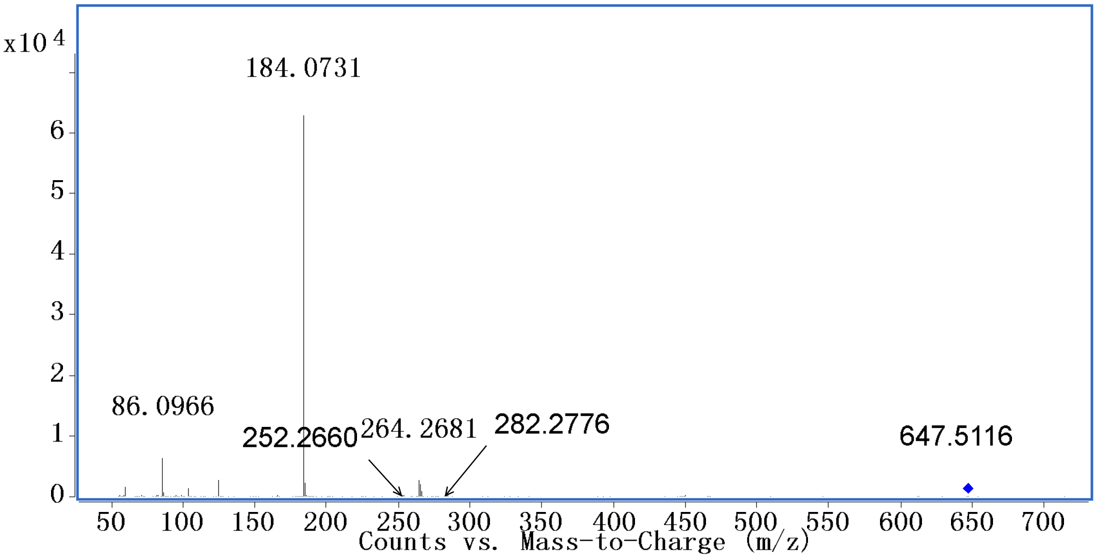

[IS-8] **SM (d18:1/12:0)**: $[M+H]^+$ 647.5116, [So (d18:1)-H_2O+H]$^+$ 282.2776, [So (d18:1)-2H_2O+H]$^+$ 264.2681, [So (d18:1)-H_2O-HCHO+H]$^+$ 252.2660, [phosphocholine+H]$^+$ 184.0731, the collision energy was set as 40eV.

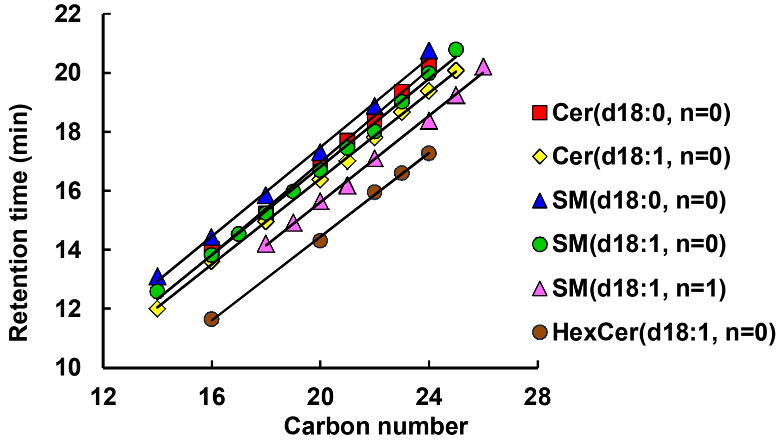

Figure S2. Linear regression models constructed by plotting carbon number vs. retention time for SPLs. X-axis stands for the carbon number in fatty acid chain; Y-axis stands for retention time; n stands for the degree of unsaturation of fatty acid chain. Cer, ceramide; SM, sphingomyelin; HexCer, hexosylceramide.

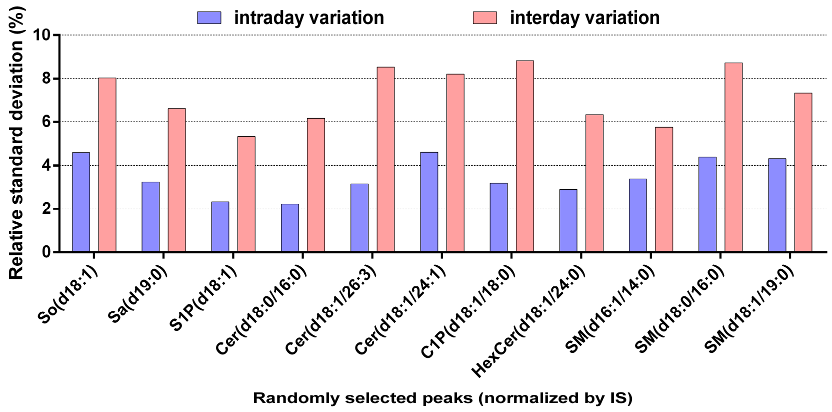

Figure S3. Intra- and inter-day RSDs of randomly selected SPLs in rat serum sample. Peak areas were normalized to the corresponding IS. The SPLs were randomly selected, taking SPLs of varying abundance and various SPL subclasses. So, sphingosine; Sa, sphinganine; S1P, sphingosine-1-phosphate; Cer, ceramide; C1P, ceramide-1-phosphate; HexCer, hexosylceramide; SM, sphingomyelin.

Table S1. Linearity and sensitivity testing results of 8 IS spiked in rat serum extracts.

spiked standards[1]	calibration equations[2]	linear range (μM)	R^2	LOD (nM)	LOQ (nM)
So(d17:1)	y = 2008 x + 12655	0.0167-25	0.998	2.20	7.33
Sa(d17:0)	y = 2599 x + 15445	0.0167-25	0.997	1.13	3.77
S1P(d17:1)	y = 3393 x + 13388	0.0167-16.67	0.997	2.32	7.73
Cer(d18:1/12:0)	y = 3043 x + 17356	0.0167-16.67	0.995	1.27	4.22
C1P(d18:1/12:0)	y = 505 x + 466	0.0167-16.67	0.998	2.00	6.67
GlcCer(d18:1/12:0)	y = 1248 x + 733	0.0167-16.67	0.998	2.80	9.33
LacCer(d18:1/12:0)	y = 981 x + 1094	0.0167-16.67	0.998	1.53	5.10
SM(d18:1/12:0)	y = 5599 x + 3024	0.033-25	0.996	0.11	0.36

[1] So, sphingosine; Sa, sphinganine; S1P, sphingosine-1-phosphate; Cer, ceramide; C1P, ceramide-1-phosphate; GlcCer, glucosylceramide; LacCer, lactosylceramide; SM, sphingomyelin.

[2] y = integrated peak area and x = concentration of corresponding spiked IS.

Table S2. Recovery testing results of 8 IS spiked in rat serum extracts.

spiked standards[1]	low con.[2]		medium con.[1]		high con.[1]	
	recovery (%)	RSD (%)	recovery (%)	RSD (%)	recovery (%)	RSD (%)
So(d17:1)	98.5	7.6	97.5	12.2	94.3	5.0
Sa(d17:0)	95.3	12.5	96.2	8.2	94.1	5.3
S1P(d17:1)	97.8	7.7	98.3	8.4	96.5	6.1
Cer(d18:1/12:0)	96.5	5.8	93.2	6.5	108.5	6.7
C1P(d18:1/12:0)	96.3	6.1	94.0	7.8	101.2	7.0
GlcCer(d18:1/12:0)	102.4	9.8	91.3	6.8	102.9	6.3
LacCer(d18:1/12:0)	101.6	10.1	93.6	8.3	98.6	5.4
SM(d18:1/12:0)	98.2	4.5	99.4	5.5	100.5	4.8

[1] So, sphingosine; Sa, sphinganine; S1P, sphingosine-1-phosphate; Cer, ceramide; C1P, ceramide-1-phosphate; GlcCer, glucosylceramide; LacCer, lactosylceramide; SM, sphingomyelin.

[2] Serum samples spiked with low (5 pmol), medium (50 pmol), and high (500 pmol) levels of IS.
